# Select Dietary Supplement Ingredients for Preserving and Protecting the Immune System in Healthy Individuals: A Systematic Review

**DOI:** 10.3390/nu14214604

**Published:** 2022-11-01

**Authors:** Cindy Crawford, LaVerne L. Brown, Rebecca B. Costello, Patricia A. Deuster

**Affiliations:** 1 Consortium for Health and Military Performance, Department of Military and Emergency Medicine, F. Edward Hebert School of Medicine, Uniformed Services University, Bethesda, MD 20817, USA; 2 Henry M. Jackson Foundation for the Advancement of Military Medicine, Bethesda, MD 20817, USA; 3 Office of Dietary Supplements, National Institutes of Health, Bethesda, MD 20817, USA

**Keywords:** dietary supplements, immune health, systematic review, resilience, acute respiratory infection

## Abstract

Immune health products represent approximately 10% of all US dietary supplement sales. Claims made on products to support or boost the immune system are attractive to the otherwise healthy consumer who may or may not be experiencing certain life stressors. The purpose of this systematic review is to critically evaluate the purported benefits and/or potential harms of select dietary supplement ingredients frequently listed on the labels of products having immune health or related market claims. With a focus on resilience, research questions were related to whether dietary supplement ingredients are efficacious in preserving and protecting immune health in healthy individuals; and when faced with a stressor, whether taking a supplement prophylactically can assist in maintaining health and resisting or bouncing back more quickly. Thirty-nine randomized controlled studies involving populations including children, adults and seniors exposed to stressors, such as air travel, intense exercise, academic stress, and/or exposure to winter weather, met eligibility criteria. The studies included eight of the 27 supplement ingredients identified through a market-driven scoping review. Those ingredients used in single ingredient products were echinacea, elderberry, garlic, vitamin A, vitamin C, vitamin D, vitamin E, and zinc. Whereas some studies may point to evidence for benefit, specific gaps preclude the authors from making firm statements with regard to the overall evidence-base for these products and ingredients and in answering the research questions. As we move toward a vision of health promotion and resilience rather than a sole focus on disease prevention and treatment, further work in this area of dietary supplements is of utmost importance.

## 1. Introduction

The general public has increased their use of dietary supplements since the start of the COVID-19 pandemic. Dietary supplements purported to mediate immune function for disease prevention, health maintenance, and resilience outcomes and with claims to “boost” the immune system, are emerging in new products as well as previously marketed products. Cold, flu and immunity supplement sales reported by the Nutrition Business Journal (NBJ) climbed to almost $6 billion by the end of 2020, from $3.4 billion US dollars in 2019, with immune health representing approximately 10% of all US supplement sales [1,2,3,4].

Dietary supplements, by definition are not meant to “treat, diagnose, cure or prevent diseases” but “are intended to add to or supplement the diet and are different from conventional food” [5]. However, existing research tends to focus on specific disease states and populations rather than health maintenance, optimizing performance, and resilience. Most recently, the Office of Dietary Supplements (ODS) at the National Institutes of Health (NIH) established the Trans-NIH Resilience Working Group, to “seek fundamental knowledge about the nature and behavior of living systems and the application of that knowledge to enhance health, lengthen life and reduce illness and disability through the study of resilience”. “Resilience encompasses the capacity to resist, adapt to, recover, or grow from a challenge” [6]. For most people interested in taking dietary supplements, and in line with the Food and Drug Administration (FDA) definition for dietary supplements and how they are supposed to be marketed, resilience outcomes are important to examine. Claims made for “improving” the immune response or protecting immune health are attractive to those individuals that are otherwise healthy or perhaps experiencing certain life stressors or challenges. The number of products to potentially choose from seem immeasurable.

The purpose of this systematic review is to critically evaluate and highlight the scientific knowledge of the purported benefits and/or potential harms of select dietary supplement ingredients frequently listed as contained in these products with market claims associated with improved immune response or protecting immune health. This was deemed important in order to provide accurate, evidence-based and timely information to the public. The public will benefit by our highlighting the science behind products, ingredients and claims [benefits and harms]. Furthermore, the scientific evidence will be used to inform strategic decisions regarding future research initiatives in the area of health maintenance and resilience.

## 2. Materials and Methods

### 2.1. Scoping Review for Selection of Dietary Supplement Ingredients

The authors took a market driven approach for the selection and evaluation of dietary supplement ingredients coming into the market and/or forecasted to contribute to increased sales in the immune health dietary supplement market. Authors collated information from four sources: (1) products and ingredients with “immune” claims found in the Dietary Supplement Label Database (DSLD); (2) the NBJ 2020 Immune System Report’s top 20 ingredients ranked by immunity sales [7]; (3) a recent publication on “immune boosting” ingredients and dietary supplements that appeared in greater than 10% of webpage advertisements based on Google analytics during the first and second wave of COVID [8]; and (4) a published article on popular dietary supplements based on Google search trends related to immunity during the COVID March 2020 outbreak [9]. Full details can be found in Supplementary File S1. Ultimately, 27 unique dietary supplement ingredients considered to be frequently contained in dietary supplement products with claims related to immune function were selected. Some of the most common claims identified from these products found on DSLD and Amazon.com consisted of “immune support”, “supports healthy immune system”, “bolsters the immune system”, “natural immune booster”, and “immune defense”.

Findings from these activities were presented to a diverse panel of research experts brought onto the project to help define the research question(s) and scope for systematic review. The panel was also engaged at the conclusion of the project to help make recommendations about next steps for research based upon the evidence presented.

The following research questions were ultimately developed for systematic review:

1. Are select dietary supplement ingredients efficacious in preserving and protecting the immune system in otherwise healthy individuals?

2. Are select dietary supplement ingredients efficacious in preserving and protecting the immune system in otherwise healthy individuals experiencing a stressor?

3. Do individuals exposed to stressors (e.g., lack of sleep, intense exercise, environmental/wintertime) recover more quickly if they have taken a select dietary supplement ingredient prior to an acute respiratory tract infection, than those not taking a dietary supplement, or as compared to placebo?

### 2.2. Search Strategy and Selection Criteria for Systematic Review

The authors searched PubMed, CINAHL, PsycInfo and EMBASE for randomized controlled trials (RCTs) involving humans published from 2001 through 7 October 2021. Supplementary File S2 details the search strategy executed. Reference lists of all eligible trials were also examined as other search sources. The authors chose the starting point of 2001 for the evidence generation in an attempt to include ingredients and products likely representative of the market today with current claims for immune health.

All citations retrieved from the searches were uploaded into the systematic review platform, Covidence, for title and abstract screening according to predefined eligibility criteria (Table 1). Primary outcomes for this systematic review included incidence of infection, severity, duration of symptoms for events that did occur, and any reported adverse events. An infection for this systematic review was defined as that related to an acute respiratory infection to include symptoms such as the common cold, cough, congestion, fever, body aches and influenza. The eligibility criteria were constructed in such a way to be consistent with Food and Drug Administration’s (FDA) definition for dietary supplements and by marketed health claims on products [5,10]. Two reviewers screened titles and abstracts accordingly in duplicate (CC, CB). A protocol for this systematic review is registered in Prospero under CRD42021288766.

### 2.3. Data Extraction and Quality Assessment

Data were extracted from each eligible study to describe the basic characteristics including the participants, any stressors they were experiencing or exposed to at the time of the study, the dietary supplement product and formulation, amount and duration of time taking the product, and associated outcomes reported, as well as the funding source. Two reviewers assessed risk of bias independently by using the Scottish Intercollegiate Guidelines Network—SIGN 50 for RCTs [11].

### 2.4. Data Synthesis and Analysis

Studies were grouped by their specific dietary supplement ingredient, by children, adults, and seniors, and whether the participants were experiencing a stressor as reported in the publications. Where data were available on the number of participants experiencing at least one infection, the number of participants and sample sizes in each group were extracted and a risk ratio (RR) was calculated for each included study. When data were available on any adverse events reported, a risk difference (RD) was calculated based on the numbers in each group. Where data were available on severity of symptoms, means, standard deviations and sample sizes in each group were extracted and a standardized mean difference (SMD) calculated. For duration of symptoms, the mean number of days, standard deviations and number of events were extracted and a mean difference (MD) in days calculated. Data were first entered into Comprehensive Meta-Analysis for calculations and subsequently into REVMAN 5.4 for creating forest plots. A negative effect size indicates benefit of the dietary supplement ingredient over that of placebo for MD, RD and SMDs. No totals for overall effects or sub-analyses were pooled for incidence, severity, or duration outcomes, due to the heterogeneity across dietary supplement products, amounts, formulations and the different populations and stressors studied. The individual calculations were done so data could be presented consistently across studies with a forest plot for visual comparison of results across studies, stressors, and products. This was carried out for easier interpretation rather than relying upon statistical *p*-values as reported in the individual studies according to varying measures and metrics.

All studies, regardless of whether data were available for extraction and analysis, are narratively described. The authors chose not to make any assumptions about data or transform any statistics reported to fit these calculations, except for transforming standard errors (SEM) to standard deviation (SD) where required. When data could not be used, the data reported in the original publication were recorded to describe the outcome as well as for all secondary outcomes related to biomarkers and other resilience type outcomes from studies (Supplementary File S2).

Due to the variations across the products included and because the authors selected a starting point of 2001 for the evidence, the authors chose not to use the Grading of Recommendations, Assessment, Development and Evaluation (GRADE) approach to assign a formal “GRADE” for the overall confidence in any effect estimate. Instead, we narratively describe the evidence along these criteria [risk of bias, indirectness, inconsistency, imprecision] and for benefits and risks across studies related to the research questions for systematic review [12,13].

To gain further insight into potential adverse events beyond those reported in efficacy trials, the authors searched for published case reports in PubMed, reviewed Natural Medicines database “adverse effects” sections of dietary ingredient professional monographs, and examined the Center for Food Safety and Applied Nutrition Adverse Event Reporting System (CAERS) database (January 2014–March 2020), which contains reports on dietary supplement product “suspect” adverse events submitted to FDA.

## 3. Results

Twenty-seven ingredients were identified through the market driven approach for the selection of dietary supplement ingredients for systematic review (Supplementary File S1). The search strategy yielded 3928 unique publications once duplicates were removed across databases (Supplementary File S2). Thirty-nine RCT studies (across 43 publications) met eligibility criteria including eight dietary supplement ingredients: echinacea (N = 6; n = 1708 subjects) [14,15,16,17,18,19], elderberry (N = 1; n = 312) [20], garlic (N = 2; n = 266) [21,22,23], vitamin A (N = 2; n = 1719) [24,25], vitamin C (N = 3; n = 237) [26,27,28], vitamin D (N = 18; n = 19,309) [29,30,31,32,33,34,35,36,37,38,39,40,41,42,43,44,45,46,47,48], vitamin E (N = 1; n = 652) [49,50] and zinc (N = 6; n = 1445) [51,52,53,54,55,56], involving populations including children, adults and seniors, exposed to stressors described as stressful air travel, intense exercise, academic stress, exposure to winter months, environmental stressors such as poor living environments or where deficiency in certain nutrients is prevalent, and subjects inoculated with a virus. After title and abstract screening, the authors decided to exclude prebiotic and probiotic studies from this current review due to complexities in which these ingredients are categorized/defined in terms of dietary supplements vs. food sources. [57] In addition, although studies on mushrooms were identified from the search strategy, they were primarily on beta-glucans either isolated from *Pleurotus ostreatus* (oyster mushroom) and presented as a combination product including vitamin C [58,59,60] (and currently off the market according to DSLD), or comparing different sources of beta-glucans. [61] These studies were excluded, after careful examination, for being combination products. Furthermore, no published studies were identified meeting the eligibility criteria for astragalus, calcium, ginger, goji, goldenseal, holy basil, licorice, magnesium, melatonin, mangosteen, noni, rose hip, slippery elm, turmeric/curcuma, selenium, vitamin B or vitamin K as single ingredient products for immune health. Of the 39 included studies, eight were industry sponsored and eight did not disclose the granting agency or sponsorship.

**Figure 1:**
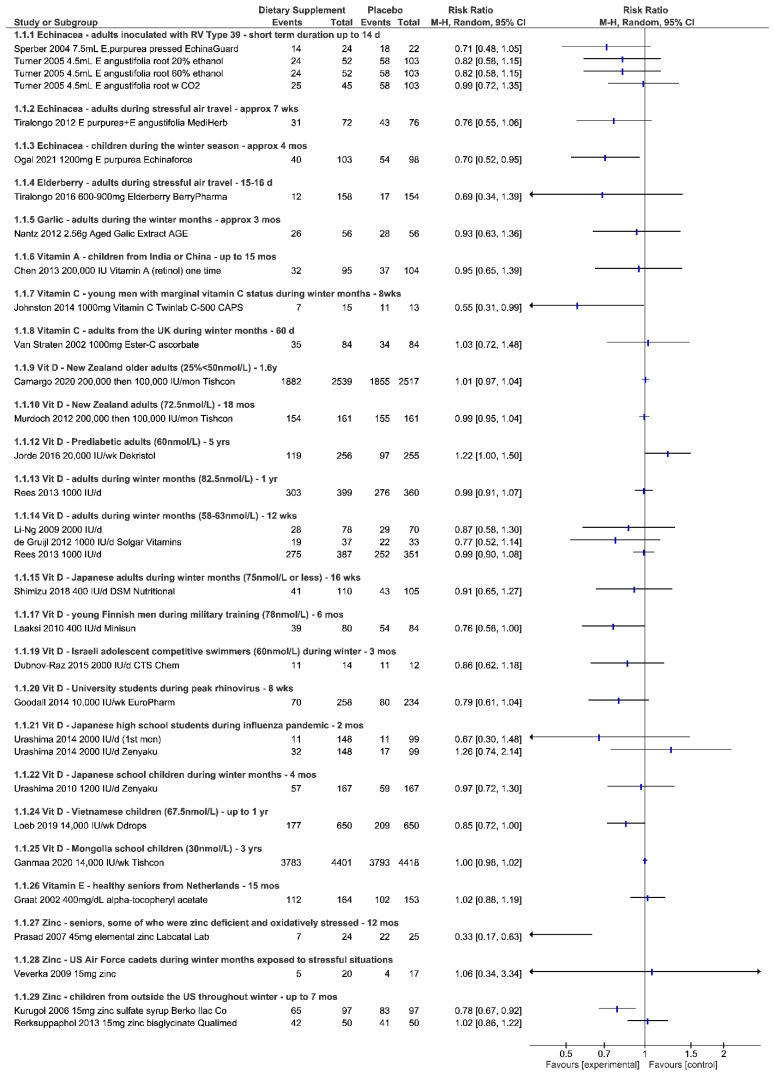
Analysis 1.1 Number of persons experiencing at least one infection.

**Figure 2:**
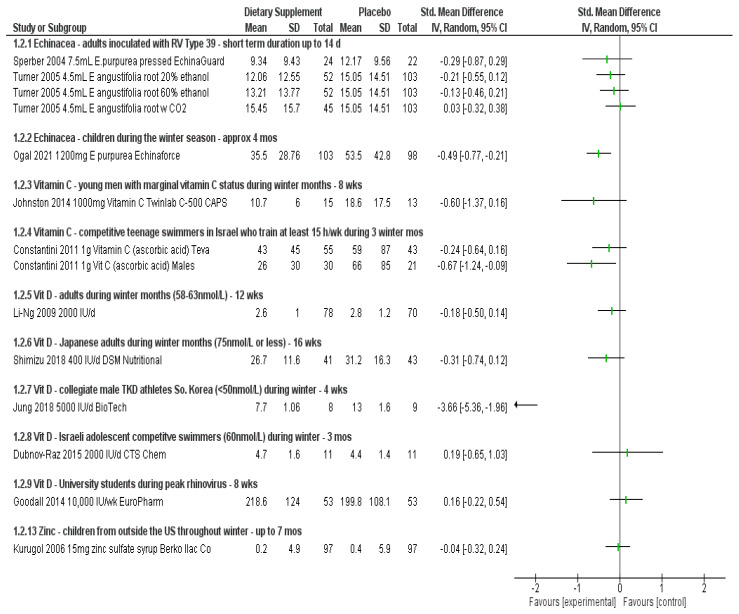
Analysis 1.2 Severity of symptoms as reported by participants.

**Figure 3:**
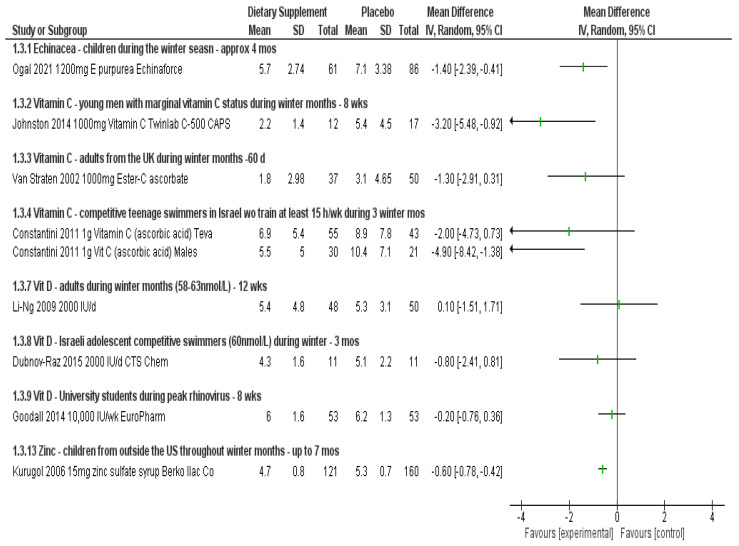
Analysis 1.3 Duration of symptoms per event experienced by participants.

**Figure 4:**
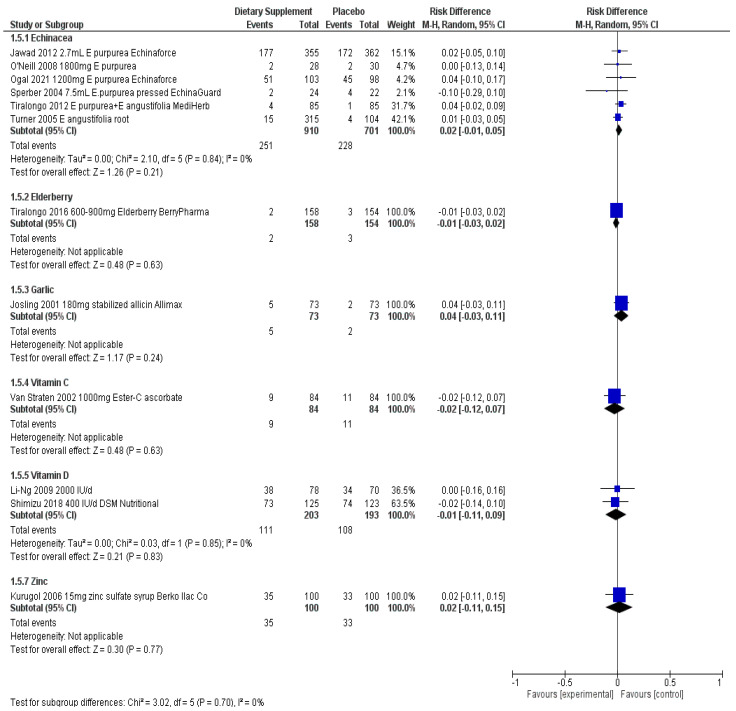
Analysis 1.4 Adverse events experienced by participants.

Results below describe the evidence for each ingredient separately for efficacy and safety in an attempt to understand the evidence for answering the three research questions posed above. Figure 1, Figure 2, Figure 3 and Figure 4 visually show the analyses across primary outcomes where data were available from studies. Table 2 summarizes the evidence collectively and research considerations/gaps discovered. Supplementary File S2 details all data related to the individual studies characteristics, quality, outcomes, and adverse event reporting.

### 3.1. Echinacea

Echinacea species are perennials closely related to sunflowers and ragweed. Extracts of the leaf, flower, and root of some species, most commonly, *E. angustifolia* and *E. purpurea*, can be found in dietary supplement products promoted to prevent and/or treat the common cold and other respiratory tract infections. Common claims on these products include “year-round herbal supplement for immune support”, “immunity boosting”, and “immune defense”. According to the 2020 NBJ Report, Echinacea represented 4.3% in 2019 of the $3.4 billion in cold, flu and immunity supplement sales, with sales projected to increase exponentially through 2020s [1,2].

Six studies met the eligibility criteria for review. One study was representative of 203 children aged 4–12 years who took EchinaForce (*E. purpurea* extract) 1200 mg/day during the winter months for approximately four months, with a one-week break after the first two months. [15] Five studies involved healthy adults exposed to various stressors; two of these studies involved participants (n = 485) instructed to take different formulations of Echinacea (one using 7.5 mL/day EchinaGuard (from *E. purpurea* in a 22% alcohol base) [17] and the other using three different preparations of 4.5 mL/day of *E. angustifolia* extracted with either carbon dioxide, 60% or 20% ethanol); [19] both studies were for a short-term duration of up to 14 days in length. At day seven, participants from these two studies were inoculated with Rhinovirus Type-39. Another two studies (n = 845) reported stressors during the winter months (i.e., a natural exposure to cold/influenza); specifically, participants were either medical center employees, [16] or university students who typically experience ≥2 colds per year but were healthy at the start of the study. [14] Both studies used *E. purpurea*, one reporting on 2.7 mL/day EchinaForce drops (2400 mg Echinacea) over the course of four months [14] and the other disclosing the product simply as “capsules” consisting of 1800 mg/day for eight weeks. [16] Finally, Tiralongo et al. [18] included 175 adults during and after traveling that included long-haul intercontinental flights throughout a five week period. Participants took MediHerb brand Echinacea tablets which contains *E. purpurea* and *E. angustifolia*. A matching control, considered as placebo, was sufficiently described in most of these studies. Characteristics of all studies are detailed in Supplementary File S2.

The methodological quality of these studies was overall “acceptable” according to SIGN-50 criteria for risk of bias with one study being rated as “low quality” [17]. Criteria not adequately described included allocation concealment and the randomization process; selective reporting appeared in some publications. As per the actual formulation of Echinacea investigated, two of the studies did not disclose the product name or manufacturer of the product, [16,19] five did not describe the method of authentication of the raw material or a description of any special testing/purity testing undertaken [15,16,17,18,19]. Research into what the active constituents of various species of Echinacea might be ongoing [62].

#### 3.1.1. Outcomes

Trials reported the number of participants experiencing an infection by using the Wisconsin Upper Respiratory Symptom Survey (WURSS-44), the Jackson scale or other self-report methods. No statistical difference was detected in the three individual studies [17,18,19] reporting data for the risk of experiencing an infection for adults exposed to various stressors and taking various formulations of Echinacea, prophylactically as compared to placebo. Studies were consistent however in showing less risk (RR 0.71 to RR 0.82; up to 29%) in developing symptoms when taking Echinacea as compared to placebo (Figure 1).

Two trials involving adults [17,19] demonstrated very small to no effect on overall symptom severity throughout the course of the trial when using symptom severity self-report scales (SMD −0.29 to 0.03) (Figure 2). Tiralongo reported “borderline significantly lower respiratory symptom scores compared to placebo [during travel]” [18]. O’Neill reported that the placebo group consistently reported symptoms more often during any given week than the Echinacea group, but “none of these differences were statistically significant” [16].

Outcomes related to the duration of symptoms for events that did occur throughout the trial were only reported in two of those involving adults. Jawad reported, “The difference of cumulated events (episodes and episode days) between the treatment groups each of 26% reached statistical significance for episode days (*p* < 0.05, chi-square test)” [14] whereas O’Neill reported the “Median number sick days (the sum of the most prevalent symptom each week)” was 9 days in Echinacea vs. 14 days in control; total of 56 days; (z = 0.42; *p* = 0.67) [16].

There was no difference in the risk of experiencing an adverse event when taking various Echinacea products vs. placebo controls used in the studies involving adults; (Figure 4) RD 0.02 (95% CI, −0.01, 0.05; *p* = 0.24).

Ogal et al. was the only study eligible for review involving children [15]. Using the same statistical analysis techniques as for those adults, a 30% less risk (RR 0.70 (95% CI, 0.52, 0.95; *p* = 0.02) of developing symptoms was found when taking *E. purpurea*. A moderate effect was found in the severity of symptoms, in favor of Echinacea (SMD −0.49 (95% CI, −0.77, −0.21; *p* = 0.001); there was no difference in risk of adverse events between treatment and control (RD 0.04 (95% CI, −0.10, 0.17; *p* = 0.61). The data also suggest that children taking Echinacea had less confirmed laboratory infections (RR 0.75 (95% CI, 0.61, 0.93; *p* = 0.008) than controls. Like Jawad [14], this study also used the product EchinaForce, and similarly reported a significant reduction in duration, with a mean of 1.4 days less compared to placebo (MD −1.40 (95% CI, −2.39, −0.41; *p* = 0.008) (Figure 1, Figure 2, Figure 3 and Figure 4).

Laboratory evidence of infection and biomarkers are reported in Supplementary File S2. No studies reported on other outcomes of well-being, such as sleep quality, mood, or activity.

#### 3.1.2. Adverse Events

As reported above, studies show no difference in the risk of experiencing an adverse event when comparing Echinacea to a placebo control. Reported events included symptoms of sleeplessness and severe oral aphthous ulcers, which resolved spontaneously while receiving treatment (evaluated as likely not to be related to treatment), gastrointestinal side effects (e.g., diarrhea), as well as other allergic/hypersensitivity reactions (e.g., rash, urticaria) experienced in both the placebo and Echinacea groups. Natural Medicines reports that generally, “orally, Echinacea is well-tolerated” and that the “most common adverse effects include abdominal pain, constipation, diarrhea, heartburn, nausea and vomiting, rashes and stomach upset. Severe [rare] allergic reactions and hepatitis have been reported”. There is no known tolerable upper limit for Echinacea and doses vary widely across commercial products and various extracts. In addition, the active constituents are not currently known. There have been approximately 26 CAERS reported adverse event incidences documented between 2014 and 2020. Supplementary File S2 details adverse events on record and 10 published case reports.

#### 3.1.3. Quality of the Evidence

Collectively, these studies are not directly related to each other and consist of different populations experiencing different life stressors and taking different formulations/amounts for varying durations. On their own as individual studies, they show few statistically significant results but do show overall consistency in positive trending results. Power to detect a meaningful difference may be a factor influencing the results, and thus imprecision is of concern. The largest trial involving adults [14] and most recent study among children [15] both reported significant results in favor of Echinacea. Both were also using the same product brand supplied by the same manufacturer in the studies, throughout the winter months.

### 3.2. Elderberry

Elderberry is the berry of the black elder tree, or *Sambucus nigra*, which contains a number of antioxidants. In dietary supplements, elderberry is typically included as an extract or juice concentrate of the whole fruit, when marketed for immune health. Common product claims include “cold and flu relief”, “year-round immune support” and “supports wellness and vitality”. According to the recent NBJ report, elderberry represented 7.2% of the cold, flu and immunity supplement sales in 2020, which reached close to $6 billion [2].

One study met the review’s eligibility criteria and was included in the review [20]. A total of 312 healthy long-flight airplane passengers who were traveling to an overseas destination were given 300 mg capsules of proprietary membrane filtered elderberry (*Sambucus nigra* L., Haschberg variety; BerryPharma Brand); specifically, adult participants were asked to take 600 mg/day for 10 days before travel and 900 mg/day for five days (e.g., one day before leaving home until 4–5 days after arriving at the destination). A full description of the study’s characteristics is detailed in Supplementary File S2.

This study’s overall methodological quality was considered “high quality” according to SIGN-50 criteria for risk of bias. Although the elderberry supplement was described, several pieces of information regarding the actual formulation of the supplement were missing: method of authentication, chemical fingerprint, and purity testing.

#### 3.2.1. Outcomes

This trial used the Jackson score to document the number of participants developing a well-defined cold; it was not statistically different between the elderberry and placebo groups; (RR 0.69 (95% CI, 0.34, 1.39; *p* = 0.298) (Figure 1). The study reported that participants suffering cold episodes while taking elderberry would experience a “2-day shorter duration of a cold (4.75 days vs. 6.88 days) and a lower symptom severity (mean 21 vs. 34)” in comparison to those in the placebo group suffering from a cold. Standard deviations were not reported in the publication. The authors also examined other outcomes such as quality of life measures by using SF-12 and the Perceived Stress Scale to evaluate whether subjects were stressed during the trial Supplementary File S2.

#### 3.2.2. Adverse Events

There was no difference in the risk of experiencing an adverse event throughout the trial. A risk difference was calculated as RD −0.01 (95% CI, −0.03, 0.02; *p* = 0.632) (Figure 4). Reported adverse events included cold-like symptoms and fatigue, however, a causal relationship between elderberry and the events could not be established. Natural Medicines reports that elderberry is generally well-tolerated, with common adverse events being nausea, vomiting, diarrhea, weakness, dizziness, numbness, and stupor. Such events may be due to ingestion of raw/unripe elderberries and/or their plant parts, and may be avoided by cooking the elderberry and subsequently eliminating the toxin. The BerryPharma used in the included study is a proprietary elderberry extract. According to Natural Medicines, “Elderberry fruit extracts have most often been used in doses up to 1200 mg daily for 2 weeks or up to 500 mg daily for up to 6 months in adults”. There is no known tolerable upper limit for elderberry. There have been approximately 27 CAERS adverse events reported from 2014 to 2020. Supplementary File S2 details adverse events on record and one published case report.

### 3.3. Garlic

Garlic (*Allium sativum*) is a plant related to onions, leeks and chives, with a long history of culinary as well as medicinal use. People commonly use garlic for high blood pressure and cholesterol. According to FDA, garlic is generally recognized as safe (GRAS) for use. In dietary supplements, garlic is commonly marketed to boost the immune system, acts as a “cold blaster” and offers support for the “cold and flu season”. The interest in and use of garlic has increased substantially since the start of the COVID-19 pandemic [8,9].

Two studies met the review’s eligibility criteria and were included in the review. Josling et al. (2001) [21] reported on 146 adult healthy volunteers from London who were asked to take Allimax containing 180 mg of stabilized allicin per day for 12 weeks during the winter months. The second study [22,23] involved 120 healthy men and women recruited from the University of Florida and surrounding community who took Aged Garlic Extract (AGE) powder (4 capsules per day: 2.56 g) for 90 days throughout the cold and flu season which coincided with the stressors of final exams for students. Both studies used a placebo control, not fully described. Characteristics of these studies are detailed in Supplementary File S2.

One study was rated as “low quality” [22] according to SIGN-50 criteria for risk of bias whereas the other was rated as “acceptable” [21]. Criteria not sufficiently described included blinding, the randomization process and adequate concealment. It was not clear whether intention to treat analysis were carried out. In addition, although the manufacturer/product name was disclosed in these studies, there was no description of the method of authentication or any special testing of purity undertaken to ensure the supplement contained the materials as reported on the product.

#### 3.3.1. Outcomes

Illness logs and diaries were used to track cold and flu symptoms as reported by participants. As a secondary outcome in the study, Nantz reported 26 versus 28 in the AGE versus placebo groups got ill (RR 0.93 (95% CI, 0.63, 1.36; *p* = 0.71) [22] (Figure 1). Josling did not report on the number of persons experiencing a cold but rather the number of episodes throughout the trial, with 24 episodes in the garlic arm vs. 65 in placebo arm (*p* < 0.001) [21]. For Nantz the total number of symptoms reported during the study was also statistically significant in favor of garlic supplementation (584 total symptoms in garlic arm vs. 737 in placebo; *p* < 0.001). In contrast, the average number of symptoms per illness incident was not statistically significant (*p* = 0.536) [22].

Josling reported that the average duration of days with symptoms was statistically significant: the average number of days ill when taking garlic was 1.52 days vs. 5.01 days for placebo (*p* < 0.001) [21]. No standard deviations were provided in the report. Although the number of days having symptoms was not statistically significant in the Nantz study (*p* = 0.132), the number of work days missed was significant in favor of garlic (garlic 8 days vs. placebo 19 days; *p* = 0.035) [22].

Nantz also reported on other outcomes related to biomarkers and whether there were incidences of decreases in activity [22] Supplementary File S2.

#### 3.3.2. Adverse Events

Adverse events reported by Josling et al. [21] included rash and odor. There was no difference in the risk of experiencing an adverse event in this study (RD 0.04 (95% CI, −0.03, 0.11; *p* = 0.243) (Figure 4). Adverse events were not reported on in the study by Nantz et al. [22]. Natural Medicines reports that generally, garlic is well-tolerated with the most common adverse effects being “abdominal pain, body odor, flatulence, malodorous breath and nausea” and that serious adverse effects are rare but that “some case reports have raised concerns about an increased risk of bleeding”. According to Natural Medicines, “garlic is often standardized to allicin content with concentrations in most formulations ranging from 1.1% to 1.3%” and “most often used in doses of 2400 mg daily for 12 months” in adults. There is no known tolerable upper limit for garlic. Approximately 42 CAERS adverse events have been reported from 2014 to 2020. Supplementary File S2 details adverse events on record and highlights six published case reports.

#### 3.3.3. Other Studies

An additional study that appeared to meet the eligibility criteria but could not be obtained in the English language, is summarized only from its abstract available. [63] This study involved two stages, the first looking at the tolerance of allicor (600 mg/day) and its effects on acute respiratory diseases (ARD). The second stage investigated the effects of allicor (300 mg/day) on ARD morbidity during a five month trial in 42 children aged 10–12 years in comparison to 41 placebo treated children and 73 benzimidazole treated children. Allicor was not shown to induce gastrointestinal side effects and at the second stage of the study, allicor reduced ARD morbidity “1.7 fold compared to placebo and 2.4 fold vs. benzimidazole”.

#### 3.3.4. Quality of the Evidence

The two studies included in this review involving adults throughout the winter months show similar findings in that participants may experience less symptoms or episodes when taking garlic supplements. Methodological quality is a concern. In addition, the amount and formulation of the garlic differed in the studies and so inconsistency is of concern. It does not appear from this limited work that participants are at less risk of experiencing an illness. A similar finding among children taking allicor showed reduced “morbidity” compared to placebo. Overall, these studies are small in sample size and thus imprecision would be of concern. Further research would be required to confirm any of these findings.

### 3.4. Vitamin A

Vitamin A is a fat-soluble vitamin naturally present in many foods. The amount of vitamin A you need is dependent upon your age and sex. For adults, the recommended daily allowance is 900 μg retinol activity equivalents (RAE) for males and 700 μg RAE for females; and for children aged 4–8 years, 400 μg RAE is recommended (1 IU retinol = 0.3 μg RAE). Many multivitamin and mineral dietary supplements contain vitamin A either in the form of retinyl acetate or retinyl palmitate (preformed vitamin A), beta-carotene (provitamin A) or in some combination [64]. Although vitamin A deficiency is rare in the US, it is common in many developing countries where children are at high risk of diarrheal diseases and measles. Vitamin A is an ingredient identified on NBJ top 20 ingredients ranked by immunity sales in 2020 [7] and also a top ingredient identified as an “immune boosting” dietary supplement ingredient appearing in greater than 10% of webpage advertisements based on Google analytics during the 1st and 2nd wave of COVID [8]. We were unable to identify any single ingredient products advertised for immune health in our market analysis, however it is commonly listed as an ingredient in multivitamin products for immune health.

Studies on vitamin A are mostly among young children and infants in countries at high risk for vitamin A deficiency and susceptible to measles. Only two studies met the review’s eligibility criteria involving children with a mean age of four or greater and healthy at the time of the study. One study involved 1520 children less than 10 years of age from three ‘slums’ of Chandigarh, India where the prevalence of vitamin A deficiency is high (60% of the children had sub-clinical vitamin A deficiency status). Depending upon their age, they were given up to 2 mL (200,000 IU) vitamin A (type not disclosed) once every 4–6 months during a 15 month period [25]. The second study involved 445 preschool children (aged 3–6 years) in western China with serum retinal status of 1.15 ± 0.30 µmol/L, some of whom took 200,000 IU vitamin A (as retinol) once and were monitored over a six months period [24]. Characteristics of these studies are detailed in Supplementary File S2.

Both of these studies were rated as “low quality” according to SIGN-50 criteria for risk of bias. Criteria not adequately described included blinding and allocation concealment, whether intention to treat analysis was carried out, and the ways in which relevant outcomes were measured and reported. The products used in the study were not disclosed and there was insufficient information as to whether any testing was done to ensure it contained the given amounts reported as taken.

#### 3.4.1. Outcomes

Respiratory-related illness were secondary outcomes in these studies. Chowdhury showed that the incidence of diarrhea and measles was significantly reduced as well as the prevalence of vitamin A deficiency for the children receiving vitamin A. However, no statistical difference in the incidence of acute respiratory infections (number of episodes) was noted compared to the control group “RR 0.80 (95% CI, 0.61, 1.04); *p* = 0.09” [25]. Chen et al. reported that the incidence rate of respiratory-related illnesses and runny nose, cough and fever symptoms of the vitamin A group was significantly lower than the placebo group (*p* < 0.05), and results overall were enhanced in children taking a combination of vitamin A plus iron. Through analysis there was no difference between those taking vitamin A vs. placebo in the risk of getting at least a runny nose, cough or sore throat respiratory related illness (RR 0.95 (95% CI 0.65, 1.39; *p* = 0.78) [24] (Figure 1).

#### 3.4.2. Adverse Events

Adverse events were not described in these studies evaluated. Natural Medicines reports that generally, vitamin A is well-tolerated at doses below the tolerable upper intake level (UL) of 3000 μg in adults and 900 μg for children aged 4–8 years old. In very high doses vitamin A can cause pseudotumor cerebri, pain, liver toxicity, coma and even death, although these serious adverse effects are rare. Approximately 16 CAERS adverse events have been reported from 2014 to 2020. Supplementary File S2 details adverse events on record and eight published case reports.

#### 3.4.3. Quality of the Evidence

These studies are of low quality and report on secondary outcomes inconsistently which make interpretation challenging. With only these two trials available, imprecision would be a concern, and thus insufficient evidence for vitamin A as a single-ingredient dietary supplement for preventing respiratory infections in healthy individuals.

### 3.5. Vitamin C

Vitamin C is an essential vitamin that serves an important role in the functioning of the immune system and is needed for the body to develop and function properly. Dietary sources of vitamin C include various fruits and vegetables, most commonly oranges and orange juice. The recommended daily allowances for adults are 90 mg for males and 75 mg for females. Vitamin C can also be obtained from dietary supplements, commonly marketed with claims for “daily immune support”, and “antioxidant protection”. In 2020, vitamin C represented 18.2% of the cold, flu and immunity supplement sales in 2020, which reached close to $6 billion [1,2]. It was also found as the most common “immune boosting” strategy portrayed on the internet during the COVID-19 pandemic according to a content analysis of Google search results [8].

Three studies met the eligibility criteria and were included in the current review [26,27,28]. It is important to point out that a significant drop in the number of studies on vitamin C has occurred since the 1970′s. Early research on vitamin C not captured in this review, suggested that vitamin C had beneficial effects on the common cold (at >2 g/day). [65] Today, the tolerable upper intake levels for vitamin C is 2 g/day for ages 19 and above. Hemilä [66] explained the reason for the reduced interest in vitamin C research being the introduction of antibiotics in the mid-20th century, the belief that vitamin C is “ineffective” has been widely spread among general practitioners since three seminal papers published findings of no benefit of vitamin C for the common cold in the 70’s. Thus, the use of vitamin C for preventing and treating colds fell into the category of alternative medicine, and hence may amplify “prejudices against vitamin C”.

All three studies included in this review were conducted during the peak of cold season, throughout the winter months, up to 90 days in duration where participants were asked to take 1g/day of Ester-C ascorbate, [28] ascorbic acid prepared by Teva Pharmaceutical Industries [26] or a product not fully described but supplied as Twin lab C Caps [27]. Van Straten et al., 2002, included 168 healthy participants from the UK, the majority being female (84%) with a mean age of 48 years [28]. Constantini et al., 2011, included 39 competitive young swimmers aged 12–17 years from Israel, who trained at least 15 h/week (43% female) [26] and Johnston et al., 2014, included 30 healthy men aged 18–35 with plasma vitamin C concentrations less than 45 μmol/L [27]. All studies used placebo as a comparison. Characteristics of these studies are detailed in Supplementary File S2.

Studies were rated as “acceptable” and “high quality” according to SIGN-50 risk of bias. Only one study [27] reported on the participant’s vitamin C status at baseline, and assessed diet quality by using a food frequency questionnaire throughout the trial. No content analysis or purity testing was described in studies for the dietary supplement used.

#### 3.5.1. Outcomes

The number of persons experiencing at least one infection was reported in two studies by using the Wisconsin Upper Respiratory Symptom Survey 21 or through daily diaries. Up to 45% less risk was associated with taking vitamin C prophylactically (RR 0.55 (95% CI, 0.31, 0.99; *p* = 0.048), in men with plasma vitamin C concentrations less than 45 μmol/L, however the number of colds reported collectively did not reach statistical significance between groups [27]. In contrast, in healthy participants from the UK (the majority being female) did not appear to differ in the risk (RR 1.03 (95% CI, 0.72, 1.48; *p* = 0.88) (Figure 1), but a statistically significant difference was noted in the number of cold episodes (vitamin C, 37; control, 50; *p* < 0.05) [28]. The study in competitive swimmers from Israel did not show a significant difference in the number of episodes per person or the total number of episodes of respiratory infections experienced [26].

The severity of symptoms was assessed in the Constantine and Johnston studies [26,27]. Both showed a reduction in severity in favor of vitamin C, but it did not reach statistical significance. Although wide confidence intervals, the effect for men with marginal vitamin C status was moderate (SMD −0.60 (95% CI, −1.37, 0.16; *p* = 0.11) [27]. In addition, whereas the effect was insignificant and small for competitive swimmers overall (SMD −0.24), when looking at a sub-group of only males, this difference became significant in favor of vitamin C in lowering the severity of symptoms with a medium effect size (SMD −0.67 (95% CI, −1.24, −0.09; *p* = 0.02) [26] (Figure 2).

The duration of colds was reduced in all three trials, some of which were not statistically different than those in the placebo arms due to wide confidence intervals. However, when looking at males only these results were significant in favor of vitamin C, with up to 4.9 days less duration of symptoms when taking vitamin C compared to placebo [26] (Figure 3). Johnston et al. also described other measures of daily living and physical activity, reported in Supplementary File S2.

#### 3.5.2. Adverse Events

One study reported on adverse events experienced throughout the trial including indigestion and heartburn [28]. There was no difference in the risk of experiencing an adverse event taking vitamin C or placebo (RD −0.02 (95% CI, −0.12, 0.07); *p* = 0.63 (Figure 4). Natural Medicines reports that generally, vitamin C is well-tolerated and the most common adverse effects include abdominal cramps, heartburn, headache, diarrhea, nausea and vomiting; these effects are more likely to occur at doses above the tolerable upper intake level of 2 g/day. Approximately 186 CAERS adverse events have been reported from 2014 to 2020. Supplementary File S2 details adverse events on record and five published case reports.

#### 3.5.3. Quality of the Evidence

The three studies included in this review were of adequate to high quality overall. They were consistent in that 1 g/day was used in each study for a duration up to 90 days throughout the winter months and all showed some benefit in taking vitamin C prophylactically. Participants involved however in each study were unique and perhaps not directly related to each other. Results were consistent in showing that males with marginal vitamin C status or who are subject to intense exercise, are likely to experience fewer days with symptoms if taking vitamin C prophylactically, perhaps more so than the general population. No studies meeting eligibility criteria were identified in young children or under other stressful situations. Given the reported drop in research in this area and that these studies are small and unique, earlier work done on vitamin C is important to understand the magnitude of the research [65,66,67,68].

### 3.6. Vitamin D

Vitamin D is an essential vitamin important in maintaining bone and overall health. It can be found in foods such as fish, eggs and fortified milk. Synthesis of vitamin D can be promoted by exposing the skin to sunlight. The dietary recommended allowances for vitamin D for 19–50 years of age is 15 μg (600 IU). Vitamin D insufficiency is common in the US, especially among older persons or those who might not get enough exposure to sunlight. According to the IOM guidelines, Serum 25-Hydroxyvitamin D concentrations <30 nmol/L are associated with vitamin D deficiency, 30 to <50 is generally considered inadequate for bone and overall health in healthy individuals, ≥50 is generally considered adequate, and >125 has been linked to potential adverse effects [69]. Vitamin D dietary supplements are commonly used to prevent or treat such deficiency or in persons with weak or brittle bones. During the height of the COVID-19 pandemic, vitamin D was found as one of the top “immune boosting” strategies portrayed on the internet and the interest and use of vitamin D increased among individuals interested in immune-related nutrients [8,9]. It was also one of the top 20 ingredients identified by the NBJ 2020 report on immune health dietary supplements [7].

Eighteen studies covering 20 publications were included in this review. The majority of studies were conducted outside the US and included the winter months. One study involved older adults where approximately 25% had baseline levels <50 nmol/L [29]; seven studies involved adults with mean baseline levels ranging from 58 to 75 nmol/L [30,36,39,42,43,45,46]; two studies involved personnel during military training (one reporting “25% sufficient” and the other a mean of 78 μmol/L at baseline) [34,38]; two involved taekwondo (mean baseline of 31 nmol/L) [37] or swimmer athletes with mean levels of 60 μmol/L [31]; two studies involved either high school [47] or university students [33] where the baseline levels were not disclosed; and four studies involved children with mean baseline levels ranging from 30 to 67.5 nmol/L [32,35,40,48]. Vitamin D was administered in monthly doses at 200,000 tapering to 100,000 IU per month (provided by Tishcon) for up to approximately 1.6 years in two studies [29,42]; in weekly doses at 10,000 to 20,000 IU/week in five studies (provided by Dartnells, Dekristol, EuroPharm, Ddrops, or Tishcom) [32,33,36,40,46] with the longest study lasting five years [36]; the remaining eleven studies provided vitamin D in daily doses ranging from 400 IU per day for children [35], Japanese adults [45] and men during military training [38] to up to 5000 IU per day in male athletes with mean levels of 31 μmol/L at baseline [37], for a study duration up to one year (provided by Solgar, DSM, Pure Encapsulations, Minisun, BioTech, CTS Chemical, and Zenyaku) [30,31,34,35,37,38,39,43,45,47,48]. A matching placebo control was sufficiently described in most of these studies. Characteristics of all studies are detailed in Supplementary File S2.

Methodological quality was rated as “acceptable” to “high quality” according to SIGN-50 risk of bias in all studies but one, which was rated as “low quality” [30]. Inadequate description of allocation concealment and intention-to-treat analyses were common reasons for downgrading study quality. The majority of studies disclosed the products by name and assessed baseline levels of vitamin D among participants.

#### 3.6.1. Outcomes

The number of participants experiencing infection was tracked using the Wisconsin Upper Respiratory Symptom Survey or through other self-report questionnaires. For New Zealand adults and older adults (N = 2) (some of which had inadequate levels below 50 μmol/L), monthly doses of 200,000 tapering to 100,000 for greater than one year, did not reduce the risk of experiencing an infection (RR 0.99, 1.01) [29,42]. In adults with mean baseline levels of 60+ nmol/L given weekly doses of 20,000 IU vitamin D throughout the winter season and up to five years total, (N = 2) there did not appear to be any treatment effect on infection risk for respiratory tract infections [36,46]. However, one study reported that when stratified by baseline levels below 40 (n = 4) “a 44% reduction in infection risk” to include other infections besides respiratory tract infections was noted [46]. University students taking 10,000 IU/week vitamin D for eight weeks during peak rates of rhinovirus (N = 1) showed reduced laboratory confirmed infections (RR 0.54 (95% CI, 0.34, 0.84; *p* = 0.007), but the risk of self-reported symptomatic upper respiratory infections only trended toward significance (RR 0.79 (95% CI, 0.61, 1.04; *p* = 0.09) [33]. Children in Vietnam (N = 1, mean levels of 67.5) who were taking 14,000 IU/week up to one year showed a reduced risk of experiencing influenza A, B or other respiratory illnesses, but not influenza alone (RR 0.85 (95% CI, 0.72, 1.00; *p* = 0.053) [40], whereas children in Mongolia (N = 1, 30 μmol/L) taking the same amount for three years did not experience a lower risk of acute respiratory infections (RR 1.00 (95% CI, 0.98, 1.02; *p* = 0.888) [32]. Other studies [35,47,48] (N = 3) in children or high school students outside the US and taking daily doses of 400–2000 IU vitamin D up to 5 months throughout winter, showed no difference in experiencing an influenza-like illness in general (RR 0.96, 1.26), but studies report that vitamin D may influence the risk of influenza A “in certain subgroups” [48] or “during the first month” [47] of taking vitamin D. Adolescent competitive swimmers in Israel taking 2000 IU/d (N = 1.60 μmol/L) did not experience a statistically less risk of suffering an infection compared to placebo [31]. (RR 0.86 (95% CI, 0.62, 1.18; *p* = 0.349) A study in young Finnish men during military training who took 400 IU/day vitamin D for six months (mean 78 nmol/L) showed preventative benefit in the risk of experiencing an infection (RR 0.76 (95% CI, 0.58, 1.00; *p* = 0.049) [38]. Other studies in adults inside and outside the US (vitamin D levels ranging from means of 58–82.5 μmol/L) who took vitamin D in doses of 400 IU to 2000 IU per day throughout the winter months and up to one year duration did not show any statistical difference in the risk of experiencing an infection [30,39,43,45] (RR ranging from 0.77 to 0.99, with confidence intervals crossing the line of no effect) (N = 4) (Figure 1).

Nine studies assessed the severity of symptoms throughout the trials, five of which data were available to quantify the SMD of the effect contributions of vitamin D vs. placebo. A large effect (SMD −3.66 (95% CI, −5.36, −1.96; *p* = 0.000) was shown for 17 male taekwondo athletes with inadequate levels of vitamin D throughout the winter months in reducing the severity of symptoms [37]. Small to no effect was shown in the remaining studies, in adults and adolescent swimmers (SMD ranging from −0.31 to 0.19) [31,33,39,45] (Figure 2). One study, for which data were not available for extraction, did report on British army recruits (25% sufficient) who took 1090 IU for four weeks during training followed by 460 IU for an additional eight weeks. They noted that vitamin D “reduced the severity of peak URTI symptoms by 15% [*p* < 0.05]” and that participants beginning supplementation with serum levels below 50 μmol/L “had 21% lower peak severity”. This study did combine vitamin D supplementation and simulated sunlight groups together when reporting results but stated “no difference between these groups when combined” [34].

Duration of symptoms for those that did experience an event was an outcome evaluated in eight studies. Three studies had data available for calculating a mean difference in days between those taking vitamin D vs. placebo, showing no difference in days with symptoms (MD days ranging from −0.80 to 0.10) with the largest non-significant effect in adolescent swimmers. [31,33,39] Harrison et al. reported “Compared with placebo, vitamin D supplementation reduced the days with URTI by 36% (*p* < 0.05); *p* < 0.05” [34] and Shimizu et al. reported the median days duration was 10 days vs. 13 days for 400 IU/d compared to placebo, although not statistically significant (*p* = 0.061) [45]. All other studies involving adults regardless of the amount or duration of taking vitamin D showed no change in duration of symptoms [42,43,46]. Other studies among children did not report on duration of symptoms.

Quality of life measures and biomarkers were reported in few studies, reported in Supplementary File S2.

#### 3.6.2. Adverse Events

Adverse events reported in studies evaluated were judged by study authors to be not related to the supplement, such as chest pain, common cold, stomachache and nausea. There was no risk difference in the number of participants experiencing any adverse event between the vitamin D supplement group and those taking placebo, as reported in two studies [39,45] (RD −0.01 (95% CI, −0.11, 0.09; *p* = 0.83) (Figure 4) Natural Medicines reports that generally, orally, vitamin D is well-tolerated and that serious adverse effects are rare but in “excessive doses can lead to vitamin D toxicity with symptoms of hypercalcemia and also sometimes azotemia and anemia”. According to the IOM guidelines, tolerable upper intake levels for vitamin D are 4000 IU per day for individuals nine years and older, although there is evidence of adverse events with doses as low as 2000 IU/day in some populations [70]. Approximately 637 CAERS adverse events have been reported from 2014 to 2020. Supplementary File S2 details adverse events on record and 14 published case reports.

#### 3.6.3. Quality of the Evidence

Methodological quality of studies is overall acceptable to high quality. Studies are consistent in assessing the effect of supplemental vitamin D throughout the winter months, but in various amounts, frequency and duration. The majority of studies reported mean baseline levels considered by the IOM guidelines as “adequate” overall. Sub-analysis to examine different levels for participants that may be deficient or insufficient for vitamin D, challenge power issues for sample size and imprecision becomes a concern in any results reported. It does not appear that monthly administration of vitamin D in adults is beneficial from two studies; few studies in children and adults exposed to various stressors taking either weekly or daily supplementation of vitamin D showed benefits associated with protecting immune health. These studies reporting benefits however are heterogeneous, which makes it challenging to know for which subgroups vitamin D may be beneficial.

### 3.7. Vitamin E

Vitamin E is an essential fat-soluble vitamin with antioxidant activities. Naturally occurring vitamin E exists in eight chemical forms, however alpha-tocopherol is the only form recognized to meet human requirements. It can be obtained naturally from many foods such as vegetable oils, cereal grains, animal fats and vegetables. Recommended intakes of vitamin E for ages 14+ is 15 mg per day. Vitamin E is also common in multivitamin dietary supplements and usually listed to provide substantially greater than the daily recommended intake [71]. In dietary supplements, vitamin E is often promoted for “immune health” and “antioxidant support”. Vitamin E was found as a frequent ingredient in the DSLD with claims for immune health.

The one study meeting the review’s eligibility criteria involved 652 noninstitutionalized individuals, aged 60 years and older (mean age, 73) from the Netherlands and considered “well-nourished” [49,50]. Participants were divided into four treatment groups: multivitamin and minerals, vitamin E alone, both combined or a placebo. Participants assigned to vitamin E took 2 capsules of 200 mg/dL alpha-tocopheryl acetate per day for up to 15 months, to include at least three winter months. Placebo was described as containing soybean oil. Characteristics of this study are described in Supplementary File S2.

The overall methodological quality of this study was rated as “acceptable” according to SIGN-50 risk of bias. Selective outcome reporting was a concern for which some data could not be used for quantitative analysis.

#### 3.7.1. Outcomes

The main outcomes were the incidence and severity of acute respiratory tract infections assessed through the use of a diary. Participants taking vitamin E were no more likely to experience an infection than those taking a placebo (RR 1.02 (95% CI, 0.88, 1.19; *p* = 0.76) (Figure 1). Among persons experiencing an infection, the individuals who received vitamin E had longer total illness duration (19 (9–37) vs. 14 (6–29) days [interquartile range reported], more symptoms (6 (3–8) vs. 4 (3–8), and a higher frequency of fever (36.7% vs. 25.2%) and restriction of activity than those not taking vitamin E [49,50].

This study also reported on restriction of activity and biomarkers reported in Supplementary File S2.

#### 3.7.2. Adverse Events

Adverse events beyond experiencing more severe illness were not reported on in this study. Natural Medicines reports that generally, vitamin E is well-tolerated. Serious adverse effects are rare but include bleeding hemorrhagic stroke and cardiovascular complications. The tolerable upper intake levels for vitamin E based on IOM guidelines for adults 19+ years is 1000 mg/day of vitamin E. Approximately 48 CAERS adverse event incidences have been reported from 2014 to 2020. Supplementary File S2 details adverse events on record and two published case reports.

### 3.8. Zinc

Zinc is an essential mineral that is found in foods such as red meat, poultry and fish. Recommended daily intake of zinc for adults 19+ is 11 mg for males and 8 mg for females. Zinc can also be obtained through dietary supplements in various forms as elemental zinc, zinc gluconate, zinc sulfate, zinc picolinate, and zinc acetate. It can also be found as lozenges and nasal sprays, sometimes advertised as dietary supplements, but not necessarily meeting the FDA definition for dietary supplements. In addition, zinc appears as an ingredient in some over the counter cold remedies/drugs. Zinc was found as one of the top “immune boosting” strategies portrayed on the internet during the COVID-19 pandemic [8]. In addition, it was one of the top 20 ingredients identified by the NBJ 2020 report on immune health dietary supplement ingredients [7]. Zinc either alone or present in combination products is popularly advertised to “support immune system function” and “immune health”. Homeopathic products often advertise zinc as a “pre-cold remedy”.

Six studies met eligibility criteria for review since 2001. Lozenges and nasal sprays were not considered as dietary supplement products and therefore not eligible for this review. 

Four studies (n = 1355 participants) included school aged children up to the age of 15 years, living outside of the US who took various forms of zinc for up to seven months, to include the winter months. Children took between 10–20 mg/day of zinc in the forms: zinc bisglycinate—Qualimed, Thailand [53], elemental zinc [55], and zinc sulphate supplied by Berko Ilac Company, Turkey [51] or a Peruvian pharmaceutical Lab (Instituto Quimioterapico, Peru) [54]. According to Kurugol et al. [51] if children experienced symptoms, they were given 30 mg/day for up to 10 days rather than 15 mg/day during the prevention stage. One study involved 40 male and female US Air Force cadets during the cold and flu season who were exposed to a number of stressful situations during their training; participants took 15 mg zinc supplied through a “FDA licensed clinical specialty pharmacy (FL, USA)” for seven months duration [56]. Finally, one study involved 50 healthy elderly adults aged 55–87 years, some of whom were zinc-deficient and stressed oxidatively more so compared to younger adults. Subjects took 45 mg/day elemental zinc for 12 months supplied by Labcatal Laboratories, Paris, France [52]. A matching control, considered as placebo, was sufficiently described in most of these studies. Characteristics of all studies are detailed in Supplementary File S2.

The methodological quality was rated as low quality for three studies [52,55,56] and acceptable [53,54] and high quality [51] in the other three studies, according to SIGN-50 risk of bias. Methodological concerns included the randomization process not being adequately described, allocation concealment, blinding and whether intention-to-treat was performed in studies. Not all studies reported on the zinc levels of participants at baseline and whether tests were done to confirm the supplement contained the amount of zinc reported on the Supplement Facts label.

#### 3.8.1. Outcomes

The incidence of experiencing symptoms was determined through self-report. One study in children provided data to suggest significantly less risk of developing the common cold when taking zinc prophylactically for seven months RR 0.78; (95% CI, 0.67, 0.92; *p* = 0.003) [51]. Another study showed no difference between children taking zinc compared to placebo in those experiencing at least one symptom [53] (Figure 1). However, this study did show less risk of experiencing two or more symptoms throughout the three months (RR 0.46 (95% CI, 0.19, 1.12; *p* = 0.087) [53]. The other two studies in children did not report on the number of participants experiencing symptoms but did report on the number of episodes experienced. Vakili et al. [55] in 2009 reported that the average common cold episodes per child during the study was 1.37 episodes among those taking zinc compared to 3.15 episodes among placebo (*p* < 0.001). In an environment where malaria is prevalent, zinc supplementation did not impact the incidence of respiratory infections [54]. A study involving 40 cadets exposed to stressors during the winter months did not show a difference between groups in terms of physician-diagnosed cases of infection, however self-reported symptoms through a weekly survey showed that participants taking zinc experienced more symptom-free episodes compared to the placebo group (*p* = 0.01) [56]. Significantly less risk of experiencing an infection was shown in one study evaluating the effect of zinc in seniors over a 12 month period (RR 0.33 (95% CI, 0.17, 0.63; *p* = 0.001) [52] (Figure 1).

The duration of any episodes was significantly reduced as reported in two studies involving children [51,53]. The mean difference in cold symptoms in the zinc group compared to the placebo was MD −0.60 (95% CI, −0.78, −0.42; *p* = 0.0001) in one study, [51] where the other reported that the median duration of at least 2 symptoms (in days, (IQR) for those taking zinc was 0.0 (0–1.0) whereas the placebo group was 1.0 (0–5.3) *p* < 0.01 [53] (Figure 3). Vakilli et al. reported on days missing school, which was significantly reduced for those taking zinc (MD −0.80 (95% CI, −1.21, −0.39; *p* < 0.0001) [55]. The studies in cadets and seniors did not evaluate duration of symptoms (Supplementary File S2).

Biomarkers for zinc plasma concentrations are reported in Supplementary File S2.

#### 3.8.2. Adverse Events

Adverse effects reported in studies included mild gastrointestinal discomfort and bad taste. There was no sign of a significant risk difference in those taking zinc compared to placebo as reported in one study [51] (RD 0.02 (95% CI, −0.11, 0.15; *p* = 0.77) (Figure 4). Natural Medicines reports that generally, orally, zinc is well-tolerated in doses below the tolerable upper intake level (40 mg daily for adults). Most common adverse effects include abdominal cramps, diarrhea, metallic taste, nausea and vomiting, which are commonly dose-related. There have been approximately 249 CAERS adverse events reported from 2014 to 2020. Supplementary File S2 details adverse events on record and three published case reports.

#### 3.8.3. Quality of the Evidence

There is a concern of risk of bias across half of the studies included. In addition, the outcomes reported varied in the way in which statistics were presented, which makes the interpretation of consistent results challenging. From this limited evidence, there may be benefit for children outside the US taking zinc up to 20 mg/day prophylactically during the winter months to reduce the incidence and duration of symptoms. No studies on children from the US were included in this review. In addition, there was only one study in US cadets experiencing various stressors and one study among seniors, both showed benefit in taking zinc dietary supplements. Further research in these populations is needed to confirm any findings.

## 4. Discussion

The purpose of this review was to critically evaluate the evidence for select ingredients frequently advertised and contained in immune boosting dietary supplement products, for the otherwise healthy consumer looking to maintain health and resist, or bounce back quicker after getting sick upon exposure to viral or respiratory infections as well as other life stressors and challenges encountered through daily living. Of the 27 ingredients discovered and selected from the market analysis, as frequently contained in dietary supplements with claims for immune boosting effects, the authors identified only eight dietary supplement ingredients for which there was peer-reviewed literature meeting the eligibility criterion for systematic review. Table 2 details the summary of the evidence for echinacea, elderberry, garlic, vitamin A, vitamin C, vitamin D, vitamin E and zinc, in an attempt to answer the research questions posed for review. Whereas some studies may point to evidence for benefit or perhaps even no benefit, specific gaps preclude the authors from making firm statements in regard to the overall evidence-base for these products and ingredients, and in answering the research questions overall that require diverse researchers and scientific collaborations, for forging the path forward in understanding whether the science would match the claims made for dietary supplements that protect immune health. As part of this project, an expert panel was assembled and convened to review this evidence and discuss research considerations and gaps. Using a modified Delphi process, the expert panel established a set of priorities and recommendations for future research based on the state of the science and in collaboration with key stakeholders, reported elsewhere (currently in draft).

The authors acknowledge the challenge in defining some of the claims made in the marketplace for dietary supplements. For example, we do not really know what “boosting” the immune system means in scientific terminology. The authors performed a scoping review at the outset of this project in an attempt to understand what systematic reviews exists in the literature base around the term, “immune” (Supplementary File S1). Most of these reviews focus on patients with inflammatory conditions, such as atopic dermatitis, periodontal diseases, ulcerative colitis, etc., or other disease states or conditions such as cancer and heart disease. Some look at “prevention and treatment” of respiratory conditions. Previous reviews suggest that echinacea might have a preventative benefit for upper respiratory tract infections, including the common cold, but whether any reported effect is clinically meaningful is debatable [72,73]. Elderberry may reduce the duration and severity of symptoms in individuals taking elderberry as a treatment for influenza-like symptoms. Research studying elderberry prophylactically is pointed out as requiring more information in order to make any conclusions [74,75]. Only one review (involving a single study) evaluating the evidence on garlic for the prevention or treatment of the common cold was identified [76]. Reviews on vitamin A suggest that the effect on respiratory infections could differ by age and circumstance; and that excessive amounts could actually increase the risk of infection in children who are already healthy, without vitamin A deficiency or malnourishment [67,77,78,79]. Reviews on vitamin C suggest that preventative benefits may be dependent on gender (males), environmental conditions and certain stressors an individual may be exposed to; this will require future attention [66,67,68]. A number of systematic reviews have looked at vitamin D, especially since the COVID-19 pandemic [67,78,80,81,82,83]. Sub-group analyses have shown that vitamin D may be beneficial as a daily dose over a short-term, but for which individuals with which baseline levels or involving what stressors is not yet fully understood. Previous reviews including vitamin E do not appear to show any benefit for the prevention or treatment of respiratory infections [67,78,84,85]. Reviews on zinc (including lozenges and nasal sprays in addition to capsules and tablets) suggest a reduction in the days with symptoms with zinc but less known about the preventative benefits [67,78,86,87]. Limitations noted in reviews and consistent with our review include methodological concerns and clinical heterogeneity in many different preparations tested and among different types of individuals.

This review set out to look at otherwise healthy individuals, with and without stressors characterized in the study. Stressors discovered through this review involved being exposed to stressful air travel, intense exercise, academic stress, exposure to winter months, environmental stressors such as poor living environments or where deficiency in certain nutrients is prevalent, and subjects inoculated with a virus, with wintertime being the most commonly researched stressor among the included studies. Other stressors were not characterized and studies were overall small in sample size and involved varying products, amounts, and duration of use. These limitations compromised our ability to pool for meta-analysis. In addition, the authors evaluated single ingredient products to understand the evidence for specific ingredients. Most products on the market consist of several ingredients in combination. An evaluation of multi-ingredient products is challenging due to the various ingredient combinations unique to each product on the market. An investigation into single ingredient product is the first step to understanding the evidence-base.

### Applicability to Practice

Thousands of dietary supplement products are on the market with immune claims on product labels and cited through various websites selling dietary supplements. Consumers are likely overwhelmed and have little information to rely on for selecting a supplement to safely support their diet, and to enhance their capacity to resist, adapt to, recover, or grow from a stressor or challenge. This evaluation serves as a foundation of the evidence for these frequent ingredients contained in immune “boosting” dietary supplements and a knowledge vehicle to generate future research agendas based on the current gaps and in the focus of resilience for the otherwise healthy individual. For the consumer today, existing resources are available to assist consumers in choosing supplements safely, such as through Operation Supplement Safety (OPSS.org) and the Office of Dietary Supplements at the NIH. It is important for the consumer to know that dietary supplements are not regulated in the same way as conventional drugs, and industry has many freedoms in the ways in which they market supplements. Some safety tips are to choose supplements that: (1) contain third-party certification seals such as BSCG, NSF Informed Sport and USP; (2) do not have multiple ingredients listed on the Supplement Facts label, especially ones that you cannot even pronounce; (3) have quick-fix statements that seem too good to be true; and (4) are free of proprietary blends, matrices or complexes [88,89].

## 5. Conclusions

Claims made for boosting the immune system on dietary supplement products and the ingredients contained in those products do not appear to have overall, strong scientific evidence as of yet, for the otherwise healthy consumer looking to maintain health, resist getting sick, or recover quickly. There is little evidence to suggest as to that certain ingredients if taken prior to getting sick, will reduce the severity or duration of any acute respiratory infection, versus not taking a dietary supplement prophylactically or as compared to placebo. As we move toward a vision of health promotion and resilience rather than a sole focus on disease prevention and treatment, further work in this area of dietary supplements is of utmost importance.

## Figures and Tables

**Table 1:** Eligibility criteria for systematic review.

PICOTS	Eligibility Criteria
Populations	Otherwise healthy individuals who may or may not be experiencing a stressor, such as intense exercise, psychological stress, fatigue, air travel, sub-optimal environmental conditions, seasonal stressors such as winter time, school environments, a history of recurrent infections but not sick at the time, and exposure to a vaccination. Stressors were not pre-defined, but captured as they emerged naturally throughout the screen phase and tagged as discovered. To be consistent with the dietary intake recommendations, the authors chose to include children four years or older, or studies that included children with at least a mean age of four years, adults 18 years and older, and seniors, defined as 70 years and older but only including free living, non-institutionalized persons. Individuals with chronic conditions or taking medications were not considered eligible.
Dietary supplement ingredients	Dietary supplement ingredients selected from the market driven approach and delivered as a single ingredient dietary supplement product. For eligibility, the authors followed the Food and Drug Administration definition for dietary supplements. Nasal sprays and lozenges were not considered dietary supplements under this review. Studies including dietary supplements as a treatment were not eligible, but taken prophylactically, to preserve, protect or recover from, taken prior to any sign of infection or symptom occurring. If the participants included in a study were sick at the time of enrollment, those studies were not eligible. In addition, if the participants were taking other medications concurrently with the dietary supplement, then those studies were not eligible (e.g., asthma medications, other prescription drugs). Dose/amount of ingredient had to be reported in the publication to be included.
Control/ comparators	Placebo, waitlist, usual care.
Outcomes	Primary: Incidence of infection, severity, duration of symptoms, adverse events. Secondary: performance, general well-being, mood, quality of life, fatigue, sleep quality, psychological stress, dietary habits/changes, as well as biomarkers reported in conjunction with primary outcomes (e.g., C-reactive protein [CRP], Interleukins [ILs], Interferon [IFN], Tumor necrosis factor [TNF], etc.).
Timing	Not restricted.
Study designs	Efficacy: Randomized controlled trials; Additional information on safety: case reports, adverse event reporting databases and Natural Medicines adverse effects monographs.

**Table 2:** Summary of the Evidence.

No. Studies (Participants, Stressors)	Risk of Getting an Infection	Severity of Symptoms	Duration of Symptoms	Adverse Events	Research Considerations and Research Gaps
Various Echinacea products in varying amounts and duration of use, up to 4 months taken prophylactically					
5 adult studies (n = 1505) Inoculation with virus (N = 2) [17,19], exposure to winter months (N = 2) [14,16], stressful air travel (N = 1) [18]	3 studies consistently show less risk (RR 0.71 to RR 0.82; up to 29%) when taking Echinacea—none reach statistical significance on their own *2 studies did not report per person statistics*	2 studies show very small to no effect on the severity of symptoms—none reached statistical significance (SMD −0.29, 0.03) 2 studies describe less symptom severity for Echinacea group—borderline or no statistical significance	2 studies describe reduction in duration of symptoms but not statistically significant	RD 0.02 (95% CI, −0.01, 0.05; *p* = 0.24, N = 5)	Studies show consistency in results for outcomes reported across studies and involving healthy adults exposed to various stressors. Echinacea products “appear” safe for short-term use. Two of the largest trials showed benefit for both adults and children taking EchinaForce over a four month period throughout winter months. Studies are relatively small in sample size, likely lacking sufficient power to detect differences between groups It is unknown if any benefit detected from studies could be clinically meaningful Studies are ultimately not combinable for quantitative analysis to understand the effect of specific formulations, amounts and duration for use Duration of symptoms when using Echinacea prophylactically or for treatment has not been well researched Safety of long-term use unknown No studies reporting outcomes related to well-being beyond these primary outcomes assessed (e.g., sleep, diet, psychological stress) Standardization, formulation, method of authentication and chemical fingerprint largely unknown Lack of understanding of comparison between different extracts of Echinacea products (*E. purpurea, E. angustifolia*), or combination of both extracts Lack of understanding of appropriate amounts per day for different persons or what might be considered a tolerable upper limit Lack of understanding regarding what types of stressors for which Echinacea might be useful, as well as when and for who
1 child study (n = 203) [15] During winter time season	RR 0.70 (95% CI, 0.52, 0.95; *p* = 0.023); up to 30% less risk when taking Echinacea	SMD −0.49 (95% CI, −0.77, −0.21; *p* = 0.001)	MD −1.4 days (95% CI, −2.39, −0.41; *p* = 0.008)	RD 0.04 (95% CI, −0.10, 0.17; *p* = 0.61, N = 1)	
Elderberry (Sambucus nigra L., Haschberg variety; BerryPharma Brand); 600 mg/day for 10 days before travel and 900 mg/day for 5 days (e.g., 1 day before leaving home until 4–5 days after arriving at the destination); 15–16 days total					
1 adult study (n = 312) [20] During stressful air travel	RR 0.69 (95% CI, 0.34, 1.39; *p* = 0.298); up to 31% less risk when taking elderberry	*the symptom score in the placebo group over these days was 583, whereas in the elderberry group it was 247 (p = 0.02)* *decreased the symptom load (mean 21* vs. *34) SD not provided*	Number of episode days: T = 57; C = 117 (*p* = 0.05) *On average, a 2 day shorter duration of the cold (4.75* vs. *6.88 d)*	RD −0.01 (95% CI, −0.03, 0.02; *p* = 0.632, N = 1)	*One study on the prophylactic use of Elderberry, showing decrease in severity and duration of symptoms.* *Elderberry “appears” safe if properly prepared.* Lack of studies available on the prophylactic use of elderberry This study looked at physical and mental components of health (SF-12) during travel. Physical health declined in placebo group but remained stable in intervention group; intervention group trended toward higher mental health than placebo group. There is no known tolerable upper limit for elderberry
Various Garlic products in varying amounts taken throughout the cold and flu season, up to 90 days					
2 adult studies (n = 266) [21,22] Throughout cold and flu season	1 study showed the incidence of cold and flu was not statistically significant (RR 0.93 (95% CI, 0.63, 1.36; *p* = 0.71) *Second study did not report per person statistics*	Total number of episodes and total number of symptoms statistically significant in trials (*p* < 0.001)	Duration of days shown to be significant in one study *(1.52 days in garlic* vs. *5.01 days placebo (p < 0.001)* Number of days in other trial not significant but the *number of work days missed was (8 days* vs. *19 placebo days (p < 0.035)*	RD 0.04 (95% CI, −0.03, 0.11; *p* = 0.243, N = 1)	Two studies show that adult participants taking garlic supplements throughout the cold and flu season may experience less episodes and symptoms overall. Lack of high quality studies available on the prophylactic use of garlic supplements for immune health The two trials use very different amounts **(180 mg vs. 2.56 g)** and formulations of garlic supplements Safety not fully understood for what amount and duration Data presented in studies did not provide sufficient information for quantitative analysis on most outcomes One study looked at decrease in activity and reported that incidence and total days of decrease in activity was statistically significant in favor of garlic Garlic consumption may enhance immune cell function given the data available on biomarkers There is no known tolerable upper limit for garlic
Vitamin A (200,000 IU) taken once every four to six months, for a duration up to 15 months					
2 child studies (n = 1719) Children from India [25] and China [24] that are less than 10 years old	Respiratory related illnesses RR 0.95 (95% CI 0.65, 1.39; *p* = 0.78), N = 1 study *Incidence child/year for ARI* *T = 0.288; C = 0.361,* N = 1 study	Number of respiratory related events from one study VAS 262 vs. 284 in placebo group Number of episodes in other study *88 VAS* vs. *147 in placebo group*	Average days experiencing respiratory related events, *VAS 5.5* vs. *8.0 placebo group;* SD not provided, N = 1 study	Not reported	Two studies conducted in countries with high prevalence of vitamin A deficiency, in children less than 10 years of age. Studies of low quality Studies report statistics in varying ways and selective outcomes reporting of concern Both studies report on these outcomes as secondary outcomes only No research in healthy adults meeting eligibility criteria Risk is associated with very high doses Vitamin A in multi-ingredient dietary supplements at low amounts appears safe
Vitamin C 1000 mg/day taken prophylactically up to 90 days in duration throughout the winter months					
3 studies (n = 237) adolescent competitive swimmers (N = 1) [26], adults in the UK (N = 1) [28], males with adequate-low plasma C levels (N = 1) [27]	1 study in males showed up to 45% less risk of developing symptoms RR 0.55 (95% CI, 0.31, 0.99; *p* = 0.048) 1 study involving mostly females showed no difference RR 1.03 (95% CI, 0.72, 1.48; *p* = 0.88) in risk but less number of cold episodes overall. 1 study involving competitive adolescent swimmers no difference detected	Severity of symptoms reduced in two studies but not statistically significant In males alone the effect size was SMD > 0.60 (considered medium effect) in two studies, one reaching statistical significance for competitive swimmers	Duration of illness reduced in all three trials (MD > 1 day), not all statistically significant In males alone, up to 4.9 days less duration, reaching statistical significance in two trials	RD −0.02 (95% CI, −0.12, 0.07); *p* = 0.63, N = 1)	Three studies show some benefit of taking 1000 mg vitamin C per day throughout the winter months. Study involving males with adequate to low vitamin C levels show less risk of getting sick if taking vitamin C prophylactically during the winter months. Males and perhaps those exposed to intense exercise may benefit from less severe symptoms and reduced duration of illness. Vitamin C appears safe below the tolerable upper limit of 2 g/day. Only one study looked at vitamin C levels in the body and tracked diet throughout the trial The amount and timing for vitamin C to take prophylactically or therapeutically is not fully understood The size of any effect of vitamin C may be dependent upon specific participant characteristics or stressors
Various Vitamin D products in varying amounts, timing and duration of use, up to five years taken prophylactically					
2 studies in adults [42] and seniors [29] (n = 5432, some with inadequate levels) 200,000 tapering to 100,000/month (3300 IU/d) Tishcon, up to 1.6 years	Two studies showed no significant reduction in risk of infection (RR 0.99, 1.01) and *result remained* *unchanged when the analysis included* *winter season or baseline 25-OHD levels*	One study reported *No. episodes: T = 593; C = 611 and Severity per episode, median (IQR); T = 171 (86–295); C = 183 (97–316); p = 0.48*	One study reported *Median (25th percentile, d) duration of symptoms per episode* *T = 12 (8), n = 366; C = 12 (7); n = 365; p = 0.76 and No. days missed work per episode* *T = 0.76 (1.25); C = 0.76 (1.26) p = 0.82*	Both reported vitamin D did not affect any reported adverse events	Two studies in adults and seniors regardless of vitamin D status taking monthly doses of vitamin D up to 1.6 years did not reduce the risk of infection. Weekly doses of vitamin D for university students during the winter showed some reduced risk of infection. Other 2 studies in adults with adequate levels did not. Children taking weekly doses in Vietnam showed reduced risk of infections but not for influenza alone while children in Mongolia with inadequate levels showed no effect for risk of infection over a longer time period. 4 studies in children taking daily vitamin D during the winter months did not show significantly reduced risk, severity or duration of illness. 3 studies involving either male during military training or taekwondo training show either less risk of infection or reduced symptom severity when taking daily vitamin D. Four studies in adults with mean adequate levels, taking daily vitamin D during the winter months showed no significant benefit in risk, severity or duration. Vitamin D appears safe when taken below the UL Certain stressors or sub-populations may be insufficiently studied to understand the precision of results beyond the “winter months” stressors It is challenging to know whether those deficient benefit from vitamin D for respiratory infections when a mean serum level is reported and sub-analysis is done for different levels that is not adequately powered to detect meaningful changes.
3 adult studies (one involving university students) [33] (n = 1145) 10,000–20,000 IU/week (8 weeks, 16 weeks, 5 years) to include winter seasons [36,46]	Two studies showed no reduced risk *Infection hazard (HR) for all URTI; T = 1.11 (0.75, 1.65); C = 1.00 (reference) p = 0.62, N = 1;* RR 1.22 (95% CI, 1.00, 1.5; *p* = 0.055, N = 1 1 study with university students trended toward self-reported reduced risk (RR 0.79 (95% CI, 0.61, 1.04; *p* = 0.09)	One study reported *No. episodes of RTI* *T = 36; C = 32 and Average daily total infection symptom severity: T =* −*1.08 (*−*3.00, 0.85); C = 5.28 (3.73, 6.82) p = 0.27* University students *Complete case URTI episodes; T = 70; C = 80; p = 0.09; but no effect on symptom severity* SMD 0.16 (95% CI, −0.22, 0.54; *p* = 0.406)	One study reported *Infection duration (HR) for URTI T = 0.51 (*−*1.80, 2.81); C = 4.87 (3.29, 6.45); p = 0.67* University students MD −0.20 (95% CI, −0.76, 0.36; *p* = 0.480)	Similar in both groups, not reported or none occurred	
2 child studies (n = 20,252) 14,000 IU/week up to 3 years [32,40]	Children in Vietnam (RR 0.85 (95% CI, 0.72, 1.00; *p* = 0.053, N = 1) Children in Mongolia with mean inadequate levels (RR 1.00 (95% CI, 0.98, 1.02; *p* = 0.888, N = 1)			None related to vitamin D	
4 child studies (n = 862) 400–2000 IU/day up to 5 months During winter months (one study involving adolescent competitive swimmers) [31,35,47,48]	Two studies show no reduction in risk RR 0.96, 1.26 One study reported increased risk in both groups but no difference bt groups *(all p > 0.57)* Competitive swimmers *no significant less risk* (RR 0.86 (95% CI, 0.62, 1.18; *p* = 0.349)	Competitive swimmers no difference in severity of symptoms SMD 0.19 (95% CI, −0.65, 1.03; *p* = 0.641)	One study reported *Number of students absent T = 68/148; C = 38/99 and Mean absences, d; T = 1.7 (2.5); C = 1.1 (1.9); p = 0.14* Competitive swimmers *no significant difference in days with illness* MD −0.80 (95% CI, −2.41, 0.81; *p* = 0.329)	Studies either did not report on AE or reported none occurred	
1 adult study in Finnish men during military training (400 IU/day; mean adequate levels), [38] 1 study in British army recruits (25% sufficient 1090 (4 weeks), 460 IU (8 weeks)) [34] and one study in So. Korean men during taekwondo training (5000 IU/day; mean inadequate levels) [37] up to 6 months (n = 440)	Finnish men during military training show less risk of infection (RR 0.76 (95% CI, 0.58, 1.00; *p* = 0.049)	Male TKD athletes Less symptom severity SMD −3.66 (95% CI, −5.36, −1.96; *p* = 0.000) British recruits: *vitamin D supplementation reduced the severity of peak URTI* *symptoms by 15%; p < 0.05; N = 1*	*Mean days absent from duty; T = 2.2 (3.2); C = 3.0 (4.0); p = 0.096, N = 1* British recruits: *vitamin D supplementation reduced the days with URTI by 36% (p < 0.05); N = 1*	Not reported	
4 adult studies (n = 1279) 400–2000 IU/day up to 1 year with mean adequate levels [30,39,43,45] Throughout winter months	No significant difference in four studies RR ranging from 0.77 to 0.99	Severity of symptoms ranged from SMD −0.31 to −0.18, N = 2 considered small effect, not significant *Mean episodes per person-month reported in another study not significant*	Three studies reported duration in different ways, none significant *days of illness per person-month T = 1.52 (2.98); C = 1.56 (2.87); adjusted RR 1.03 (0.81, 1.31), N = 1* MD 0.10 (95% CI, −1.51, 1.71; P = 0.902), N = 1 *Median 10 days* vs. *13 days p = 0.061, N = 1*	RD −0.01 (95% CI, −0.11, 0.09; *p* = 0.83) N = 2	
Vitamin E 2 X 200 mg/dL of alpha-tocopheryl acetate per day for up to 15 months to include the winter months					
1 study among seniors [49] (n = 652) noninstitutionalized individuals aged 60 years or older from Netherlands with overall healthy micronutrient status	A greater risk of experiencing an acute respiratory tract infection RR 1.02 (95% CI, 0.88, 1.19; *p* = 0.76, N = 1 study)	*Number of symptoms, median (IQR) T = 5 (3–8); C = 4 (3–8)* *Number of episodes: T = 280; C = 230*	*Total illness duration, days, median (IQR) T = 19 (10–30); C = 14 (6–30)*	Not reported	One study showing a greater risk of experiencing an infection, greater episodes and illness duration overall. Safety not assessed in study; Limited research exists on the effect of vitamin E and respiratory infections Vitamin E is more likely to be included in multivitamin and mineral products for immune health rather than a single ingredient products with such claims
Various zinc formulations up to 30 mg/day in children and adults; in seniors up to 45 mg/day for up to 12 months duration to include the winter months					
1 study involving US cadets during winter months exposed to stressors (n = 40) [56]	No difference in the risk of physician diagnosed infection RR 1.06 (95% CI, 0.34, 3.34; *p* = 0.917)	Number of symptom events: T = 135/238; C = 163/240 *when comparing the subjects* *that had no self-report symptoms during the study to those with* *symptoms, subjects in the Zinc group appreciated more weeks without any symptoms (p = 0.01)*		Not reported	One study in cadets exposed to winter months and physical/academic stressors taking 15 mg zinc experienced less symptom events overall.One study in seniors showed significantly less risk of infections in those taking 45 mg zinc throughout 12 month period. Three of the four studies report benefit for children taking zinc prophylactically throughout the winter months in countries outside the US, up to 20 mg/day. Zinc appears safe in amounts below the upper tolerable limit. It is unclear which formulations of zinc might be most beneficial and for which populations and stressors individuals may experience Studies lack methodological rigor Studies consist of small sample sizes Studies report outcomes in various ways that are not consistent
4 studies involving children living outside the US throughout the winter months (n = 1355) [51,53,54,55]	One study showed less risk of developing symptoms when taking zinc RR 0.78; (95% CI, 0.67, 0.92; *p* = 0.003) One study did not show benefit for experiencing at least one symptom RR 1.02; (95% CI, 0.86, 1.22; *p* = 0.790) Two studies did not report per person but the average cold occurrence was statistically significant in favor of zinc supplement for one study	One study reported on the *mean number of colds per study child: T: 1.2 +* −*1.4; C: 1.7 +* −*1.2; p = 0.003* but the severity of symptoms at day 5 was not significant SMD −0.04 (95% CI, −0.32, 0.24; *p* = 0.79)	Duration of symptoms reduced significantly in two studies: *Duration of cold symptoms: T: 4.7 +* −*0.8; C: 5.3 +* −*0.7;* *MD* −*0.60 (95% CI,* −*0.78,* −*0.42; p = 0.0001); N = 1* *Duration of at least 2 symptoms: median (IQR) T = 0.0 (0–1.0); C = 1.0 (0–5.3) p < 0.01; N = 1* Another study showed *Days missing school: T = 0.55 +*−*1.09; C = 1.35 +* −*1.79;* *MD* −*0.80 (95% CI,* −*1.21,* −*0.39; p < 0.0001*	RD 0.02 (95% CI, −0.11, 0.15; *p* = 0.77, N = 1)	
One study in seniors some of which were zinc-deficient and oxidatively stressed (n = 50) [52]	Significantly less risk of experiencing an infection when taking zinc RR 0.33 (95% CI, 0.17, 0.63; *p* = 0.001)			Not reported	

No published studies were identified meeting the eligibility criteria for astragalus, calcium, ginger, goji, goldenseal, holy basil, licorice, magnesium, melatonin, mangosteen, noni, rose hip, slippery elm, turmeric/curcuma, selenium, vitamin B or vitamin K as single ingredient products for immune health.

## Data Availability

Not applicable.

## Supplementary Materials

The following supporting information can be downloaded at: https://www.mdpi.com/article/10.3390/nu14214604/s1, Supplementary File S1 and Supplementary File S2.

## Disclaimer

The opinions and assertions expressed herein are those of the author(s) and do not reflect the official policy or position of the Uniformed Services University or the Department of Defense. The contents of this publication are the sole responsibility of the author(s) and do not necessarily reflect the views, opinions or policies of the Henry M. Jackson Foundation for the Advancement of Military Medicine. Mention of trade names, commercial products, or organizations does not imply endorsement by the Office of Dietary Supplements, National Institutes of Health, Department of Defense, nor the U.S. Government.
