# Supplementary material for: Select Dietary Supplement Ingredients for Preserving and Protecting the Immune System in Healthy Individuals: A Systematic Review
Source: Nutrients. 2022 Nov 1;14(21):4604. doi: 10.3390/nu14214604 (PMC9655067; doi:10.3390/nu14214604)
Supplement: Supplementary file 1 [file nutrients-14-04604-s001.zip › Supplementary File S1.pdf]

Supplemental File S1 – Scoping reviews

Scope of the market to identify ingredients

The overall goal for this project is to "get ahead of the game" by identifying products and ingredients that are coming into the market and/or forecasted to contribute to increased sales in the immune health dietary supplement market. As such, the authors performed a scoping of the market to identify dietary supplement products and ingredients marketed for immune function in otherwise healthy adults.

The team compiled an internal database of the following ingredients listed in various sources:

- Vitamins, minerals, and botanicals with "immune" claims (Dietary Supplement Label Database (DSLD) Advanced Search – yielded 1742 products with such claims and 79 ingredients that were reported in 20 + products identified consisting of vitamins, minerals and botanicals)
- Top 20 ingredients ranked by immunity sales (Nutrition Business Journal 2020 Immune System Report – communications with journal staff)
- "Immune boosting" ingredients and dietary supplements that appeared in greater than 10% of webpage advertisements based on Google analytics during the 1st and 2nd wave of COVID (BMJ Report, doi:10.1136/bmjopen-2020-040989)
- Popular dietary supplements based on Google search trends related to immunity during the COVID March 2020 outbreak (Hamulka et al. Report, doi.org/10.3390/nu13010054)

Ingredients identified from these sources were first sorted with NBJ-identified ingredients based on (1) the market analysis projections and (2) ingredients listed in and overlapping the multiple sources, as identified above thereafter. Ingredients not mentioned in multiple sources but were identified as listed in more than 100 DSLD products were also considered. This strategy yielded 27 unique dietary supplement ingredients considered to be frequently contained in dietary supplement products with claims related to immune function. **(Table S1)**

Scope of the literature to understand what exists

The authors performed a scoping review to learn what exists in terms of systematic reviews/meta-analysis on the identified ingredients with claims for immune support. What are the types of populations studied, the aims of the research conducted and related outcomes reported?

This exercise was to obtain a general sense of what research exists in terms of these select ingredients related to "immune." Findings helped to inform discussions with the Steering Committee as they relate to the ingredients, aims, and the selection of important outcomes for evaluating the evidence base.

PubMed was searched in July 2021 using MeSH mapping/all fields strategy for each ingredient coupled with "immun*." 548 citations were screened in Covidence at the abstract level based on a broad PICOS (population, ingredient, comparator, outcome, study design as described in **Table S2** below). Studies were excluded for the following reasons. These exclusionary criteria were only for the purpose of scoping the literature and do not dictate eligibility for the subsequent systematic review. For example, while we excluded pregnant and children for the purposes of scoping the literature, we may not do so for the systematic review.

- Did not include adult populations (18+)
- Included pregnant women in relation to their offspring
- Did not mention the dietary supplement ingredient or "immun*" in the abstract
- Focused on levels of vitamins and minerals in the body with no supplementation
- Not a systematic review or meta-analysis

Studies that passed the eligibility criteria were tagged according to the population, aim, and ingredient(s) reported. 159 citations moved to the data extraction phase accordingly. Studies published before 2015 were not included for the full extraction; as such, a total of 106 studies were reviewed. **(Figure S1)** Findings from this scoping were presented to the Steering Committee during a kick-off meeting. **(Tables S3-4)**

Table S1. Dietary supplement ingredients considered frequently contained in dietary supplement products with claims related to immune function

Ingredient	NBJ Market Analysis	DSLD Search for Immune Claims	Google Analytics - Immune Boosting	Google Trends-Immunity during COVID	Available as single ingredient product[1]
Echinacea	x	x		x	x
Elderberry	x	x	x	x	x
Garlic	x	x	x	x	x
Goji juice	x				x
Goldenseal	x	x			x
Mangosteen juice	x				x
Melatonin	x				
Mushrooms	x				x
Noni	x	x			x
Probiotics/Prebiotics	x		x	x	x
Slippery elm	x	x			x
Vitamin A	x	x	x		
Vitamin B	x	x			x
Vitamin C	x	x	x	x	x
Vitamin D	x	x	x	x	x

Zinc	x	x	x	x	x
Ginger		x	x	x	x
Turmeric/curcuma		x	x	x	x
Licorice		x		x	x
Selenium		x		x	x
Vitamin K		x		x	
Astragalus		x			x
Calcium		x			
Holy basil		x			x
Magnesium		x			
Rose hip		x			x
Vitamin E		x			x

[1]Available as single ingredient products on market for immune health (searched DSLD and Amazon)

Table S2. PICOS Eligibility Criteria for *broad* strategy to identify what already exists from systematic reviews

Eligibility Criteria	Broad Eligibility Used to Examine "What Exists"	Search Strategy
Population	Any adults (18+ years old), healthy or unhealthy	No initial restrictions to learn "what exists" on adult populations
Intervention	Any dietary supplement/ingredient defined as any product that met the following criteria: (a) any product (other than tobacco) intended to supplement the diet that contains one or more of the following ingredients: a vitamin, mineral, herb or other botanical, an amino acid, a concentrate, metabolite, constituent, extract or combination of any of these ingredients; (b) administered in any form (e.g., tablet, capsule, soft gel, gel cap, liquid, powder, gel, chews, skin patches/lotions/applications); (c) not represented as conventional food or as a sole item of a meal or of the diet (e.g., sport drinks, shakes, bars); (d) included a "Supplement Facts" rather than "Nutrition Facts" panel	27 select ingredients identified from DSLD, NBJ, Google trends and analytics search strategies chosen<br><br>Specific ingredient names coupled with their scientific names and common names used to create search string or using MESH and all fields if such mapping exists.<br><br>*Combination products included at this time. No restriction to "dietary supplements" in search strategy at this point*

Control/ Comparator(s)	Sham (placebo), no treatment and/or active comparators	No restrictions for scoping
Outcome(s)	Immune function related outcomes	Any review that mentions immune in relation to the ingredient provided in the abstract considered to understand what exists.<br><br>Search strategy included “immun*” in title/abstract
Study Design(s)	We will look for evidence first through quality Systematic Review and Meta-analyses for scoping the literature. Later, a full systematic review will be conducted to include other study designs	Search filters used to target systematic reviews/meta-analyses before moving into other study designs
Timing	We are interested in any timing	No restrictions for scoping

Search Strategy Executed in PubMed

(Echinacea or elderberry or garlic or goji berry or "goji juice" or goldenseal or hydrastis or mangosteen or garcinia mangostana or melatonin or mushroom or agaricales or noni or morinda or probiotic or prebiotic or slippery elm or ulmus or vitamin a or vitamin b or vitamin c or vitamin d or zinc or ginger or turmeric or curcuma or licorice or glycyrrhiza or selenium or vitamin k or astragalus or astragalus plant or calcium or holy basil or ocimum sanctum or magnesium or "rose hip" or rosehip or "Rosa"[Mesh] or vitamin e) AND (immun[Title/Abstract])*

Filters: Meta-Analysis, Systematic Review

Figure S1. PRISMA Flowchart

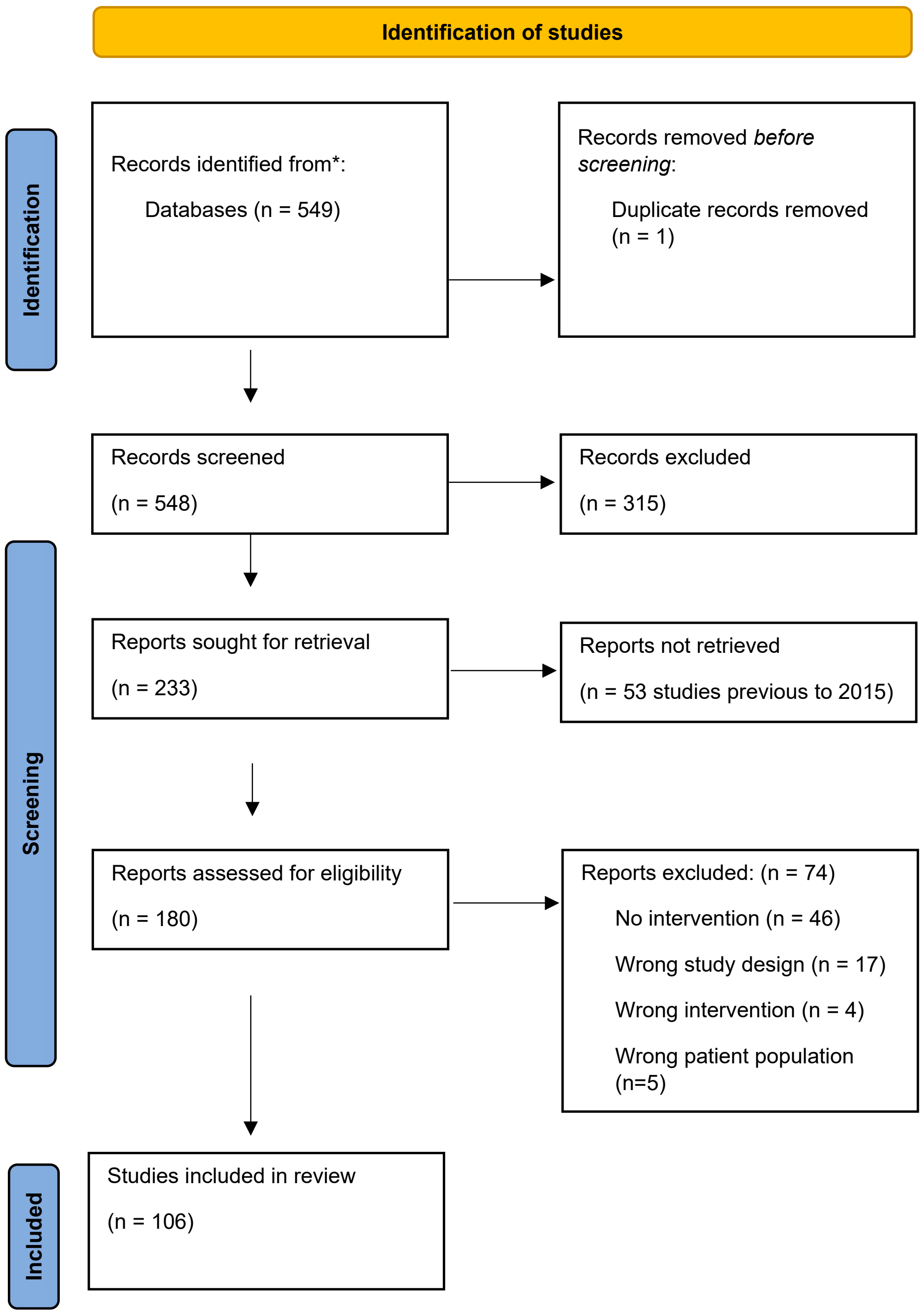

From: Page MJ, McKenzie JE, Bossuyt PM, Boutron I, Hoffmann TC, Mulrow CD, et al. The PRISMA 2020 statement: an updated guideline for reporting systematic reviews. BMJ 2021;372:n71. doi: 10.1136/bmj.n71

Table S3. Findings from Scoping Review of the Literature

This table displays the types of populations (i.e., healthy, those with respiratory condition, inflammatory condition, or another disease) reported among review articles for each select ingredient as well as the study's aim (i.e., prevention, treatment, performance/resilience).

Across all ingredients, 17% studies looked at otherwise healthy adults, including those under a stressor, vaccination, athletes, physically active individuals, and elderly; 24% studies looked at the prevention or treatment of respiratory conditions such as the common cold, viral and respiratory infections, influenza, coronavirus, allergic rhinitis, pneumonia, and tuberculosis; 25% studies looked at patients with inflammatory conditions such as atopic dermatitis, periodontal diseases, irritable bowel disease, ulcerative colitis, rheumatoid arthritis, and HIV; 30% unhealthy - other diseased including conditions such as cancer, liver disease, MS, cystic fibrosis and heart disease; and 4% of studies included data on safety and harms, with populations otherwise not specified. *Note that this scoping review only captured studies that included "immun*" in the titles/abstracts and is not representative of all studies for any condition.*

Ingredient	Healthy	Respiratory condition	Inflammatory condition	Unhealthy - other diseased
**Astragalus**			Treatment	
**Calcium**			Prevention; Treatment	
**Combination formulations**		Prevention; Treatment		
**Echinacea**				
**Elderberry**		Prevention; Treatment		
**Garlic**		Prevention; Treatment		Treatment
**Ginger**				
**Goji**				
**Goldenseal**				
**Holy basil**	Prevention			Treatment

**Licorice**				Treatment
**Mangosteen**				
**Magnesium**				Treatment
**Melatonin**		Prevention; Treatment	Prevention; Treatment	Prevention
**Mushrooms**				Treatment
**Noni**				
**Prebiotics**	Performance; Prevention	Prevention	Treatment	
**Probiotics**	Performance; Prevention	Prevention; Treatment	Prevention; Treatment	Prevention; Treatment
**Rose hip**				
**Selenium**		Prevention; Treatment	Treatment	Treatment
**Slippery elm**				
**Turmeric**			Treatment	Treatment
**Vitamin A**	Prevention	Prevention; Treatment	Treatment	Treatment
**Vitamin B**	Prevention	Prevention; Treatment	Treatment	Treatment
**Vitamin C**	Prevention	Prevention; Treatment		Treatment
**Vitamin D**	Prevention	Prevention; Treatment	Treatment	Prevention; Treatment
**Vitamin E**	Prevention	Prevention; Treatment	Treatment	
**Vitamin K**				
**Zinc**	Prevention	Prevention; Treatment	Treatment	Treatment

Table S4a. Outcomes Reported in Performance Studies

Across studies and represented in **Tables 4a-c**, biomarkers accounted for approximately half of outcomes reported on in the systematic reviews, with others related more toward clinical-type outcomes. Some of the most common biomarkers were c-reactive protein (CRP), interleukins (ILs), interferons (IFN), tumor necrosis factor (TNF), immunoglobulins (Ig), and cytokines across the different types of studies. Some of the most common other types of outcomes were related to symptom onset, symptom duration, severity, mortality and adverse events/safety. Most commonly reported are in bold throughout the tables below.

Population	Biomarkers	Other
**Healthy**	cellular innate immune activity<br>**C-reactive protein (CRP)**<br>**cytokines**<br>detection of probiotics in stool<br>immune markers<br>immunoglobulins: SIgA<br>inflammatory markers<br>**interferons (IFNs)**<br>**interleukins (IL)**<br>metabolic markers<br>NK cell tumoricidal activity<br>prostaglandins<br>**TNF/TNF-a**<br>**white blood cells: monocytes, phagocytes,**<br>**lymphocytes, macrophages**	cognition<br>frailty<br>gastrointestinal symptoms<br>immune system modulation/performance<br>mood<br>**performance (time to exhaustion)**<br>physical strength<br>receipt of care

Table S4b. Outcomes Reported in Prevention Studies

Population	Biomarkers	Other
**Healthy**	blood glucose<br>blood lipids: CD3, CD4<br>**C-reactive protein (CRP)**<br>creatine kinase (CK) levels<br>**cytokines**<br>detection of probiotics in stool<br>**immunoglobulins: IgA, IgE, IgG, IgM**<br>inflammatory markers<br>**interferons (IFNs): IFN-g**<br>**interleukins (IL): IL-4, IL-10**<br>lipid profile<br>metabolic markers<br>NK cell tumoricidal activity<br>**nutritional status markers**<br>prostaglandins<br>**TNF, TNF-a**<br>vaccines responses<br>**white blood cells: monocytes, lymphocytes, phagocyte, macrophages**	adverse events<br>blood pressure<br>**cognitive function**<br>digestive health<br>**disease symptoms**<br>frailty<br>gastrointestinal symptoms<br>general well-being<br>**infections**<br>insulin sensitivity/resistance<br>microbiota composition<br>mood<br>mortality<br>neurocognitive changes<br>**nutrient dietary intake**<br>**receipt of care**<br>**vaccine performance**
**Respiratory condition**	**allergy markers**<br>biochemical markers<br>**cellular changes**<br>**c-reactive protein (CRP)**<br>**cytokines**<br>endothelial function<br>hemagglutination inhibition (HI)<br>antibody titers<br>**interleukins: IL6**<br>immune cell phenotype<br>**immunoglobulins: IgE**<br>**immunological outcomes**<br>**immunological response to vaccination**<br>**inflammatory serum markers**<br>interferons (IFNs)<br>lymphocyte<br>serological response<br>**TNF**<br>viral load	absences due to disease<br>**adverse events**<br>clinical manifestations<br>compliance<br>**disease duration**<br>**disease episodes**<br>**disease severity**<br>**disease symptoms**<br>**disease/infection occurrence**<br>**hospitalizations - incidence/duration**<br>ICU - admission<br>**incidence of viral RTIs**<br>**infection - incidence/risk**<br>**medication usage**<br>mortality<br>number of sick days<br>quality of life<br>risk of complications<br>side effects<br>time to improvement<br>ventilation/intubation - incidence/duration
**Unhealthy**	cholesterol - HDL, LDL, total<br>triglycerides (TG)	**disease onset**<br>**disease progression**

	**immunoglobulins: IgE**	ICU - duration<br>incidence of delirium<br>pulmonary function<br>risk of symptoms<br>symptom management<br>symptom severity
**Inflammatory**	antioxidant status<br>blood glucose<br>carbohydrate metabolism<br>cell proliferation<br>composition of the intestinal microbiota<br>**c-reactive protein (CRP)**<br>**cytokines**<br>immune responses<br>insulin sensitivity<br>**interleukins**<br>metabolic activity of the intestinal microbiota<br>metabolic stress<br>oxidative stress<br>**TNF**	adverse events<br>BMI<br>**clinical symptoms**<br>fat mass<br>inflammation<br>intestinal barrier function<br>production of compounds with anti-carcinogenic activity<br>remission/relapse<br>symptom management<br>tumor growth

Table S4c. Outcomes Reported in Treatment Studies

Population	Biomarkers	Other
**Respiratory condition**	**allergy markers**<br>**cellular changes**<br>**c-reactive protein (CRP), hs-CRP**<br>**cytokines**<br>erythrocyte sedimentation rate (ESR)<br>hemoglobin<br>**immunoglobulins: IgA, IgE**<br>**inflammatory markers**<br>**interferons (IFNs): IFN-g**<br>**interleukins: IL-6, IL-10**<br>plasma levels<br>serum levels<br>**TNF**<br>**white blood cells: monocytes, lymphocytes, neutrophil**	**adverse events**<br>**BMI**<br>**body measurements**<br>chest imaging<br>clinical deterioration<br>cognitive function<br>**condition-specific symptoms**<br>**cure**<br>**disease duration**<br>**disease incidence**<br>**disease severity**<br>dyspnea<br>fatigue<br>**hospitalization - duration/incidence**<br>**ICU admission - duration/incidence**<br>infection/illness<br>insomnia<br>medication use<br>**mortality**<br>pain<br>physical function<br>**quality of life**<br>radiological examinations<br>risk of complications<br>sick days<br>TB score<br>time to improvement<br>time to recovery<br>vaccine response<br>ventilation/intubation - frequency/incidence
**Unhealthy**	adiponectin<br>anti-tumor immune response<br>blood lipid profile (cholesterol: HDL, LDL, total)<br>**c-reactive protein (CRP), hs-CRP**<br>creatine kinase (CK) levels<br>**cytokines**<br>endotoxin<br>**Glucose measures**<br>glutathione peroxidase (GSH-PX)<br>glycemic measures<br>**immune function parameters**<br>**immunoglobulins: IgA, IgE, IgG, IgM**	adverse events<br>balance<br>blood pressure<br>body weight<br>bone disease-related outcomes<br>CHD mortality<br>clinical status<br>disease activity<br>disease complication<br>**disease duration**<br>**disease incidence**<br>**disease onset**<br>**disease progression**

	**inflammatory parameters**<br>insulin sensitivity measures<br>**interferons (IFNs)**<br>**interleukins: IL-6, IL-10**<br>leptin<br>lipid profile<br>NK-cell activity<br>nutritional status markers<br>serum levels<br>**TNF**<br>triglycerides (TG)<br>**white blood cells: T-lymphocytes**	**disease severity**<br>**disease-related symptoms**<br>disorder status<br>fatigue<br>**health-related quality of life**<br>hepatic fibrosis<br>hepatic function<br>hospitaliation - duration<br>ICU - duration<br>immune depression<br>immune function/response<br>immunological status-related outcomes<br>infections<br>liver function<br>long-term survival<br>malignant transformation<br>**mortality**<br>need for additional therapy<br>neurocognitive changes<br>performance status/score<br>pulmonary exacerbations<br>pulmonary function<br>quality of life<br>relapse rates<br>respiratory status-related outcomes<br>risk of illness<br>tumour response<br>ventilation - duration<br>wound healing
**Inflammatory**	antioxidant status<br>blood glucose<br>carbohydrate metabolism<br>**CD4 + cells**<br>C-reactive protein (CRP)<br>**cytokines**<br>erythrocyte sedimentation rate<br>immunomodulatory effects<br>insulin sensitivity<br>**interleukins: IL2, IL-6, IL-10, IL-12**<br>metabolic stress<br>oxidative stress indices: antioxidant capacity,<br>malondialdehyde<br>serum levels<br>serum thyroid peroxidase (TPOAb)<br>Th17Treg cells<br>thyroglobulin (TgAb) autoantibody levels<br>**TNF**	body mass index<br>diarrhea management<br>**disease activity**<br>**disease progression**<br>**disease severity**<br>fat mass<br>hypovitaminosis<br>infection levels/risk<br>**mortality**<br>oral lesions<br>pain severity<br>remission/relapse rates<br>safety<br>swollen joint count<br>symptom management<br>tender joint count<br>**nutrient dietary intake**

References from Scoping

1. Zhao W, Peng C, Sakandar HA, Kwok LY, Zhang W. Meta-Analysis: Randomized Trials of Lactobacillus plantarum on Immune Regulation Over the Last Decades. *Front Immunol.* 2021;12:643420. 10.3389/fimmu.2021.643420

2. Yang C, Lu Y, Wan M, et al. Efficacy of High-Dose Vitamin D Supplementation as an Adjuvant Treatment on Pneumonia: Systematic Review and a Meta-Analysis of Randomized Controlled Studies. *Nutr Clin Pract.* 2021;36(2):368-384. 10.1002/ncp.10585

3. Wieland LS, Piechotta V, Feinberg T, et al. Elderberry for prevention and treatment of viral respiratory illnesses: a systematic review. *BMC Complement Med Ther.* 2021;21(1):112. 10.1186/s12906-021-03283-5

4. Vollbracht C, Kraft K. Feasibility of Vitamin C in the Treatment of Post Viral Fatigue with Focus on Long COVID, Based on a Systematic Review of IV Vitamin C on Fatigue. *Nutrients.* 2021;13(4). 10.3390/nu13041154

5. Taghipour A, Abdoli A, Ramezani A, et al. Leishmaniasis and Trace Element Alterations: a Systematic Review. *Biol Trace Elem Res.* 2021. 10.1007/s12011-020-02505-0

6. Strauss M, Mičetić-Turk D, Pogačar M, Fijan S. Probiotics for the Prevention of Acute Respiratory-Tract Infections in Older People: Systematic Review. *Healthcare (Basel).* 2021;9(6). 10.3390/healthcare9060690

7. Steimbach L, Borgmann AV, Gomar GG, et al. Fungal beta-glucans as adjuvants for treating cancer patients - A systematic review of clinical trials. *Clin Nutr.* 2021;40(5):3104-3113. 10.1016/j.clnu.2020.11.029

8. Shi HY, Zhu X, Li WL, et al. Modulation of gut microbiota protects against viral respiratory tract infections: a systematic review of animal and clinical studies. *Eur J Nutr.* 2021:1-24. 10.1007/s00394-021-02519-x

9. Rawat D, Roy A, Maitra S, Shankar V, Khanna P, Baidya DK. "Vitamin D supplementation and COVID-19 treatment: A systematic review and meta-analysis". *Diabetes Metab Syndr.* 2021;15(4):102189. 10.1016/j.dsx.2021.102189

10. Petrelli F, Luciani A, Perego G, Dognini G, Colombelli PL, Ghidini A. Therapeutic and prognostic role of vitamin D for COVID-19 infection: A systematic review and meta-analysis of 43 observational studies. *J Steroid Biochem Mol Biol.* 2021;211:105883. 10.1016/j.jsbmb.2021.105883

11. Ng E, Tay JRH, Saffari SE, Lim LP, Chung KM, Ong MMA. Adjunctive probiotics after periodontal debridement versus placebo: a systematic review and meta-analysis. *Acta Odontol Scand.* 2021:1-10. 10.1080/00016357.2021.1942193

12. Lv Q, Liang X, Nong K, et al. Advances in Research on the Toxicological Effects of Selenium. *Bull Environ Contam Toxicol.* 2021;106(5):715-726. 10.1007/s00128-020-03094-3

13. Łagowska K, Bajerska J. Effects of probiotic supplementation on respiratory infection and immune function in athletes: systematic review and meta-analysis of randomized controlled trials. *J Athl Train.* 2021. 10.4085/592-20

14. Hutchinson AN, Bergh C, Kruger K, et al. The Effect of Probiotics on Health Outcomes in the Elderly: A Systematic Review of Randomized, Placebo-Controlled Studies. *Microorganisms.* 2021;9(6). 10.3390/microorganisms9061344

15. Hoppe C, Kutschan S, Dörfler J, Büntzel J, Büntzel J, Huebner J. Zinc as a complementary treatment for cancer patients: a systematic review. *Clin Exp Med.* 2021;21(2):297-313. 10.1007/s10238-020-00677-6

16. Fernández-Palanca P, Méndez-Blanco C, Fondevila F, et al. Melatonin as an Antitumor Agent against Liver Cancer: An Updated Systematic Review. *Antioxidants (Basel).* 2021;10(1). 10.3390/antiox10010103

17. Darbandi A, Asadi A, Ghanavati R, et al. The effect of probiotics on respiratory tract infection with special emphasis on COVID-19: Systemic review 2010-20. *Int J Infect Dis.* 2021;105:91-104. 10.1016/j.ijid.2021.02.011

18. Corrao S, Mallaci Bocchio R, Lo Monaco M, et al. Does Evidence Exist to Blunt Inflammatory Response by Nutraceutical Supplementation during COVID-19 Pandemic? An Overview of Systematic Reviews of Vitamin D, Vitamin C, Melatonin, and Zinc. *Nutrients.* 2021;13(4). 10.3390/nu13041261

19. Cho JH, Bhutani S, Kim CH, Irwin MR. Anti-inflammatory effects of melatonin: A systematic review and meta-analysis of clinical trials. *Brain Behav Immun.* 2021;93:245-253. 10.1016/j.bbi.2021.01.034

20. Bassatne A, Basbous M, Chakhtoura M, El Zein O, Rahme M, El-Hajj Fuleihan G. The link between COVID-19 and VItamin D (VIVID): A systematic review and meta-analysis. *Metabolism.* 2021;119:154753. 10.1016/j.metabol.2021.154753

21. Askari G, Ghavami A, Shahdadian F, Moravejolahkami AR. Effect of synbiotics and probiotics supplementation on autoimmune diseases: A systematic review and meta-analysis of clinical trials. *Clin Nutr.* 2021;40(5):3221-3234. 10.1016/j.clnu.2021.02.015

22. Abioye AI, Bromage S, Fawzi W. Effect of micronutrient supplements on influenza and other respiratory tract infections among adults: a systematic review and meta-analysis. *BMJ Glob Health.* 2021;6(1). 10.1136/bmjgh-2020-003176

23. Zhu Y, Jiang Z, Huang H, et al. Assessment of Melatonergics in Prevention of Delirium: A Systematic Review and Meta-Analysis. *Front Neurol.* 2020;11:198. 10.3389/fneur.2020.00198

24. Zhu M, Liu X, Zhou C, Li J. Effect of sodium cantharidinate/vitamin B6 injection on survival, liver function, immune function, and quality of life in patients with hepatocellular carcinoma: Protocol for a meta-analysis. *Medicine (Baltimore).* 2020;99(34):e21952. 10.1097/md.0000000000021952

25. Xu YY, Wang DM, Liang HS, et al. The Role of Th17/Treg Axis in the Traditional Chinese Medicine Intervention on Immune-Mediated Inflammatory Diseases: A Systematic Review. *Am J Chin Med.* 2020;48(3):535-558. 10.1142/s0192415x20500275

26. Tan HK, Streeter A, Cramp ME, Dhanda AD. Effect of zinc treatment on clinical outcomes in patients with liver cirrhosis: A systematic review and meta-analysis. *World J Hepatol.* 2020;12(7):389-398. 10.4254/wjh.v12.i7.389

27. Salles BIM, Cioffi D, Ferreira SRG. Probiotics supplementation and insulin resistance: a systematic review. *Diabetol Metab Syndr.* 2020;12(1):98. 10.1186/s13098-020-00603-6

28. Poon T, Juana J, Noori D, Jeansen S, Pierucci-Lagha A, Musa-Veloso K. Effects of a Fermented Dairy Drink Containing Lacticaseibacillus paracasei subsp. paracasei CNCM I-1518 (Lactobacillus casei CNCM I-1518) and the Standard Yogurt Cultures on the Incidence, Duration, and Severity of Common Infectious Diseases: A Systematic Review and Meta-Analysis of Randomized Controlled Trials. *Nutrients.* 2020;12(11). 10.3390/nu12113443

29. Pellow J, Nolte A, Temane A, Solomon EM. Health supplements for allergic rhinitis: A mixed-methods systematic review. *Complement Ther Med.* 2020;51:102425. 10.1016/j.ctim.2020.102425

30. Paknahad Z, Moravejolahkami AR. Probiotics against viruses; COVID-19 is a paper tiger: A Systematic Review. *Endocr Metab Immune Disord Drug Targets.* 2020. 10.2174/1871530320666200917114033

31. Pabón-Carrasco M, Ramirez-Baena L, Vilar-Palomo S, Castro-Méndez A, Martos-García R, Rodríguez-Gallego I. Probiotics as a Coadjuvant Factor in Active or Quiescent Inflammatory Bowel Disease of Adults-A Meta-Analytical Study. *Nutrients.* 2020;12(9). 10.3390/nu12092628

32. Mohr AE, Basile AJ, Crawford MS, Sweazea KL, Carpenter KC. Probiotic Supplementation Has a Limited Effect on Circulating Immune and Inflammatory Markers in Healthy Adults: A Systematic Review of Randomized Controlled Trials. *J Acad Nutr Diet.* 2020;120(4):548-564. 10.1016/j.jand.2019.08.018

33. Mirabile A, Rivoltini L, Daveri E, et al. Metabolism and Immune Modulation in Patients with Solid Tumors: Systematic Review of Preclinical and Clinical Evidence. *Cancers (Basel).* 2020;12(5). 10.3390/cancers12051153

34. Mata IRD, Mata SRD, Menezes RCR, Faccioli LS, Bandeira KK, Bosco SMD. Benefits of turmeric supplementation for skin health in chronic diseases: a systematic review. *Crit Rev Food Sci Nutr.* 2020:1-15. 10.1080/10408398.2020.1798353

35. Kazemi A, Soltani S, Nasri F, Clark CCT, Kolahdouz-Mohammadi R. The effect of probiotics, parabiotics, synbiotics, fermented foods and other microbial forms on immunoglobulin production: a systematic review and meta-analysis of clinical trials. *Int J Food Sci Nutr.* 2020:1-19. 10.1080/09637486.2020.1857710

36. Kaur L, Gordon M, Baines PA, Iheozor-Ejiofor Z, Sinopoulou V, Akobeng AK. Probiotics for induction of remission in ulcerative colitis. *Cochrane Database Syst Rev.* 2020;3(3):Cd005573. 10.1002/14651858.CD005573.pub3

37. Juhász MF, Varannai O, Németh D, et al. Vitamin D supplementation in patients with cystic fibrosis: A systematic review and meta-analysis. *J Cyst Fibros.* 2020. 10.1016/j.jcf.2020.12.008

38. Jiang D, Bai CX, Chen O. [Roles of Probiotic Supplementation in the Prevention and Treatment of Asthma:A Systematic Review]. *Zhongguo Yi Xue Ke Xue Yuan Xue Bao.* 2020;42(2):178-189. 10.3881/j.issn.1000-503X.11346

39. Jayawardena R, Sooriyaarachchi P, Chourdakis M, Jeewandara C, Ranasinghe P. Enhancing immunity in viral infections, with special emphasis on COVID-19: A review. *Diabetes Metab Syndr.* 2020;14(4):367-382. 10.1016/j.dsx.2020.04.015

40. Jafari A, Noormohammadi Z, Askari M, Daneshzad E. Zinc supplementation and immune factors in adults: a systematic review and meta-analysis of randomized clinical trials. *Crit Rev Food Sci Nutr.* 2020:1-19. 10.1080/10408398.2020.1862048

41. Iheozor-Ejiofor Z, Kaur L, Gordon M, Baines PA, Sinopoulou V, Akobeng AK. Probiotics for maintenance of remission in ulcerative colitis. *Cochrane Database Syst Rev.* 2020;3(3):Cd007443. 10.1002/14651858.CD007443.pub3

42. Hurley MN, Smith S, Forrester DL, Smyth AR. Antibiotic adjuvant therapy for pulmonary infection in cystic fibrosis. *Cochrane Database Syst Rev.* 2020;7:Cd008037. 10.1002/14651858.CD008037.pub4

43. Ho SN, Acharya A, Sidharthan S, et al. A Systematic Review and Meta-analysis of Clinical, Immunological, and Microbiological Shift in Periodontitis After Nonsurgical Periodontal Therapy With Adjunctive Use of Probiotics. *J Evid Based Dent Pract.* 2020;20(1):101397. 10.1016/j.jebdp.2020.101397

44. Ebrahimpour-Koujan S, Milajerdi A, Larijani B, Esmaillzadeh A. Effects of probiotics on salivary cytokines and immunoglobulines: a systematic review and meta-analysis on clinical trials. *Sci Rep.* 2020;10(1):11800. 10.1038/s41598-020-67037-y

45. Cruz BCS, Sarandy MM, Messias AC, Gonçalves RV, Ferreira C, Peluzio MCG. Preclinical and clinical relevance of probiotics and synbiotics in colorectal carcinogenesis: a systematic review. *Nutr Rev.* 2020;78(8):667-687. 10.1093/nutrit/nuz087

46. Coutts L, Ibrahim K, Tan QY, Lim SER, Cox NJ, Roberts HC. Can probiotics, prebiotics and synbiotics improve functional outcomes for older people: a systematic review. *Eur Geriatr Med.* 2020;11(6):975-993. 10.1007/s41999-020-00396-x

47. Chen T, Zuo X, Wang S, et al. The effect of vitamin D supplementation on the progression of fibrosis in patients with chronic liver disease: A protocol for a systematic review and meta-analysis. *Medicine (Baltimore).* 2020;99(19):e20296. 10.1097/md.0000000000020296

48. Calero CDQ, Rincón EO, Marqueta PM. Probiotics, prebiotics and synbiotics: useful for athletes and active individuals? A systematic review. *Benef Microbes.* 2020;11(2):135-149. 10.3920/bm2019.0076

49. Byrne DF, Geraghty AA, Yelverton CA, et al. The impact of probiotic supplementation on metabolic health in healthy women of reproductive age: a systematic review. *Food Funct.* 2020;11(12):10279-10289. 10.1039/d0fo01727b

50. Zhang J, Chen C, Yang J. Effectiveness of vitamin D supplementation on the outcome of pulmonary tuberculosis treatment in adults: a meta-analysis of randomized controlled trials. *Chin Med J (Engl).* 2019;132(24):2950-2959. 10.1097/cm9.0000000000000554

51. van Gorkom GNY, Lookermans EL, Van Elssen C, Bos GMJ. The Effect of Vitamin C (Ascorbic Acid) in the Treatment of Patients with Cancer: A Systematic Review. *Nutrients.* 2019;11(5). 10.3390/nu11050977

52. Teixeira N, Pereira BM, Oliveira IKF, et al. Effect of vitamin D3 supplementation on HIV-infected adults: a systematic reviewVitamin D3 Supplementation on HIV-Infected Adults: A Systematic Review. *Nutr Hosp.* 2019;36(5):1205-1212. 10.20960/nh.02647

53. Reynolds KA, Juhasz MLW, Mesinkovska NA. The role of oral vitamins and supplements in the management of atopic dermatitis: a systematic review. *Int J Dermatol.* 2019;58(12):1371-1376. 10.1111/ijd.14404

54. Picó-Monllor JA, Mingot-Ascencao JM. Search and Selection of Probiotics That Improve Mucositis Symptoms in Oncologic Patients. A Systematic Review. *Nutrients.* 2019;11(10). 10.3390/nu11102322

55. Morshedi M, Hashemi R, Moazzen S, Sahebkar A, Hosseinifard ES. Immunomodulatory and anti-inflammatory effects of probiotics in multiple sclerosis: a systematic review. *J Neuroinflammation.* 2019;16(1):231. 10.1186/s12974-019-1611-4

56. Möller GB, da Cunha Goulart MJV, Nicoletto BB, Alves FD, Schneider CD. Supplementation of Probiotics and Its Effects on Physically Active Individuals and Athletes: Systematic Review. *Int J Sport Nutr Exerc Metab.* 2019;29(5):481-492. 10.1123/ijsnem.2018-0227

57. Miller LE, Lehtoranta L, Lehtinen MJ. Short-term probiotic supplementation enhances cellular immune function in healthy elderly: systematic review and meta-analysis of controlled studies. *Nutr Res.* 2019;64:1-8. 10.1016/j.nutres.2018.12.011

58. Lv KJ, Chen TC, Wang GH, Yao YN, Yao H. Clinical safety and efficacy of curcumin use for oral lichen planus: a systematic review. *J Dermatolog Treat.* 2019;30(6):605-611. 10.1080/09546634.2018.1543849

59. Karimi A, Ghodsi R, Kooshki F, Karimi M, Asghariazar V, Tarighat-Esfanjani A. Therapeutic effects of curcumin on sepsis and mechanisms of action: A systematic review of preclinical studies. *Phytother Res.* 2019;33(11):2798-2820. 10.1002/ptr.6467

60. Gray NA, Dhana A, Stein DJ, Khumalo NP. Zinc and atopic dermatitis: a systematic review and meta-analysis. *J Eur Acad Dermatol Venereol.* 2019;33(6):1042-1050. 10.1111/jdv.15524

61. Gadelha C, Bezerra AN. Effects of probiotics on the lipid profile: systematic review. *J Vasc Bras.* 2019;18:e20180124. 10.1590/1677-5449.180124

62. Church JA, Parker EP, Kirkpatrick BD, Grassly NC, Prendergast AJ. Interventions to improve oral vaccine performance: a systematic review and meta-analysis. *Lancet Infect Dis.* 2019;19(2):203-214. 10.1016/s1473-3099(18)30602-9

63. Azimi A, Ghajarzadeh M, Sahraian MA, et al. Effects of Vitamin D Supplements on IL-10 and INFγ Levels in Patients with Multiple Sclerosis: a Systematic Review and Meta-Analysis. *Maedica (Bucur).* 2019;14(4):413-417. 10.26574/maedica.2019.14.4.413

64. Zimmermann P, Curtis N. The influence of probiotics on vaccine responses - A systematic review. *Vaccine.* 2018;36(2):207-213. 10.1016/j.vaccine.2017.08.069

65. Yeh TL, Shih PC, Liu SJ, et al. The influence of prebiotic or probiotic supplementation on antibody titers after influenza vaccination: a systematic review and meta-analysis of randomized controlled trials. *Drug Des Devel Ther.* 2018;12:217-230. 10.2147/dddt.S155110

66. Wu HX, Xiong XF, Zhu M, Wei J, Zhuo KQ, Cheng DY. Effects of vitamin D supplementation on the outcomes of patients with pulmonary tuberculosis: a systematic review and meta-analysis. *BMC Pulm Med.* 2018;18(1):108. 10.1186/s12890-018-0677-6

67. Wang L, Song Y. Efficacy of zinc given as an adjunct to the treatment of severe pneumonia: A meta-analysis of randomized, double-blind and placebo-controlled trials. *Clin Respir J.* 2018;12(3):857-864. 10.1111/crj.12646

68. Varela-López A, Navarro-Hortal MD, Giampieri F, Bullón P, Battino M, Quiles JL. Nutraceuticals in Periodontal Health: A Systematic Review on the Role of Vitamins in Periodontal Health Maintenance. *Molecules.* 2018;23(5). 10.3390/molecules23051226

69. Skonieczna-Żydecka K, Kaczmarczyk M, Łoniewski I, et al. A Systematic Review, Meta-Analysis, and Meta-Regression Evaluating the Efficacy and Mechanisms of Action of Probiotics and Synbiotics in the Prevention of Surgical Site Infections and Surgery-Related Complications. *J Clin Med.* 2018;7(12). 10.3390/jcm7120556

70. Jagannath VA, Filippini G, Di Pietrantonj C, et al. Vitamin D for the management of multiple sclerosis. *Cochrane Database Syst Rev.* 2018;9(9):Cd008422. 10.1002/14651858.CD008422.pub3

71. Costa RL, Moreira J, Lorenzo A, Lamas CC. Infectious complications following probiotic ingestion: a potentially underestimated problem? A systematic review of reports and case series. *BMC Complement Altern Med.* 2018;18(1):329. 10.1186/s12906-018-2394-3

72. Aqaeinezhad Rudbane SM, Rahmdel S, Abdollahzadeh SM, Zare M, Bazrafshan A, Mazloomi SM. The efficacy of probiotic supplementation in rheumatoid arthritis: a meta-analysis of randomized, controlled trials. *Inflammopharmacology.* 2018;26(1):67-76. 10.1007/s10787-017-0436-y

73. Visser ME, Durao S, Sinclair D, Irlam JH, Siegfried N. Micronutrient supplementation in adults with HIV infection. *Cochrane Database Syst Rev.* 2017;5(5):Cd003650. 10.1002/14651858.CD003650.pub4

74. Simadibrata M, Halimkesuma CC, Suwita BM. Efficacy of Curcumin as Adjuvant Therapy to Induce or Maintain Remission in Ulcerative Colitis Patients: an Evidence-based Clinical Review. *Acta Med Indones.* 2017;49(4):363-368.

75. Oliveira ALG, Chaves AT, Menezes CAS, et al. Vitamin D receptor expression and hepcidin levels in the protection or severity of leprosy: a systematic review. *Microbes Infect.* 2017;19(6):311-322. 10.1016/j.micinf.2017.03.001

76. Mohammed AT, Khattab M, Ahmed AM, et al. The therapeutic effect of probiotics on rheumatoid arthritis: a systematic review and meta-analysis of randomized control trials. *Clin Rheumatol.* 2017;36(12):2697-2707. 10.1007/s10067-017-3814-3

77. Miller LE, Lehtoranta L, Lehtinen MJ. The Effect of Bifidobacterium animalis ssp. lactis HN019 on Cellular Immune Function in Healthy Elderly Subjects: Systematic Review and Meta-Analysis. *Nutrients.* 2017;9(3). 10.3390/nu9030191

78. Lei WT, Shih PC, Liu SJ, Lin CY, Yeh TL. Effect of Probiotics and Prebiotics on Immune Response to Influenza Vaccination in Adults: A Systematic Review and Meta-Analysis of Randomized Controlled Trials. *Nutrients.* 2017;9(11). 10.3390/nu9111175

79. Ju W, Li X, Li Z, et al. The effect of selenium supplementation on coronary heart disease: A systematic review and meta-analysis of randomized controlled trials. *J Trace Elem Med Biol.* 2017;44:8-16. 10.1016/j.jtemb.2017.04.009

80. Jamshidi N, Cohen MM. The Clinical Efficacy and Safety of Tulsi in Humans: A Systematic Review of the Literature. *Evid Based Complement Alternat Med.* 2017;2017:9217567. 10.1155/2017/9217567

81. Jalloh MA, Gregory PJ, Hein D, Risoldi Cochrane Z, Rodriguez A. Dietary supplement interactions with antiretrovirals: a systematic review. *Int J STD AIDS.* 2017;28(1):4-15. 10.1177/0956462416671087

82. Franco AS, Freitas TQ, Bernardo WM, Pereira RMR. Vitamin D supplementation and disease activity in patients with immune-mediated rheumatic diseases: A systematic review and meta-analysis. *Medicine (Baltimore).* 2017;96(23):e7024. 10.1097/md.0000000000007024

83. Fernandes R, do Rosario VA, Mocellin MC, Kuntz MGF, Trindade E. Effects of inulin-type fructans, galacto-oligosaccharides and related synbiotics on inflammatory markers in adult patients with overweight or obesity: A systematic review. *Clin Nutr.* 2017;36(5):1197-1206. 10.1016/j.clnu.2016.10.003

84. Enomoto YM, Nakamura YMP, Enomoto NMP, Fujisawa TMP, Inui NMP, Suda T. Japanese herbal medicine-induced pneumonitis: A review of 73 patients. *Respir Investig.* 2017;55(2):138-144. 10.1016/j.resinv.2016.11.007

85. Autier P, Mullie P, Macacu A, et al. Effect of vitamin D supplementation on non-skeletal disorders: a systematic review of meta-analyses and randomised trials. *Lancet Diabetes Endocrinol.* 2017;5(12):986-1004. 10.1016/s2213-8587(17)30357-1

86. Ai R, Wei J, Ma D, et al. A meta-analysis of randomized trials assessing the effects of probiotic preparations on oral candidiasis in the elderly. *Arch Oral Biol.* 2017;83:187-192. 10.1016/j.archoralbio.2017.04.030

87. Abdo J, Rai V, Agrawal DK. Interplay of Immunity and Vitamin D: Interactions and Implications with Current IBD Therapy. *Curr Med Chem.* 2017;24(9):852-867. 10.2174/0929867323666161026124951

88. Zuk A, Fitzpatrick T, Rosella LC. Effect of Vitamin D3 Supplementation on Inflammatory Markers and Glycemic Measures among Overweight or Obese Adults: A Systematic Review of Randomized Controlled Trials. *PLoS One.* 2016;11(4):e0154215. 10.1371/journal.pone.0154215

89. Wichman J, Winther KH, Bonnema SJ, Hegedüs L. Selenium Supplementation Significantly Reduces Thyroid Autoantibody Levels in Patients with Chronic Autoimmune Thyroiditis: A Systematic Review and Meta-Analysis. *Thyroid.* 2016;26(12):1681-1692. 10.1089/thy.2016.0256

90. Varela-López A, Giampieri F, Bullón P, Battino M, Quiles JL. A Systematic Review on the Implication of Minerals in the Onset, Severity and Treatment of Periodontal Disease. *Molecules.* 2016;21(9). 10.3390/molecules21091183

91. Sáez-Lara MJ, Robles-Sanchez C, Ruiz-Ojeda FJ, Plaza-Diaz J, Gil A. Effects of Probiotics and Synbiotics on Obesity, Insulin Resistance Syndrome, Type 2 Diabetes and Non-Alcoholic Fatty Liver Disease: A Review of Human Clinical Trials. *Int J Mol Sci.* 2016;17(6). 10.3390/ijms17060928

92. Rossi RE, Whyand T, Murray CD, Hamilton MI, Conte D, Caplin ME. The role of dietary supplements in inflammatory bowel disease: a systematic review. *Eur J Gastroenterol Hepatol.* 2016;28(12):1357-1364. 10.1097/meg.0000000000000728

93. Ried K. Garlic Lowers Blood Pressure in Hypertensive Individuals, Regulates Serum Cholesterol, and Stimulates Immunity: An Updated Meta-analysis and Review. *J Nutr.* 2016;146(2):389s-396s. 10.3945/jn.114.202192

94. Miller H, Ferris R, Phelps BR. The effect of probiotics on CD4 counts among people living with HIV: a systematic review. *Benef Microbes.* 2016;7(3):345-351. 10.3920/bm2015.0163

95. Jin X, Ruiz Beguerie J, Sze DM, Chan GC. Ganoderma lucidum (Reishi mushroom) for cancer treatment. *Cochrane Database Syst Rev.* 2016;4(4):Cd007731. 10.1002/14651858.CD007731.pub3

96. Güvenç IA, Muluk NB, Mutlu F, et al. Do probiotics have a role in the treatment of allergic rhinitis? A comprehensive systematic review and meta-analysis. *Am J Rhinol Allergy.* 2016;30(5):157-175. 10.2500/ajra.2016.30.4354

97. Grobler L, Nagpal S, Sudarsanam TD, Sinclair D. Nutritional supplements for people being treated for active tuberculosis. *Cochrane Database Syst Rev.* 2016;2016(6):Cd006086. 10.1002/14651858.CD006086.pub4

98. Carter GM, Esmaeili A, Shah H, et al. Probiotics in Human Immunodeficiency Virus Infection: A Systematic Review and Evidence Synthesis of Benefits and Risks. *Open Forum Infect Dis.* 2016;3(4):ofw164. 10.1093/ofid/ofw164

99. Zajac AE, Adams AS, Turner JH. A systematic review and meta-analysis of probiotics for the treatment of allergic rhinitis. *Int Forum Allergy Rhinol.* 2015;5(6):524-532. 10.1002/alr.21492

100. Singh M, Das RR. WITHDRAWN: Zinc for the common cold. *Cochrane Database Syst Rev.* 2015;2015(4):Cd001364. 10.1002/14651858.CD001364.pub5

101. Saez-Lara MJ, Gomez-Llorente C, Plaza-Diaz J, Gil A. The role of probiotic lactic acid bacteria and bifidobacteria in the prevention and treatment of inflammatory bowel disease and other related diseases: a systematic review of randomized human clinical trials. *Biomed Res Int.* 2015;2015:505878. 10.1155/2015/505878

102. Peng Y, Li A, Yu L, Qin G. The role of probiotics in prevention and treatment for patients with allergic rhinitis: A systematic review. *Am J Rhinol Allergy.* 2015;29(4):292-298. 10.2500/ajra.2015.29.4192

103. Hao Q, Dong BR, Wu T. Probiotics for preventing acute upper respiratory tract infections. *Cochrane Database Syst Rev.* 2015(2):Cd006895. 10.1002/14651858.CD006895.pub3

104. Giammanco M, Di Majo D, La Guardia M, et al. Vitamin D in cancer chemoprevention. *Pharm Biol.* 2015;53(10):1399-1434. 10.3109/13880209.2014.988274

105. Fritz H, Kennedy DA, Ishii M, et al. Polysaccharide K and Coriolus versicolor extracts for lung cancer: a systematic review. *Integr Cancer Ther.* 2015;14(3):201-211. 10.1177/1534735415572883

106. Allingstrup M, Afshari A. Selenium supplementation for critically ill adults. *Cochrane Database Syst Rev.* 2015;2015(7):Cd003703. 10.1002/14651858.CD003703.pub3
