# Supplementary material for: Select Dietary Supplement Ingredients for Preserving and Protecting the Immune System in Healthy Individuals: A Systematic Review
Source: Nutrients. 2022 Nov 1;14(21):4604. doi: 10.3390/nu14214604 (PMC9655067; doi:10.3390/nu14214604)
Supplement: Supplementary file 1 [file nutrients-14-04604-s001.zip › Supplementary File S2.pdf]

Supplemental File S2: Systematic review on select dietary supplements for immune health

Table of Contents

Methods 2

Table S1: Search strategy 2

Figure S1: PRISMA flow chart 4

Table S2: Types of outcome metrics used across studies 5

Results 7

Table S3: Characteristics of included studies 7

Table S4: Risk of bias across studies 17

Table S5: Outcomes – Statistics by population stressor 18

Table S6: Adverse event information 42

References cited 59

Methods

Table S1: Search strategy

Search Date: 2021-10-07

Database searched	Search string executed	Limits applied	Yielded # citations
**PUBMED**	*(Echinacea or elderberry or garlic or goji berry or "goji juice" or goldenseal or hydrastis or mangosteen or garcinia mangostana or melatonin or mushroom or agaricales or noni or morinda or probiotic or prebiotic or slippery elm or ulmus or vitamin a[Title/Abstract] or vitamin b[Title/Abstract] or vitamin c[Title/Abstract] or vitamin d[Title/Abstract] or zinc[Title/Abstract] or ginger or turmeric or curcuma or licorice or glycyrrhiza or selenium[Title/Abstract] or vitamin k[Title/Abstract] or astragalus or astragalus plant or calcium[Title/Abstract] or holy basil or ocimum sanctum or magnesium[Title/Abstract] or "rose hip" or rosehip or "Rosa"[Mesh] or vitamin e[Title/Abstract] or adaptogen) and (immune system or immune system phenomena or immunity or "immune function" or immun* or respiratory tract infections or "Infections/prevention and control"[Mesh])*	RCT, Humans, 2001 to present	3,146
**CINAHL + PsycInfo**	*(Echinacea or elderberry or garlic or "goji berry" or "goji juice" or goldenseal or "hydrastis" or "mangosteen" or "garcinia mangostana" or melatonin or (MH "Mushroom+") or "agaricales" or noni or "morinda" or probiotics or prebiotics or slippery elm or "ulmus" or (TI "vitamin a" OR AB "vitamin a") or (TI "vitamin b" OR AB "vitamin b") or (TI "vitamin c" OR AB "vitamin c") or (TI "vitamin d" OR AB "vitamin d") or (TI zinc OR AB zinc) or ginger or turmeric or curcumin or licorice or "glycyrrhiza" or (TI selenium OR AB selenium) or (TI "vitamin k" OR AB "vitamin k") or astragalus or "astragalus plant" or (TI calcium or AB calcium) or "holy basil" or "ocimum sanctum" or (TI magnesium or AB magnesium) or rosehip or "rose hip" or (TI "vitamin e" OR AB "vitamin e") or adaptogen) AND (immun* or "immune function" or immunity or "immune system phenomena" or (MH "Immune System+") or (MH "Immunity+") or (MH "Respiratory Tract Infections+") OR (MH "Infection+/PC"))*	Humans, Journal Article, RCTs, 2001-2021	480
**EMBASE**	*('echinacea'/exp OR 'echinacea' OR 'sambucus'/exp OR 'sambucus' OR 'elderberry'/exp OR 'elderberry' OR 'sambucus flower'/exp OR 'sambucus flower' OR 'allium sativum'/exp OR 'allium sativum' OR 'carisano'/exp OR 'carisano' OR 'garlic'/exp OR 'garlic' OR 'garlic powder'/exp OR 'garlic powder' OR 'goji berry'/exp OR 'goji berry' OR 'goji juice' OR 'hydrastis'/exp OR 'hydrastis' OR 'hydrastis canadensis'/exp OR 'hydrastis canadensis' OR 'goldenseal'/exp OR 'goldenseal' OR 'yellow root'/exp OR 'yellow root' OR 'goldenseal extract'/exp OR 'goldenseal extract' OR 'mangosteen'/exp OR 'mangosteen' OR 'mangosteen extract'/exp OR 'mangosteen extract' OR 'garcinia mangostana'/exp OR 'garcinia mangostana' OR 'garcinia mangostana extract'/exp OR 'garcinia mangostana extract' OR 'melatonin'/exp OR 'melatonin' OR 'mush-room'/exp OR 'mush-room' OR 'mushroom'/exp OR 'mushroom' OR 'agaricaceae'/exp OR 'agaricaceae' OR 'agaricales'/exp OR 'agaricales' OR 'indian mulberry'/exp OR 'indian mulberry' OR 'morinda citrifolia'/exp OR 'morinda citrifolia' OR 'noni'/exp OR 'noni' OR 'probiotic'/exp OR 'probiotic' OR 'probiotic agent'/exp OR 'probiotic agent' OR 'probiotics'/exp OR 'probiotics' OR 'prebiotic'/exp OR 'prebiotic' OR 'prebiotic agent'/exp OR 'prebiotic agent' OR 'prebiotics'/exp OR 'prebiotics' OR 'slippery elm' OR 'ulmus'/exp OR 'ulmus' OR 'elm'/exp OR 'elm' OR 'elm tree'/exp OR 'elm tree' OR 'vitamin a':ti,ab OR 'vitamin b':ti,ab OR 'vitamin c':ti,ab OR 'vitamin d':ti,ab OR zinc:ti,ab OR 'zingiber officinale'/exp OR 'zingiber officinale' OR 'zingiberis rhizoma'/exp OR 'zingiberis rhizoma' OR 'ginger'/exp OR 'ginger' OR 'zinziber officinale'/exp OR 'zinziber officinale' OR 'ginger extract'/exp OR 'ginger extract' OR 'zingiber officinale extract'/exp OR 'zingiber officinale extract' OR 'zintona'/exp OR 'zintona' OR 'turmeric'/exp OR 'turmeric' OR 'turmeric extract'/exp OR 'turmeric extract' OR 'curcuma'/exp OR 'curcuma' OR 'curcuma longa extract'/exp OR*	RCT, Humans, 2001 to present, Articles, Articles in Press	727

Database searched	Search string executed	Limits applied	Yielded # citations
	'curcuma longa extract' OR 'turmeric oleoresin'/exp OR 'turmeric oleoresin' OR 'glycyrrhiza glabra root'/exp OR 'glycyrrhiza glabra root' OR 'licorice'/exp OR 'licorice' OR 'licorice root'/exp OR 'licorice root' OR 'liquorice'/exp OR 'liquorice' OR 'radix glycorrhizae glabra'/exp OR 'radix glycorrhizae glabra' OR 'radix liquiritiae'/exp OR 'radix liquiritiae' OR 'sweet root'/exp OR 'sweet root' OR 'licorice extract'/exp OR 'licorice extract' OR selenium:ti,ab OR 'vitamin k':ti,ab OR 'astragalus extract'/exp OR 'astragalus extract' OR 'astragalus plant extract'/exp OR 'astragalus plant extract' OR 'astragalus (plant)'/exp OR 'astragalus (plant)' OR calcium:ti,ab OR 'ocimum sanctum'/exp OR 'ocimum sanctum' OR 'ocimum tenuiflorum'/exp OR 'ocimum tenuiflorum' OR 'holy basil'/exp OR 'holy basil' OR 'sacred basil'/exp OR 'sacred basil' OR 'tulasi'/exp OR 'tulasi' OR magnesium:ti,ab OR 'rosehip extract'/exp OR 'rosehip extract' OR 'rosehip' OR 'rosehip'/exp OR rosehip OR 'vitamin e':ti,ab OR 'adaptogen'/exp OR 'adaptogen') AND ('immun' OR 'immune system'/exp OR 'immune system' OR 'body defense'/exp OR 'body defense' OR 'immune defence'/exp OR 'immune defence' OR 'immune defense'/exp OR 'immune defense' OR 'immune enhancement'/exp OR 'immune enhancement' OR 'immune reaction'/exp OR 'immune reaction' OR 'immune resistance'/exp OR 'immune resistance' OR 'immune system phenomena'/exp OR 'immune system phenomena' OR 'immune system phenomenon'/exp OR 'immune system phenomenon' OR 'immune system process'/exp OR 'immune system process' OR 'immune system processes'/exp OR 'immune system processes' OR 'immunity'/exp OR 'immunity' OR 'immuno defence'/exp OR 'immuno defence' OR 'immuno resistance'/exp OR 'immuno resistance' OR 'immunodefense'/exp OR 'immunodefense' OR 'immunological reaction'/exp OR 'immunological reaction' OR 'immunoprotection'/exp OR 'immunoprotection' OR 'immunoresistance'/exp OR 'immunoresistance' OR 'local immunity'/exp OR 'local immunity' OR 'resistance, immune'/exp OR 'resistance, immune' OR 'immune function'/exp OR 'immune function' OR 'acute respiratory infection'/exp OR 'acute respiratory infection' OR 'acute respiratory tract infection'/exp OR 'acute respiratory tract infection' OR 'airway infection'/exp OR 'airway infection' OR 'bronchopulmonary infection'/exp OR 'bronchopulmonary infection' OR 'chronic respiratory tract infection'/exp OR 'chronic respiratory tract infection' OR 'infection, respiratory tract'/exp OR 'infection, respiratory tract' OR 'pulmonary tract infection'/exp OR 'pulmonary tract infection' OR 'respiration infection'/exp OR 'respiration infection' OR 'respiration tract infection'/exp OR 'respiration tract infection' OR 'respiratory infection'/exp OR 'respiratory infection' OR 'respiratory tract infection'/exp OR 'respiratory tract infection' OR 'respiratory tract infections'/exp OR 'respiratory tract infections')*		

Figure S1: PRISMA flow chart

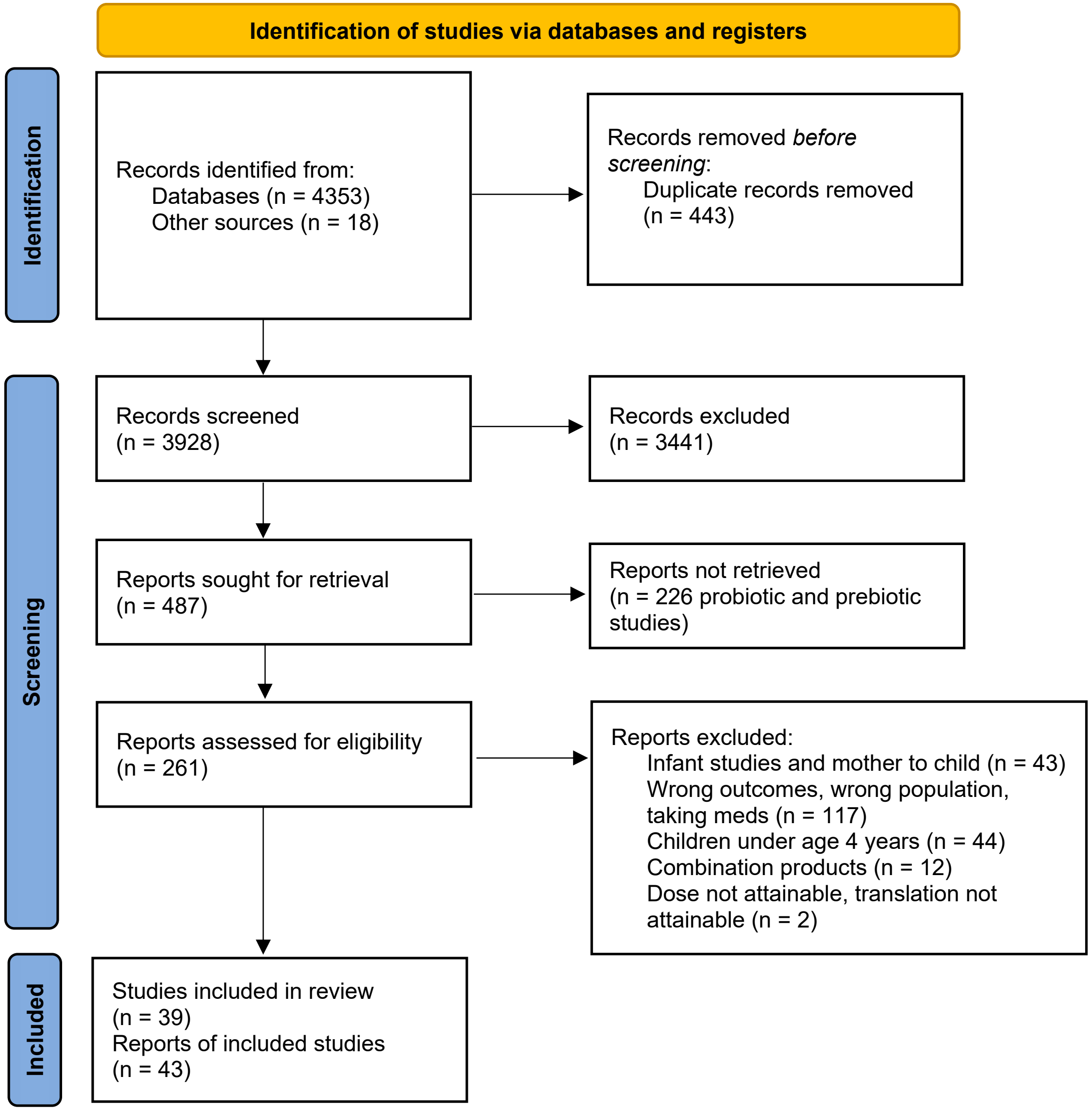

Table S2: Types of outcome metrics used across studies

	Echinacea	Elderberry	Garlic	Vitamin A	Vitamin C	Vitamin D	Vitamin E	Zinc
Persons that experience symptoms								
**Self-report questionnaires**	x		x	x	x	x	x	x
**Jackson scale**	x	x				x		
**Wisconsin Upper Respiratory Symptom Survey-11 (WURSS-11)**						x		
**Wisconsin Upper Respiratory Symptom Survey-21**		x			x	x		
**Wisconsin Upper Respiratory Symptom Survey 24**						x		
**Wisconsin Upper Respiratory Symptom Survey (WURSS-44)**	x							
Severity of symptoms								
**Self-report severity rating scales**	x	x			x	x		x
**Jackson scale**	x	x				x		
**Wisconsin Upper Respiratory Symptom Survey (WURSS-44)**	x							
**Total number of episodes**	x	x	x	x	x	x	x	x
**Avg. number of symptoms**	x		x			x	x	
Duration of symptoms								
**Average duration of sick days**			x	x		x		x
**Median number total days sick**	x						x	x
**Cumulative number of days sick**	x	x	x		x	x		
**Time to resolution**	x		x					
**Number of work/school days missed**	x		x			x		x
Adverse events								
**Self-report**	x	x	x		x	x	x	x
Laboratory evidence of infection								
**Nasal or throat specimens, PCR tests**	x					x	x	
Biomarkers								
**Y& T-cells**			x					x

	Echinacea	Elderberry	Garlic	Vitamin A	Vitamin C	Vitamin D	Vitamin E	Zinc
NK cells			x					
IL-8, IL-6	x					x		
secretory immunoglobulin A (SIgA)						x		
serum levels of neopterin and DHEA-S							x	
Changes in Serum levels				x		x		
Deficiency status: conjunctival impression cytology				x				
polymorphonuclear-leukocyte concentrations in nasal-lavage specimens	x							
Physical activity								
Godin-Leisure Time Exercise Questionnaire					x			
Restriction on activity self-report							x	
Decrease in activity illness (incidence and days)			x					
Physical severity (WURSS-21)						x		
Diet Quality								
Rapid Eating and Activity Assessment for Patients (REAPS) tool					x			
Food Frequency Questionnaire						x		
General well being								
SF-12 acute		x						
Perceived Stress Scale		x						
Wisconsin Upper Respiratory Symptom Surveys						x		

Results

Table S3: Characteristics of included studies

Author, Year (Quality)	Methods	Participants	Stressor	Interventions	Total amount per day; Treatment duration	Outcomes
**Echinacea studies (N=6)**						
**Jawad 2012[1] (Acceptable quality)**	randomized, double-blind, parallel, placebo-controlled clinical trial	755 healthy participants recruited via advertisements around a university campus, aged >18 years (M=23.6; 68.7%F in treatment group) that experience >=2 colds per year	Study conducted throughout the winter months; participant who experience >=2 colds per year	*E. purpurea* (**Echinaforce**) drops prepared by alcoholic (57.3% m/m) extraction from freshly harvested *E. purpurea* with a combination of 95% herba (DER = 1:12) and 5% roots (DER = 1:11); standardized to contain 5 mg/100 g of dodecatetraenoic acid isobutylamide; Placebo drops similar in shape, color, consistency, odor, flavor, and they contained the same amount of alcohol. 3 × 0.9 mL per day for illness prevention. This corresponded to 2400 mg of extract per day. During acute stages of colds, the participants were instructed to increase the dose to 5 × 0.9 mL per day; this totaled a daily dose of 4000 mg of extract.	2.7 mL per day (2400 mg Echinacea) (prevention); 4.5 mL per day (4000 mg Echinacea) at onset of any cold; 4 months	Adverse events, blood samples of physiological measures, incidence of cold, severity of symptoms, nasal secretions
**Ogal 2021[2] (Acceptable quality)**	randomized, controlled, blinded study carried out at 13 general practices and	203 healthy children aged 4-12 years (M=7.8 in treatment group; %F ND)	winter season; most children attending school	3 x 400 mg of *Echinacea purpurea* extract **(Echinaforce®, EFJ)** per day comprising 380 and 20 mg of ethanolic extract from freshly harvested above-ground plant parts	1200 mg Echinacea per day; 2 x two months separated by a	**Primary:** cumulative number of days with cold symptoms **Other**: incidence of RTI episodes, severity and duration of symptoms,

	pediatric clinics in Switzerland			and roots, respectively (drug to extraction solvent ratio of 1:12 and 1:11, extractant 65% EtOH (VIV)) , delivering a total daily dose of 1200 mg, which corresponds to 50% of the adult prevention dose; Control tablets contained 50 mg vitamin C (20 mg ascorbic acid and 36 mg calcium ascorbate) taken three times per day and the same amount of natural orange flavor like verum for masking. Both tablets looked, smelled and tasted similar.	one-week treatment break	nasopharyngeal secretion sample, pathogens, subjective judgement of efficacy, RTI complications and antibiotic prescriptions, adverse events
**O'Neil 2008[3] (Acceptable quality)**	randomized, double-blind, placebo-controlled clinical trial	90 healthy adults working in a university medical center aged 18-65 years (M=40; %F=ND)	exposure to cold/influenza during the winter months	2 x 3 capsules per day of either *E. purpurea*, 300 mg per capsule or parsley, 300 mg per capsule	1800 mg/day; 8 weeks	Days with symptoms, number of days missed from work and any medications used to treat symptoms, adverse events
**Sperber 2004[4] (Low quality)**	randomized, double-blind, placebo-controlled clinical trial	48 healthy adult volunteers aged 18–65 years (M=33; 54%F) with serum neutralizing antibody titers of 1:2 to Rhinovirus (RV) type 39	intranasal inoculation with RV-39 at day 7	3 x 2.5mL Echinacea per day of pressed juice of the above-ground plant parts of *E. purpurea* placed in a 22% alcohol base (**EchinaGuard**); matching placebo (not described)	7.5 mL/day; 14 days	**Primary:** occurrence and severity of symptoms **Secondary:** increase in RV-39 neutralizing antibody titer and/or recovery, adverse events
**Tiralongo 2012[5] (Acceptable quality)**	randomized, double-blind, placebo-controlled trial	175 volunteers who were traveling for a period of 1-5 weeks from Australia to America, Europe or Africa aged 18-65 years (M=43, 67%F)	Intercontinental air travel with a flying time of 15–25 hours and less than 12-hour stopovers	2 x 1 Echinacea tablet per day of (**MediHerb brand**) standardized to 4.4 mg alkylamides; containing 112.5 mg *Echinacea purpurea* 6 : 1 extract (equivalent to 675 mg dry root) and 150 mg *Echinacea angustifolia* 4 : 1 extract (equivalent to 600 mg dry root) or 2 tablets during stressful time of flying during 5 weeks travel; matching placebo tablets in size, excipients, and color	Approx. 63 days; 14 days before travel (baseline), <1 week after travel (return at approx. 5 weeks), and follow up 4 weeks after returning from travel	**Primary:** upper respiratory symptom-related quality of life; **Secondary:** occurrence and duration of jet lag, headache, sleep pattern, and herpes simplex sores, adverse events

**Turner 2005[6] (Acceptable quality)**	randomized, placebo-controlled, double-blind study	437 healthy young adult volunteers susceptible to rhinovirus type 39, as evidenced by a serum-neutralizing antibody titer of 1:4 or less (M=20.8, 60%F)	rhinovirus type 39 challenge after 7 days	*Echinacea angustifolia* root extracted with either supercritical carbon dioxide, 60% ethanol or 20% ethanol for 3 different preparations; placebo containing a mixture of alcoholic beverages, denatonium benzoate (250 ppm), and tap water (8 total groups total); given 3 X/day as a 1.5 ml tincture containing the equivalent of 300 mg of echinacea	4.5 mL per day (900 mg Echinacea); days -7 until virus challenge on day 0 and then until 5 days post challenge; 13 day duration; follow up 3 weeks later	**Primary:** symptom score **Secondary:** testing of infection for rhinovirus type 39 with nasal specimens, inflammation IL-8, polymorphonuclear leukocytes, incidence of clinical colds and individual symptoms, adverse events
**Elderberry studies (N=1)**						
**Tiralongo 2016[7] (High quality)**	randomized, double-blind placebo controlled clinical trial	312 healthy adults with a minimum age of 18 (M = 51, 66%F) who were traveling economy class to an overseas destination	Long-flight air travelers (minimum of a 7 hour flight, i.e., Australia-Asia, less than a 12 hour stopover and a minimum of 4 days stay at the destination)	2 x 300 mg capsules/day for 10 days and 3 x 300 mg capsules/day for 5 days. Proprietary membrane filtered elderberry (Sambucus nigra L., Haschberg variety, Steiermark region in Austria; **BerryPharma Brand)**; Each elderberry capsule contained 300 mg of elderberry extract (22% polyphenols, 15% anthocyanins, 150 mg of rice flour, and several mineral, trace elements and vitamins); placebo manufactured to match the elderberry capsules in size, excipients, and appearance.	600 mg/day for 10 days before travel and 900 mg/day for 5 days (1 day before leaving home until 4/5 days after arriving at the destination); total of 15-16 d	**Primary:** Total number of cold episode days<br><br>**Other:** Upper respiratory symptoms severity and duration, general wellbeing and quality of life, perceived stress
**Garlic studies (N=2; 3 publications)**						
**Josling 2001[8] (Acceptable quality)**	randomized, double-blind, placebo-controlled survey	146 healthy volunteers recruited through advertisements in two London daily newspapers (M=52.5; 58%F)	Study conducted between November and February, winter months	1 capsule per day with main meal (**Allimax**) containing 180 mg of stabilized allicin; placebo (ND)	180 mg/day; 12 week duration	number of colds experienced; duration of symptoms; days to recover; adverse events
**Nantz 2012[9], Percival 2016[10] (Low quality)**	randomized, double-blind, placebo-controlled clinical trial	120 healthy men and women aged 21-50 years (M=26; 56%F) with a body mass index between 18-30 kg/m2,	Study conducted from February through May, which is	2 capsules twice daily containing either Aged Garlic Extract (**AGE**) powder (4 capsules: 2.56 g per day); placebo	2.56 g/day; 90 days duration	**Primary:** ex vivo proliferation of γ&-T cell and NK cells **Secondary:** blood pressure measurements, incidence of

		recruited from the University of Florida campus and Gainesville Florida community	considered cold and flu season, and also included the end of the semester for students, subjecting them to the stressor of final exams	capsules (ND); **Wakunaga of America, Co. Ltd.**		illness, severity, number of missed days from school and work, number of doctor visits, decrease in activity, side effects, medications
**Vitamin A studies (N=2)**						
**Chen 2013[11] (Low quality)**	double-blind, randomized placebo-controlled trial	445 preschool children aged 3-6 years old, in Huayuan Town, Pixian County of Chengdu City, western China (Treatment group M=4.4; 46%F, Serum retinol status 1.15+-0.30 umol/L)	Peri-urban community in a kindergarten environment	200000 IU vitamin A capsule (as retinol) provided by **Sichuan Pearl Pharmaceutical Co., Ltd,** just one time initially; placebo received neither vitamin A nor ferrous sulfate (ND)	children monitored over a six month period	change in serum Hb and infectious illness
**Chowdhury 2002[12] (Low quality)**	randomized, double-blind design	1520 non-xerophthalmic children less than 10 years of age from three slums of Chandigarh with a high prevalence of Vitamin A deficiency (M=53 months in treatment group; 60% with sub-clinical vitamin A deficiency status)	environment with high prevalence of vitamin A deficiency	2 ml vitamin A (200000 IU) to children more than 12 months, 1 ml (100000 IU) to children age 6-12 months and 0.5 ml (50000 IU) to less than 6 month olds; placebo given in equal volume as arachis oil	once every 4-6 months during a 15 month trial period	incidence of and number of episodes of illness; deficiency status
**Vitamin C studies (N=3)**						
**Constantini 2011[13] (High quality)**	randomized, double-blind, placebo-controlled trial	39 competitive young swimmers aged 12-17, (M= 13.8±1.6; 43%F) in Jerusalem, Israel who trained at least 15h/wk.	throughout the winter months involving competitive young swimmers (December - February)	2 X 0.5 g vitamin C (ascorbic acid) prepared by **Teva Pharmaceutical Industries Ltd.**, Netanya, Israel; placebo pills identical in appearance	1 g/day; three winter months	incidence, episodes, duration and severity of symptoms
**Johnston 2014[14]**	randomized, double-blind,	30 healthy men, aged 18-35 years; BMI <34 kg/m2, recruited from a large campus	young men with marginal vitamin	2 capsules per day (morning and evening) of 500 mg vitamin C per	1000 mg/day; 8 weeks duration	incidence, severity and duration of symptoms, impact of cold on daily living,

**(Acceptable quality)**	placebo-controlled study	in the Southwestern US, with plasma vitamin C concentrations less than 45 umol/L (M=23; average Plasma vitamin C 30.2+-8.5 in treatment group)	C status during the peak of the cold season (January to April).	capsule, **Twinlab C-500 CAPS**); placebo containing white flour		physical activity levels, food frequency, diet quality, blood samples
**VanStraten 2002[15] (High quality)**	randomized, double-blind, placebo-controlled survey	168 healthy participants recruited through an editorial in consumer media from the UK (M=48 in the treatment group; 84% F)	throughout the winter months (November - February)	2 tablets of **Ester-C ascorbate** 500 mg, or a matched placebo, every day with their main meal	1000 mg per day/60 day duration	general well-being, number of colds, duration of symptoms, adverse events
**Vitamin D studies (N=18; 20 publications)**						
**Camargo, 2020[16], Scragg 2016[17] (High quality)**	randomized, double-blinded, placebo-controlled trial	5110 New Zealand adults participating in the VIDA study, aged 50-84 years, with a mean baseline blood 25-hydroxyvitamin D level of 63 (24) nmol/L; 25% were < 50 nmol/L (M=66; 42%F)	Older adults, some of which were considered insufficient	2 X 100,000 IU [2.5 mg] vitamin D3 as an initial dose or placebo soft-gel capsules, from **Tishcon Corp**. bolus, followed by a monthly 100,000 IU	daily dose approx. 3300 IU/day; 1.6 years	**Primary:** time to first ARI event<br>**Other:** Adverse events, vitamin D blood levels
**de Gruijl, 2012[18] (Low quality)**	randomized controlled trial	106 volunteers most of NW European descent, 18-30 years with a mean vitamin D level of 58-62 nmol/L (M=22; 92% F)	mid-winter 8-week period (January–March)	daily 1000 IU (25 µg) of vitamin D3 orally in gel capsules (**Solgar Vitamins**, Haarlem, The Netherlands); control group asked to "*go about their usual business*" without vitamin D supplementation	1000 IU/day; 8 weeks	vitamin D status and incidence of colds
**Dubnov-Raz, 2015[19] (Acceptable quality)**	randomized, placebo-controlled trial	55 adolescent competitive swimmers, aged 12-21 years, who train approximately 15 hrs/week from Israel with vitamin D "insufficiency" (average of 60 nmol/L) (M=15; 36% F)	throughout the winter months (Nov-Jan), and training approx. 15 hours/week	vitamin D3 (2,000 IU·d-1) in 10 liquid drops; placebo with identical taste and bottle packaging, supplied by the same manufacturer (**CTS Chemical Industries Ltd, Israel)**	2,000 IU/day; 12 weeks	**Primary:** number of URIs, duration and severity<br>**Other:** serum vitamin D concentrations
**Ganmaa, 2020[20] (Acceptable quality)**	phase 3, double-blind, randomized, placebo-controlled trial	8851 children attending one of 18 public schools in Ulaanbaatar, Mongolia, aged 6-13 years, with mean serum	school age children with vitamin D insufficiency and living in	one capsule per week containing either 14,000 IU (0.35 mg) of vitamin D3 or placebo	14,000 IU/week; 3 years	**Primary:** development of M. tuberculosis infection<br>**Secondary:** incidence of active tuberculosis and acute respiratory infection

		25(OH)D level of 30 nmol/L; (M=9.4; 49% F)	environments with prevalent TB			
**Goodall, 2014[21] (Acceptable quality)**	2 X 2 factorial randomized, double-blind, controlled trial	600 McMaster University students living with at least one student housemate, aged >17 years (baseline level ND) (Median age=19; 64% F)	peak rates of rhinovirus infection Sept - Oct when vitamin D levels have likely not yet declined as it does during autumn and winter months	one capsule per week containing either 10,000 IU of active vitamin D3 (from **Euro-Pharm International Canada Inc**.); identical placebo (calcium carbonate)	10,000 IU/week; 8 weeks	**Primary:** incidence of clinical URTI **Secondary:** laboratory confirmed illness, viral load, symptom duration and severity
**Harrison, 2021[22] (Acceptable quality)**	double-blind, randomized placebo-controlled trial	250 British Army recruits during basic military training aged 22+-7 years, only 25% of which were vitamin D sufficient.	during basic training, and throughout winter months (Jan-Feb)	one vitamin D3 capsule daily, analyzed as containing 1090 and 460 IU during the restoration and maintenance phases, respectively (**Pure Encapsulations, Sudbury, MA**); identical-looking cellulose placebo capsule daily (**Almac Group, County Armagh, UK**).	restorative phase 4 weeks, maintenance phase 8 weeks; 12 weeks total	prevalence, severity and duration of URTI, serum blood levels, salivary secretory immunoglobulin
**Hauger, 2019[23] Mortensen, 2016[24] (Acceptable quality)**	randomized, double-blind, placebo-controlled trial	130 children living in the capital area of Denmark (55°N), aged 4-8 years, (M=6.7; 53%F) with mean ± SD serum 25(OH)D of 56.7 ± 12.3 nmol/L	during winter months Oct–March	10 or 20 µg/day (400 or 800 IU) of vitamin D3; placebo (ND)	10 or 20 ug/day; 20 weeks	incidence of respiratory infection, biomarkers
**Jorde 2016[25] (Acceptable quality)**	randomized controlled trial	511 subjects with prediabetes from North Norway with baseline serum 25(OH)D levels 59.9 ± 21.9 nmol/L in the vitamin D group and 61.1 ± 21.2 nmol/L in the placebo group (M=62; 39% F)	subjects had pre-diabetes	one capsule vitamin D (cholecalciferol 20,000 IU (**Dekristol; Mibe, Jena, Germany))** per week or an identical looking placebo capsule containing arachis oil (Hasco-Lek, Wroclaw, Poland)	20,000 IU/week; 5 years	occurrence and number of infections
**Jung, 2018[26] (Acceptable quality)**	randomized, double-blinded, and placebo-controlled	26 collegiate male taekwondo athletes in South Korea, aged 19–22 years with vitamin D insufficiency	training during the winter months, vitamin D insufficient	daily vitamin D3 (5000 IU) or a visually identical placebo capsule **(Bio-Tech Pharmacal Inc.**, Fayettville, AR, USA)	5000 IU/day; 4 weeks	symptoms of URTI, Quality of life, SIgA and lactoferrin, serum blood levels

		(<50 nmol/L) (M=31.3 1.39 nmol/L) who trained 5X/wk, at least 3 hrs/d (M=19.9)				
**Laaksi, 2010[27] (Acceptable quality)**	blinded, placebo-controlled randomized trial	164 voluntary young Finnish men (18–28 years of age) undergoing compulsory periodic military training as conscripts in an infantry unit in the Finnish Defense Forces with mean serum 25(OH)D concentrations of 78.7 14.9nmol/L (T) and 74.4 20.8 nmol/L ©	during periodic military training Oct - March	400 IU (10 mg) vitamin D3 **(Minisun**; Verman) daily; placebo **(Pharmia**; a capsule identical in size and form to the active preparation)	400 IU/d; 6 months	**Primary:** number of days absent from duty **Secondary:** symptoms of ARTI and hospitalizations
**Li-Ng 2009[28] (Acceptable quality)**	prospective, randomized, double-blind, placebo-controlled trial	162 healthy adults living in Long Island, NY with serum vitamin D levels of (64.3+-25.4 nmol/l in the vitamin D group; 63.0+-25.8 nmol/l in the placebo group (M=59 in treatment arm; 80% F)	throughout the winter months (Dec - June months)	50 ug/d (2000 IU) vitamin D3 or matching placebo	2000 IU/day; 12 weeks	incidence of URI, severity and duration of symptoms
**Loeb 2019[29] (High quality)**	placebo-blinded randomized controlled trial	1300 healthy children and adolescents living in Vietnam, between the ages of 3 and 17 years, with mean baseline serum 25-hydroxyvitamin D levels 65.7 nmol/L and 65.2 nmol/L in the intervention and placebo groups (M=8.5; 52%F)	children living in Vietnam where there is a high prevalence of deficiency and less vaccinated	7 drops (0.028 mL per drop) of vitamin D drops (**Ddrops** Company, Woodbridge, ON) (14 000 IU/wk of vitamin D) weekly ; placebo received 7 drops (0.028 mL per drop) of placebo liquid, fractionated coconut oil, identical in appearance and taste to the vitamin D liquid	14,000 IU/week; 8 months and then 4 month surveilance period	**Primary:** reverse transcriptase (RT)-PCR–confirmed influenza infection, multiplex PCR–confirmed non-influenza respiratory viruses **Other:** a course of antibiotics, pharmacy visit, over the counter medication for respiratory symptoms, private medical clinic visits, and hospital or medical center visits

**Murdoch, 2012**[30] **(High quality)**	randomized, double-blind, placebo-controlled trial	322 staff or students of the Canterbury District Health Board, the regional publicly funded health care organization, or the University of Otago, Christchurch, New Zealand who were aged 18 years or older (M=47; 75%F) with Serum 25-hydroxyvitamin D level mean 29 ng/mL (72.5 nmol/L)	Staff or students to include winter months	oral vitamin D3, 200 000 IU, then 200 000 IU 1 month later, then 100 000 IU monthly thereafter; matching inactive tablets administered in a dosing regimen identical to the active treatment. Both sourced from **Tishcon Corp**	200,000 IU tapering down to 100,000 IU monthly; 18 months	**Primary:** number of URTI episodes **Secondary:** number of days of missed work as a result of URTI episodes, duration of URTI episodes, severity of URTI episodes, and detection of respiratory viruses in nasopharyngeal samples
**Rees 2013**[31] **(Acceptable quality)**	randomized, double-blind, placebo-controlled multicenter trial	759 participants, aged 45-75 years, from 11 clinical centers who had at least 1 colorectal ademona recently removed and none remaining with no familial colorectal cancer syndromes, in good health, serum vitamin D levels >12ng.mL (30 nmol/L), mean in treated group 33 ng/mL (82.5 nmol/L) (M=61; 42% F)	throughout the winter months	1000 IU per day of Vitamin D3; identical looking placebo pills	1000 IU/day; approximately 1 year	URTI, cold and flu episodes and duration of illness
**Shimizu, 2018**[32] **(Acceptable quality)**	prospective, randomized, double-blind, placebo-controlled study	252 healthy Japanese participants aged 45-74 years, with serum 25-hydroxyvitamin D level of 30 ng/mL (=75nmol/L) or less (M=53; 69% F)	throughout winter - spring (Jan - May)	hard capsule formulation containing 10 µg (400 IU) of 25-hydroxivitamin D3 (25OHD); placebo was prepared by replacing 25OHD with crystalline cellulose so that it would not be distinguished from the trial supplement by the color. 25OHD was provided for free by **DSM Nutritional Products, Ltd**. (Heerlen, Netherlands).	10 ug/day; 16 weeks	**Primary:** incidence of URTI, **Secondary:** physical severity, quality of life, duration of illness, adverse events
**Simpson, 2015**[33] **(High quality)**	double-blind, randomized placebo-controlled trial	34 Australian students and staff and their friends/family of Menzies Research Institute Tasmania and the University School of Medicine, the Royal	throughout the winter months (May-Oct)	20,000 IU/week cholecalciferol supplementation (roughly 3000 IU/day); placebo capsules identical in appearance; **Dartnells Pharmacy**, Victoria, Australia	average of 3000 IU/day; 16.4 weeks	occurrence and magnitude of infection symptoms, severity and duration, adverse events, serum samples

		Hobart Hospital and TasTAFE Campbell Street campus, aged 18-60 with a mean 25(OH)D at study entry of 67.9 nmol/L (M=32; 59% F)				
**Urashima 2010[34] (Acceptable quality)**	multicenter, randomized, double-blind, placebo-controlled, parallel-group trial	430 schoolchildren aged 6-15 years in Japan (M=10; 44% F)	throughout four months of winter (Dec-March)	3 X 200 IU vitamin D3 tablets twice daily; placebo tablets identical in appearance (**Zenyaku Co, Ltd** (Otsuka, Bunkyo-ku, Tokyo, Japan)	1200 IU/day; 4 months	incidence of influenza A (or B) (diagnosed with antigen testing), adverse events
**Urashima 2014[35] (High quality)**	randomized, double-blind, placebo-controlled parallel-group trial	247 high school students, aged 15-18 years, at Seisoku High School in Tokyo, Japan (freshman, sophomores and seniors; 34% F)	throughout an influenza pandemic (Oct-Dec)	5 capsules daily of 400 IU vitamin D; placebo capsules identical in appearance **Zenyaku Co., Ltd**. (Otsuka, Bunkyo-ku, Tokyo, Japan)	2000 IU/day; 2 months	Occurrence of influenza A positive test, adverse events, school absences, influenza symptoms
**Vitamin E studies (N=1; 2 publications)**						
**Graat 2002[36], Van Amsterdam 2005[37] (Acceptable quality)**	randomized, double-blind, placebo-controlled, 2X2 factorial trial	652 noninstitutionalized individuals aged 60 years or older from Netherlands with overall healthy micronutrient status (treatment group M= 73.1; 51%F)	seniors taking supplements for a long duration to include at least three winter months (October - February)	2 X 200 mg/dL of alpha-tocopheryl acetate per day with dinner; placebo capsules contained soybean oil	400 mg/dL per day; 15 months	**Primary:** incidence, severity of acute respiratory tract infections,<br>**Other:** duration, serology tests, blood samples<br>Post-hoc (sub-sample): serum levels of neopterin and DHEA-S
**Zinc studies (N=6)**						
**Kurugol 2006[38] (High quality)**	randomized, double-blind, placebo-controlled, prospective study	200 healthy children, aged 2-10 years old, from Ege University Nursery and Primary School (treatment group M=5.5; 51%F; avg zinc level 84.2 (72-116 ug/dl)	attending nursery and primary school during the winter months	zinc sulphate (15 mg of zinc) syrup supplied by **Berko Ilac¸ Company, Turkey**, once per day; placebo syrup identical in appearance, texture and flavoring content. Dose was increased 2 X/day if at least two symptoms occurred until cold symptoms resolved	15 mg/day; 7 month duration; 30 mg/day if symptoms occurred, up to 10 d duration	**Primary:** number of colds per study child<br>**Secondary:** duration and severity of symptoms, adverse effects, throat and nasal swabs for detection of virus
**Prasad 2007[39] (Low quality)**	randomized, placebo-controlled trial	50 healthy elderly adults of both sexes aged 55–87 years, from a senior citizen center	seniors some of which were zinc-deficient and	3 X 1 capsule of zinc gluconate (15 mg elemental zinc) - 1 in the morning and 2 before going to bed each day	45 mg/day elemental zinc; 12 months	**Primary**: incidence of infection

		(treatment group M=65; 67% F) with plasma zinc levels of 94.3+- 11.4 ug/dL	oxidatively stressed moreso compared to younger adults	supplied by **Labcatal Laboratories, Paris, France**; placebo capsules (ND)		**Secondary:** types of infection and laboratory variables such as plasma zinc, percent of cells producing cytokines and generation of cytokines and plasma oxidative stress markers
**Rerksuppaphol 2013[40] (Acceptable quality)**	double-blind randomized controlled trial	100 children , aged 8-13 years, attending a public school in Ongkharuck district, Thailand (treatment group M=10; 44%F)	children attending public school during the winter months (November - January)	15 mg chelated zinc (zinc bis-glycinate - **Qualimed,** Thailand) tablet once daily; placebo tablet prepared by same company identical in color, size and taste	15 mg/day; 3 months	**Primary:** occurrence of any symptom<br>**Secondary:** occurrence of vomiting, diarrhea, use of antibiotics, school absence, duration of symptoms
**Richard 2006[41] (Acceptable quality)**	community-based, randomized, double-blind, placebo-controlled trial	855 children aged 0.5–15 years, from a rural village in the Amazon, some of whom were anemic, stunted or zinc deficient at baseline (approximately 70% aged 5 and older; 54.7% F in zinc group; 10.80 (2.99) umol/L mean plasma zinc level in zinc group)	rural Peruvian village of Santa Clara, which is located in the Amazon region near the city of Iquitos, Peru, an area with mesoendemic seasonal (peak April–June) malaria	20 mg zinc (zinc sulfate) given as 2.5 mL of a red strawberry-flavored syrup made in Lima, Peru by a **Peruvian pharmaceutical Lab** (Instituto Quimioterapico, Peru); placebo similar in appearance and taste bottled in similar containers	20 mg per day; 7 months (Feb-September)	blood tests, incidence of infection (ALRI)
**Vakili 2009[42] (Low quality)**	randomized, double-blind, placebo-controlled, community-based intervention trial	200 school children aged 6-10 years old, (M=7.8; 50%F) living in a low socioeconomic suburb of Mashhad (Altimor), north-east Iran	conducted during cold season (November - March) in Altimor suburb of Mashhad, northeast Iran involving school-aged children	10 mg elemental zinc tablet; placebo (ND)	10 mg per day (6 days a week); 5 month duration	occurrence of the common cold; need for antibiotic; days missing school
**Veverka 2009[43] (Acceptable quality)**	randomized, double blind, placebo-controlled design	40 male and US Air Force female cadets (M=18.5; 20% F) with mean zinc levels in	cadets during the cold and flu season (Oct-April)	15 mg capsule of zinc supplied through an "FDA licensed clinical specialty pharmacy (FL, USA); placebo capsule	15 mg per day; 7 months	**Primary:** URI infection rate (self-reported and physician diagnosed)

		treatment group 94.9+- 17.3 ug/dL	exposed to a number of stressful situations (basic training and academic life)	same type as used for zinc but filled with cornstarch or gelatin		**Other:** laboratory analysis, plasma biomarkers, severity of symptoms

Table S4: Risk of bias across studies

Percentage of studies adequately addressing criteria across each ingredient.

Criteria evaluated[a]	Echinacea (N=6)	Elderberry (N=1)	Garlic (N=2)	Vitamin A (N=2)	Vitamin C (N=3)	Vitamin D (N=18)	Vitamin E (N=1)	Zinc (N=6)
Addresses an appropriate and clearly focused question	100	100	100	100	100	100	100	100
Assignment of subjects to treatment groups is randomized	83	100	50	100	100	100	100	67
Adequate concealment method is used	50	100	50	0	67	56	100	50
Subjects and investigators kept blind about treatment allocation	83	100	0	0	100	94	100	17
Treatment and control groups similar at start of the trial	100	100	100	100	100	88	100	83
Only difference between groups is the treatment under investigation	100	100	100	50	100	83	100	100
Relevant outcomes measured in a standard, valid and reliable way	67	0	100	0	100	83	0	100
Percentage of individuals dropped out before study completed[b]	83	100	100	100	100	72	100	*1 ND*
Subjects analyzed in the groups to which they were randomly allocated	67	100	0	0	33	33	100	33
Where study is carried out at more than one site, results are comparable	NA	NA	NA	NA	NA	Yes	NA	NA
Number of low-quality studies[c]	1 of 6	0 of 1	1 of 2	2 of 2	0 of 3	1 of 18	0 of 1	3 of 6
**Overall judgement of risk of bias across studies[d]**	**Not serious**	**Not serious**	**Serious**	**Serious**	**Not serious**	**Not serious**	**Not serious**	**Serious**

NA = not applicable, ND = not described
aThe SIGN 50 Checklist for randomized clinical trials was used to evaluate risk of bias. Studies were marked as *yes* for adequately addressing criteria, *no* if criteria was not addressed, or *can't say* when there was unclear/ insufficient information to make a determination. Percentages of criteria marked with *yes* are displayed in the table.
bPercentage of dropouts was unable to be calculated if some studies did not report dropouts; these studies are marked as ND followed by the number of studies that did not report dropout rates. Downgraded if dropouts were above 20%.
cEach study was scored overall as low, acceptable, or high quality, as noted in Table S3.
dGRADE overall judgement for risk of bias

Table S5: Outcomes – Statistics by population stressor

Study	Incidence of symptoms (# persons)	Severity of symptoms	Duration of symptoms	Adverse events	Laboratory evidence of infection	Biomarkers	Other resilience-type outcomes
**Echinacea study outcomes**							
Adult participants inoculated with RV Type 39 - Short term duration up to 14 days<br>**N=2 studies (n=485 participants) of different Echinacea formulations and different types of administration and amounts per day.**							
Sperber 2004[4]<br>**7.5 mL per day *E. purpurea* EchinaGuard for 14 days/inoculated with virus at day 7**	T=14/24; C=18/22<br>RR 0.713 (95% CI, 0.482, 1.054; P=0.09); 29% less risk of developing symptoms when taking echinacea	T=9.34+-9.43;<br>C=12.17+-9.56<br>SMD -0.298 (95% CI, -0.880, 0.284; P=0.315)		T=2/24; C=4/22<br>RD -0.098 (-0.294, 0.097; P=0.323)	T=22/24; C=21/22<br>RR 0.960 (95% CI, 0.826, 1.117; P=0.60)		

Turner 2005[6] ***Echinacea angustifolia* root 60% extract 4.5 mL or 900 mg per day for 13 days)/inoculated with virus at day 7**	T=24/52; C=58/103 RR 0.820 (95% CI, 0.584, 1.151; p=0.251); 18% less risk of developing symptoms when taking echinacea	T=13.21+-13.77; C=15.05+- 14.51 SMD -0.129 (95% CI, -0.463, 0.205; P=0.449)		T=15/315; C=4/104 RD 0.009 (95% CI, -0.035, 0.053; P=0.682)	T=42/52; C=88/103 RR 0.945 (95% CI, 0.810, 1.104; P=0.477)	*"Neither …had a significant effect on either the inter leukin-8 response or polymorphonu clearleukocyte response to rhinovirus infection…ther e was a significant correlation between in terleukin-8 and polymorphonu clearleukocyte responses and symptom severity."* Data not shown	

Study	Incidence of symptoms (# persons)	Severity of symptoms	Duration of symptoms	Adverse events	Laboratory evidence of infection	Biomarkers	Other resilience-type outcomes
Turner 2005[6] ***Echinacea angustifolia* root 20% extract 4.5 mL or 900 mg per day for 13 days)/inoculated with virus at day 7**	T=24/52; C=58/103 RR 0.820 (95% CI, 0.584, 1.151; p=0.251); 18% less risk when taking echinacea	T=12.06+-12.55; C=15.05+-14.51 SMD -0.215 (95% CI, -0.550, 0.119; P=0.207)			T=48/52; C=88/103 RR 1.080 (95% CI, 0.966, 1.208; P=0.175)		
Turner 2005[6] ***Echinacea angustifolia* root CO2 extract 4.5 mL or 900 mg per day for 13 days)/inoculated with virus at day 7**	T=25/45; C=58/103 RR 0.987 (95% CI, 0.722, 1.348; P=0.932); 1.3% less risk when taking echinacea	T=15.45+-15.70; C=15.05+-14.51 SMD 0.03 (95% CI, -0.32, 0.38; P=0.880)			T=40/45; C=88/103 RR 1.040 (95% CI, 0.913, 1.185; P=0.552)		
Adult participants with equitable exposure to cold/influenza during the winter months – up to 4 months duration **N=2 studies (n=845 participants) *E. purpurea capsules vs drops at different amounts***							

Jawad 2012[1]<br>***E. purpurea* (Echinaforce) drops 2.7 mL per day (2400 mg Echinacea) (prevention); 4.5 mL per day (4000 mg Echinacea) at onset of any cold; 4 months**	NR per person	NR per person<br><br>Episodes: T:149; C: 188	*"The placebo group had ..a collective duration of 850 episode days;..the Echinaforce group .. a collective duration of 672 episode days (ITT population). The difference of cumulated events (episodes and episode days) between the treatment groups each of 26% reached statistical significance for episode days (P < 0.05, chi-square test)."*	T=177/355; C=172/362<br>RD 0.023 (95% CI, -0.050, 0.097; P=0.530)	T=54/86; C=74/115<br>RR 0.976 (95% CI, 0.789, 1.206; P=0.821)		

Study	Incidence of symptoms (# persons)	Severity of symptoms	Duration of symptoms	Adverse events	Laboratory evidence of infection	Biomarkers	Other resilience-type outcomes
O'Neill 2008[3] ***E purpurea*** **1800 mg capsules per day for 8 weeks**		*"..Approximately 40% of the participants reported having symptoms most weeks, with no differences between the 2 groups… placebo group consistently reported symptoms during any given week more than the echinacea group..However, none of these differences were statistically significant."*	*"Median number sick days (the sum of the most prevalent symptom each week) 9 days in echinacea vs 14 days in control; total of 56 days; (z = 0.42; P= 0.67)."*	T=2/28; C=2/30<br>RD 0.005 (95% CI, -0.126, 0.135; P=0.943)			
Adult participants during stressful air travel - approximately 7 weeks duration<br>**N=1 study (n=175 participants) concluding, *"Although respiratory symptoms for both groups increased significantly during travel periods associated with long-haul flights, the increase of these symptoms for the Echinacea group was significantly lower than for the placebo group."***							

Study	Incidence of symptoms (# persons)	Severity of symptoms	Duration of symptoms	Adverse events	Laboratory evidence of infection	Biomarkers	Other resilience-type outcomes
Tiralongo 2012[5]<br>**2 Echinacea tablets of** mediHerb **brand standardised to 4.4mg alkylamides; containing 112.5mg *Echinacea purpurea* 6 : 1 extract (equivalent to 675 mg dry root) and 150 mg *Echinacea angustifolia* 4 : 1 extract (equivalent to 600 mg dry root) or 2 tablets during stressful time of flying during 5 weeks travel; started 14 days prior to travel**	T=31/72; C=43/76<br>RR 0.761 (95% CI, 0.547, 1.059; P=0.105); 24% less risk when taking echinacea during travel;<br><br>T=18/72; C=29/75<br>RR 0.647 (95% CI, 0.396, 1.057; P=0.082) 35% less risk when taking Echinacea four weeks after travel	*"during travel (measured retrospectively at return), the placebo group had significantly higher WURSS-44 scores on average compared to the Echinacea group (26 versus 13, P = 0.05)."*		T=4/85; C=1/85<br>RD 0.035 (95% CI, -0.015, 0.086; P=0.171)			
Children during the winter season – approximately 4 months duration<br>***<u>N=1 study (n=203 children)</u> Echinacea purpurea concluding, "Our results support the use of Echinacea for the prevention of RTIs…"***							
Ogal 2021[2]<br>***Echinacea purpurea* extract (Echinaforce®, EFJ) 1200 mg per day for 4 months – one week treatment break**	T=40/103; C=54/98<br>RR 0.705 (95% CI, 0.522, 0.952; P=0.023); 29.5% less risk when taking echinacea	T=35.5+-28.76;<br>C=53.5+-42.8<br>SMD -0.496 (95% CI, -0.777, -0.215; P=0.001)<br><br>Episodes T: 61; C=86	T=5.7+- 2.74; C:7.1+-3.38<br><br>MD -1.4 (95% CI, -2.427, -0.373; P=0.008)<br><br>*"The cumulative number of cold days was 429 days for EFJ (N = 103 subjects) and 602 days for C (N = 98 subjects)*<br>*47% more symptom days for C and a statistically significant*	T=51/103; C=45/98<br>RD 0.036 (95% CI, -0.102, 0.174; P=0.610)	T=57/103; C=72/98<br>RR 0.753 (95% CI, 0.610, 0.930; P=0.008)		

Study	Incidence of symptoms (# persons)	Severity of symptoms	Duration of symptoms	Adverse events	Laboratory evidence of infection	Biomarkers	Other resilience-type outcomes
			superiority for EFJ (ITT, p < 0.0001, Chi-square test).”				
**Elderberry study outcomes**							
Adults during stressful air travel – up to 16 days<br>***N=1 study (n=312 participants), concluding, “These data suggest a significant reduction of cold duration and severity in air travelers. More research is warranted to confirm this effect and to evaluate elderberry's physical and mental health benefits”***							
Tiralongo 2016[7]<br>**600-900 mg/d elderberry (Sambucus nigra L., Haschberg variety, Steiermark region in Austria; BerryPharma Brand); 15-16 d**	T=12/158; C=17/154<br>RR 0.688 (95% CI, 0.340, 1.392; P=0.298)	*“the symptom score in the placebo group over these days was 583, whereas in the elderberry group it was 247”*<br><br>*“decreased the symptom load (mean 21 vs 34)” SD not provided*	Number of episode days: T=57; C=117 (P=0.05)<br><br>*“On average, a 2 day shorter duration of the cold (4.75 vs 6.88 d)”*	T=2/158; C=3/154<br>RD -0.007 (95% CI, -0.035, 0.021; P=0.632)			*SF-12 physical component “for the actual travel and arriving in a potential different climate and time zone… physical health declined significantly in placebo (P=0.005) but was stable in the elderberry group (P=0.9)”*<br><br>*SF-12 mental component: “non-significant trend for the*

Study	Incidence of symptoms (# persons)	Severity of symptoms	Duration of symptoms	Adverse events	Laboratory evidence of infection	Biomarkers	Other resilience-type outcomes
							active group to have higher average mental health score than placebo (P=0.07)"
**Garlic study outcomes**							
Healthy men and women throughout the cold and flu season - approximately 3 months duration **N=2 studies (n=266 participants) taking different garlic products in varying amounts**							
Josling 2001[8] **180 mg/day of stabilized allicin Allimax; 12 week duration**	NR per person	*"Number of episodes: T=24; C=65; P<0.001"*	*"Duration of symptoms – avg. days T=1.52; C=5.01 P<0.001 (No SD reported)* *Days to recovery: T=4.63; C=5.63"*	T=5/73; C=2/73 RD 0.041 (95% CI, -0.028, 0.110; P=0.243)			
Nantz 2012[9] **2.56 g/day Aged Garlic Extract; 90 days duration Wakunaga of America, Co. Ltd.**	T=26/56; C=28/56 RR 0.929 (95% CI, 0.632, 1.364; P=0.706)	*"Avg. number of symptoms per illness incident: T=11.9 (SEM 17); C=14 (SEM 17.6) P=0.536"* <br>*"total number of symptoms T=584; C=737; P<0.001"*	*"Number of days having symptoms: T=317; C=358; P=0.132"* <br>*"Number of work days missed T=8; C=19; P=0.035"*			*"After 45 d of consuming aged garlic extract, y&-T cells (P=0.039, n=56) and NK cells (P=0.043, n=56) were shown to proliferate better compared to placebo"*	Reported incidence of decrease in activity: T=15/48; C=38/58 RR 0.477 (95% CI, 0.301, 0.755; P=0.002) <br>*"total days of DIA: T=53;*

Study	Incidence of symptoms (# persons)	Severity of symptoms	Duration of symptoms	Adverse events	Laboratory evidence of infection	Biomarkers	Other resilience-type outcomes
							C=126 P<0.001"
**Vitamin A study outcomes**							
Children from India or China that are less than 10 years old given vitamin A (200000 IU) once every four to six months - studies vary from 6 months duration to 15 months duration **N=2 studies (n=1719 participants) – reported statistics in varying ways and came to different conclusions about the incidence rate of respiratory illnesses.**							
Chowdhury 2002[12] **2 ml vitamin A (200000 IU) given once every 4-6 months over 15 month period**	*"Incidence child/year for ARI T=0.288; C=0.361"*	*"No. episodes T=88; C=147* <br>*RR 0.80 (95% CI, 0.61, 1.04) P=0.09"*				*"following vitamin A supplementation on changes in conjunctival impression cytology (CIC) started after 2 months and thereafter, the prevalence of abnormal CIC in vitamin A supplemented children was significantly less (P<0.001)"*	
Chen 2013[11] **200000 IU vitamin A capsule (as retinol) just one time and tracked over 6 months**	Respiratory related illnesses T=32/95; C=37/104 RR 0.947 (95% CI 0.646, 1.388; P=0.780)	*"Number of respiratory related events T=262; C=284"*	*"T= 5.5 days; C=8 days"*				

Study	Incidence of symptoms (# persons)	Severity of symptoms	Duration of symptoms	Adverse events: estimated difference in the probability of experiencing the event	Laboratory evidence of infection	Biomarkers	Other resilience type outcomes
**Vitamin C study outcomes**<br><br>**N=3 studies (n=237 total participants) all throughout the winter months taking Vitamin C (1000 mg) - up to 3 months duration.**							
young men (18-35 y) with marginal vitamin C status during the peak of the cold season (January to April)							
Johnston 2014[14]<br>**1000 mg vitamin C, Twinlab C-500 CAPS) per day for 8 weeks**	T=7/15; C=11/13<br>RR 0.55 (95% CI, 0.306, 0.994; P=0.048); 45% less risk of developing symptoms when taking vitamin C	T=10.7+-6; C=18.6+-17.5; SMD= -0.623 (95% CI, -1.384, 0.137; P=0.108)<br><br>Number of colds: T=12; C=17; "(RR = 0.66, 95% CI [0.28, 1.55], *p* = 0.11"	"Cold duration was reduced 59% in the vitamin C vs placebo groups (−3.2 days, 95% CI [−7.0, 0.6]; p = 0.06)."<br><br>T=2.2+-1.4; C=5.4+-4.5;<br>MD -3.2 (95% CI, -5.84, -0.56 P=0.018)			*Fasting plasma vitamin C concentrations were raised significantly for the vitamin C group versus the placebo group at week 4 (41.3 ± 10.9 versus 30.8 ± 11.4 µmol/L; p = 0.02) and at week 8 (42.1 ± 10.4 versus 33.9 ± 10.9 µmol/L; p = 0.05).*	Impact on daily living mean difference of 0.2<br>T=2.6+-1.6; c=2.4+-2.1; SMD=-0.108 (95% CI, -0.851, 0.635; P=0.775)<br><br>*Physical activity increased slightly in the vitamin C group compared to the placebo group in the first four weeks of the trial (+6.2% to +7.5%; p ≥*

Study	Incidence of symptoms (# persons)	Severity of symptoms	Duration of symptoms	Adverse events: estimated difference in the probability of experiencing the event	Laboratory evidence of infection	Biomarkers	Other resilience type outcomes
							0.7); at weeks 5–6 and weeks 7–8, physical *activity increase more markedly in the vitamin C (+21.2%, 95% CI [−23.7, 66.1], p = 0.3 and +39.6%, 95% CI [−4.5, 83.7], p = 0.10, respectively)*<br><br>*Diet quality did not change significantly between groups at week 8*<br><br>*dietary vitamin C did not differ significantly between*

Study	Incidence of symptoms (# persons)	Severity of symptoms	Duration of symptoms	Adverse events: estimated difference in the probability of experiencing the event	Laboratory evidence of infection	Biomarkers	Other resilience type outcomes
							groups during the trial
Participants from the UK throughout the winter months (November –February)							
Van Straten 2002[15]<br>**1000 mg Ester-C per day for 60 days**	T=35/84; C=34/84<br>RR=1.029 (95% CI, 0.717, 1.479; P=0.875)	Number of cold episodes: T=37; C=50; P<0.05<br><br>significantly shorter duration of severe symptoms (1.8 vs 3.1 days, P< .03)	T=1.8 +-2.98; C=3.1+-4.65<br>Cold duration reduced 42% in vitamin c<br>MD -1.3 (95% CI, -3.012, 0.412; P=0.137)	T=9/84; C=11/84<br>RD= -0.024 (95% CI, -0.122, 0.074); P=0.634			
Competitive young swimmers under age 18 from Jerusalem who train at least 15 h/week/ conducted throughout winter months (December – February)							

Study	Incidence of symptoms (# persons)	Severity of symptoms	Duration of symptoms	Adverse events: estimated difference in the probability of experiencing the event	Laboratory evidence of infection	Biomarkers	Other resilience type outcomes
Constantini 2011[13]<br>**1 g/day vitamin C (ascorbic acid) prepared by Teva Pharmaceutical Industries Ltd., Netanya, Isreal over three winter months**		Total number of URI episodes per person year: T=12; C=12;<br><br>Number of URI episodes total: T=55; C=43<br><br>Total severity of symptoms: difference -16 (95% CI, -43, 11) in favor of Vitamin C<br><br>SMD=-0.240 (95% CI, -0.640, 0.161;P=0.240)<br><br>**Males only**: difference -40 (95% CI, -75, -6)<br><br>SMD= -0.678 (95% CI, -1.251, -0.105; P=0.02)	Total difference of URI episode days: T=6.9+-5.4; C=8.9+-7.8<br>MD= -2.0 days (95% CI, -4.6, 0.617; P=0.134)<br><br>**Males only**: T=5.5+-5.0; C=10.4+- 7.1<br>MD=-4.9 days (95% CI -8.2, -1.58)<br>P=0.004<br><br>Episodes: T=30; C=21				
**Vitamin D study outcomes**							
New Zealand older adults (25% levels <50 nmol/L) – 1.6 yrs (N=1; 5110 participants)							

Study	Incidence of symptoms (# persons)	Severity of symptoms	Duration of symptoms	Adverse events: estimated difference in the probability of experiencing the event	Laboratory evidence of infection	Biomarkers	Other resilience type outcomes
Camargo 2020[16]<br>**200,000 tapering to 100,000/month (3300 IU/d) Tishcon**	T=1882/2539;<br>C=1855/2517<br>RR=1.01 (95% CI, 0.97, 1.04; P=0.73)<br><br>*"there was no evidence of benefit among individuals with baseline 25(OH)D less than 50 nmol/L"*						
New Zealand adults (72.5 nmol/L) – 18 mos (N=1; 322 participants)							
Murdoch 2012[30]<br>**200,000 tapering to 100,000/month (3300 IU/d) Tishcon**	T=154/161; C=155/161<br>RR=0.99 (95% CI, 0.95, 1.04; P=0.78)<br><br>*"result remained unchanged when the analysis included winter season or baseline 25-OHD levels"*	*No episodes: T=593; C=611*<br><br>*Severity per episode, median (IQR); T= 171 (86-295); C= 183 (97-316); P=0.48*	*Median (25th percentile, d) duration of symptoms per episode*<br>*T=12 (8), n=366; C=12 (7); n=365; P=0.76*<br><br>*No. days missed work per episode*<br>*T=0.76 (1.25); C=0.76 (1.26) P=0.82*		*No. (%) of URTI episodes with positive swabs*<br>*T= 177 (51); C= 166 (49)*		
Australian adults (67.9 nmol/L) – 16 wks throughout the winter months (N=1; 34 participants)							

Study	Incidence of symptoms (# persons)	Severity of symptoms	Duration of symptoms	Adverse events: estimated difference in the probability of experiencing the event	Laboratory evidence of infection	Biomarkers	Other resilience type outcomes
Simpson 2015[33]<br>**20,000 IU/wk (3000 IU/d) Dartnells**	*Infection hazard (HR) for all URTI; T=1.11 (0.75, 1.65); C=1.00 (reference) P=0.62*	*No. episodes of RTI T=36; C=32*<br><br>*Average daily total infection symptom severity: T=-1.08 (-3.00, 0.85); C=5.28 (3.73, 6.82) P=0.27*	*Infection duration (HR) for URTI T=0.51 (-1.80, 2.81); C=4.87 (3.29, 6.45); P=0.67*				
Prediabetic adults (60 nmol/L) -5 yrs (N=1; 511 participants)							
Jorde 2016[25]<br>**20,000 IU/wk (3000 IU/d) Dekristol**	Number of subjects that develop at least one influenza like illness; T=119/256; C=97/255<br>RR=1.22 (95% CI, 1.00, 1.5; P=0.055						
Adults throughout winter months (82.5 nmol/L) – 1 year (N=1; 759 participants)							
Rees 2013[31]<br>**1000 IU/d**	number of participants reporting all seasons URTI episodes; T=303/399; C=276/360 RR=0.991 (95% CI, 0.915, 1.072; P=0.814)<br><br>number of participants reporting winter episodes; T=275/387; C=252/351	*Mean episodes per person-month (SD) All seasons: T=0.20 (0.26); C=0.23 (0.27); adjusted RR=0.94 (0.79, 1.11)*<br><br>*Winter: T=0.24 (0.30); C=0.27 (0.31); adjusted RR=0.93 (0.79, 1.09)*	*All seasons days of illness per person-month (SD); T=1.52 (2.98); C=1.56 (2.87); adjusted RR=1.03 (0.81, 1.31)*<br><br>*Winter: T=1.78 (3.33); C=1.68 (2.84); RR=1.13 (0.90, 1.43)*				

Study	Incidence of symptoms (# persons)	Severity of symptoms	Duration of symptoms	Adverse events: estimated difference in the probability of experiencing the event	Laboratory evidence of infection	Biomarkers	Other resilience type outcomes
	RR=0.990 (95% CI, 0.903, 1.084; P=0.825)						
Adults throughout winter months (58-63 nmol/L) up to 12 wks (N=2; 268 participants)							
de Gruijl 2012[18] **1000 IU/d Solgar Vitamins for 8 weeks**	T=19/37; C=22/33 RR=0.770 (95% CI, 0.519, 1.144; P=0.196)						
Li-Ng 2009[28] **2000 IU/d; 12 weeks**	T=28/78; C=29/70 RR=0.866 (95% CI, 0.577, 1.302; P=0.490)	No episodes; T=48; C=50<br><br>Severity of URI, range 1-5; T=2.6 (1.0); C=2.8 (1.2) SMD=-0.182 (95% CI, -0.505, 0.141; P=0.270)	Duration of URI, days T=5.4 (4.8); C=5.3 (3.1) MD=0.10 (95% CI, -1.49, 1.69; P=0.902)	T=38/78; C=34/70 RD=0.001 (95% CI, -0.160, 0.163; P=0.986)			
Japanese adults throughout winter months (75 nmol/L or less) – 16 wks (N=1; 252 participants)							
Shimizu 2018[32] **400 IU/d DSM Nutritional Products**	T=41/110; C=43/105 RR=0.910 (95% CI, 0.652, 1.271; P=0.581)	T=26.7 (11.6), n=41; C=31.2 (16.3), n=43 SMD=-0.317 (95% CI, -0.747, 0.114; P=0.149)	*[medians] T=10 days (7-12 days); C=13 days (10-19 days) P=0.061*	T=73/125; C=74/123 RD=-0.018 (95% CI, -0.140, 0.105; P=0.778)			*QOL score T=27.6 (17.2); C=31.3 (19.3); P=0.346*

Study	Incidence of symptoms (# persons)	Severity of symptoms	Duration of symptoms	Adverse events: estimated difference in the probability of experiencing the event	Laboratory evidence of infection	Biomarkers	Other resilience type outcomes
**British army recruits during training (25% sufficient) - 12 wks (N=1; 250 participants)**							
Harrison 2021[22]<br>**1090 (4wks), 460 IU (8 wks) Pure Encapsulations**<br><br>***Results combine vitamin D supplementation group and simulated sun light vitamin D group. The authors stated no difference between these groups when combined as "vitamin D supplementation"***		*Compared with placebo, vitamin D supplementation reduced the severity of peak URTI symptoms by 15% ; P < 0.05;*<br><br>*Participants beginning vitamin D supplementation with serum 25(OH)D <50 nmol·L $^{-1}$ had 21% lower peak URTI severity (P < 0.05)*	*Compared with placebo, vitamin D supplementation reduced the days with URTI by 36% (P < 0.05); P < 0.05;*<br><br>*Participants beginning vitamin D supplementation with serum 25(OH)D <50 nmol·L $^{-1}$ had 33% shorter average URTI duration (P = 0.05)*			*Supplementation did not affect salivary secretory immunoglobulin A or cathelicidin.*	
Young Finnish men during military training (78 nmol/L) – 6 mos (N=1; 164 participants)							
Laaksi 2010[27]<br>**400 IU/d Minisun**	T=39/80; C=54/84<br>RR=0.758 (95% CI, 0.576, 0.999; P=0.049)		*Mean days absent from duty; T=2.2 (3.2); C=3.0 (4.0); P=0.096*				
College male taekwondo athletes So. Korea (<50 nmol/L; M=31 nmol/L) throughout winter months – 4 wks (N=1; 26 participants)							

Study	Incidence of symptoms (# persons)	Severity of symptoms	Duration of symptoms	Adverse events: estimated difference in the probability of experiencing the event	Laboratory evidence of infection	Biomarkers	Other resilience type outcomes
Jung 2018[26]<br>**5000 IU/d BioTech**		T=7.7 (1.06), n=8; C=13.0 (1.60), n=9 SMD=-3.856 (95% CI, -5.464, -2.247; P=0.000)				*While the significant increase in SIgA was observed in both groups (p < 0.001), elevated salivary lactoferrin level in response to winter training was found only in the placebo group (p = 0.011).*	*QOL (score/day) T=7.0 (1.0); C=9.7 (2.27); P=0.093*
Adolescent competitive swimmers in Israel (60 nmol/L) training 15 hrs/wk during winter – 3 mos (N=1; 55 participants)							
Dubnov-Raz 2015[19]<br>**2000 IU/d CTS Chemical Industries**	T=11/14; C=11/12 RR=0.857 (95% CI, 0.621, 1.183; P=0.349)	T=4.7 (1.6), n=11; C=4.4 (1.4), n=11 SMD=0.20 (95% CI, -0.638, 1.037; P=0.641)	T=4.3 (1.6), n=11; C=5.1 (2.2), n=11 MD=-0.80 (95% CI, -2.41, 0.808; P=0.329)				
McMaster university students during peak rates of rhinovirus – 8 wks (N=1; 600 participants)							

Study	Incidence of symptoms (# persons)	Severity of symptoms	Duration of symptoms	Adverse events: estimated difference in the probability of experiencing the event	Laboratory evidence of infection	Biomarkers	Other resilience type outcomes
Goodall 2014[21]<br>**10,000 IU/wk (1428/d) EuroPharm**	T=70/258; C=80/234<br>RR=0.79 (95% CI, 0.61, 1.04; P=0.09)	*Complete case URTI episodes; T=70; C=80; P=0.09*<br><br>Symptom severity; T=218.6 (124.0)n=53; C=199.8 (108.1), n=53<br>SMD=0.162 (95% CI, -0.220, 0.543; P=0.406)	T=6.0 (1.6), n=53; C=6.2 (1.3), n=53<br>MD=-0.20 (95% CI, -0.755, 0.355; P=0.480)		T=26/256; C=44/233<br>RR=0.538 (95% CI, 0.343, 0.844; P=0.007)	*Viral load; T=5.51 (1.6); C=6.4 (1.2); P=0.036*	
Japanese high school students during influenza pandemic – 2 mos (N=1; 247 participants)							
Urashima 2014[35]<br>**2000 IU/d Zenyaku**	Over 2 months:<br>T=32/148; C=17/99<br>RR=1.259 (95% CI, 0.741, 2.140; P=0.394)<br><br>First month: T=11/148; C=11/99<br>RR= 0.67 (95% CI, 0.30, 1.48; P=0.32)		*Number of students absent T=68/148; C=38/99*<br><br>*Mean absences, d; T=1.7 (2.5); C=1.1 (1.9); P=0.14*				
Japanese school children throughout winter months – 4 mos (N=1; 430 participants)							
Urashima 2010[34]<br>**1200 IU/d Zenyaku**	Incidence of influenza a or b; T=57/167; C=59/167<br>RR=0.966 (95% CI, 0.720, 1.296; P=0.818)						

Study	Incidence of symptoms (# persons)	Severity of symptoms	Duration of symptoms	Adverse events: estimated difference in the probability of experiencing the event	Laboratory evidence of infection	Biomarkers	Other resilience type outcomes
Children in Denmark (56.7 nmol/L) throughout winter months – 20 wks (N=1; 130 participants)							
Hauger 2019[23] **400 or 800 IU/d**	*The risk of respiratory infections increased from baseline to endpoint within all groups with odds ratios of 3.32 (95% CI 1.27; 8.68), 2.22 (95% CI 0.79; 6.25), and 3.00 (95% CI 1.11; 7.85) in the placebo, 10 and 20 µg/day group, respectively (P values from 0.02 to 0.13). However, these increases in risk did not differ between groups (all P > 0.57).*					*a decreased LPS-induced IL-8 with placebo [– 438 (95% CI – 693; – 184) ng/L] was marginally avoided with 20 µg/day [– 109 (– 374; 157) ng/L] (P = 0.07)*	
Vietnamese children (67.5 nmol/L) – up to 1 yr (N=1; 1300 participants)							
Loeb 2019[29] **14,000 IU/wk (2000 IU/d) Ddrops**	influenza A or B or other respiratory illnesses; T=177/650; C=209/650 RR=0.847 (95% CI, 0.716, 1.002; P=0.053)				PCR laboratory confirmed influenza a or b; T=50/650; C=43/650 RR=1.163 (95% CI, 0.785, 1.722; P=0.452)		

Study	Incidence of symptoms (# persons)	Severity of symptoms	Duration of symptoms	Adverse events: estimated difference in the probability of experiencing the event	Laboratory evidence of infection	Biomarkers	Other resilience type outcomes
School children in Mongolia (30 nmol/L) – 3 yrs (N=1; 8851 participants)							
Ganmaa 2020[20]<br>**14,000 IU/wk (2000 IU/d) Tishcon**	T= 3783/4401; C= 3793/4418<br>RR=1.001 (95% CI, 0.984, 1.018; P=0.888)						
**Vitamin E study outcomes**<br><br>**N=1 study among seniors (>60 years old, n= 652 participants) with adequate nutritional status over 15 months to include three months of winter, concluding that 200 mg of Vitamin E did not show a favorable effect on incidence and severity of acute respiratory tract infections in well-nourished noninstitutionalized elderly individuals. "Instead we observed adverse effects of vitamin E on illness severity". (Data only taken from Vitamin E and placebo groups)**							
Graat 2002 and Van Amsterdam 2005[36,37]<br>**400 mg/dL per day of alpha-tocopheryl acetate; 15 months**	T=112/164; C=102/153<br>RR 1.024 (95% CI, 0.879, 1.194; P= 0.758)	*"Number of symptoms, median (IQR) T=6 (3-8); C=4 (3-8)"*<br><br>*"Number of episodes: T=280; C=230"*	*"Total illness duration, days, median (IQR) T=19 (10-30); C=14 (6-30)"*		T=13/29; C=10/21<br>RR 0.941 (95% CI, 0.515, 1.721; P=0.844)	*"vitamin E does not affect the level of DHEAS and neopterin"*	Restriction of activity: T=54/109; C=48/99<br>RR 1.022 (95% CI, 0.774, 1.349; P=0.879)
**Zinc study outcomes**							
School aged children up to age 15 y, living outside of the U.S. - up to 7 months duration to include the winter months<br>N=4 studies; (n=1355 children) taking 10-20 mg zinc per day in various different formulations or types of zinc							

Study	Incidence of symptoms (# persons)	Severity of symptoms	Duration of symptoms	Adverse events: estimated difference in the probability of experiencing the event	Laboratory evidence of infection	Biomarkers	Other resilience type outcomes
Kurugol 2006[38]<br>**zinc sulphate (15 mg of zinc) syrup supplied by Berko Ilac¸ Company, Turkey, once per day; 30 mg/day if symptoms occurred, up to 10 d duration; 7 months**	T=65/97; C=83/97; RR 0.783; (95% CI, 0.666, 0.921; P=0.003)	Mean number of colds per study child: T: 1.2+-1.4; C: 1.7+-1.2; P=0.003<br><br>Total events: T:121; C=160<br><br>Severity of symptoms at day 5: T=0.2 (4.9); C=0.4(5.9);<br>SMD= -0.037 (95% CI, -0.318, 0.245; P=0.789)	Duration of cold symptoms: T: 4.7+-0.8; C: 5.3+-0.7; MD= -0.60 (95% CI, -0.776, -0.424; P=0.0001)<br><br>Mean cold-related school absence T= 0.9 (2.1); C=1.3 (1.9) MD -0.40 (95% CI, -0.964, 0.164; P=0.164)	T=35/100; C=33/100 RD 0.02 (95% CI, -0.111, 0.151; P=0.765)			
Rerksuppaphol 2013[40]<br>**15 mg chelated zinc (zinc bis-glycinate - Qualimed, Thailand) tablet once daily; 3 months**	At least one symptom: T=42/50; C=41/50 RR1.024 (95% CI, 0.858, 1.223; P=0.790)<br><br>All three symptoms: T=6/50; C=13/50<br>RR 0.462 (95% CI, 0.191, 1.118; P=0.087)	Cold related school absence: T=13/50; C=14/50<br>RR 0.929 (95% CI, 0.487, 1.770; P=0.822)	*"Duration of at least 2 symptoms: median (IQR) T=0.0 (0-1.0); C=1.0 (0-5.3) P<0.01"*	T=0/50; C=1/50			
Richards 2006[41]<br>**20 mg zinc (zinc sulfate) given as 2.5 mL of a red strawberry-flavored syrup made in Lima, Peru by a Peruvian pharmaceutical Lab (Instituto Quimioterapico, Peru); 7 months**	NR per person	Number of episodes: T=39; C=39				*"Zinc group had significantly higher average plasma zinc concentrations than placebo group (P<0.01)"*	

Study	Incidence of symptoms (# persons)	Severity of symptoms	Duration of symptoms	Adverse events: estimated difference in the probability of experiencing the event	Laboratory evidence of infection	Biomarkers	Other resilience type outcomes
Vakili 2009[42]<br>**10 mg elemental zinc tablet (6 days a week); 5 month duration**	NR per person<br><br>"Average common cold occurrence T=1.7 (0.86); C=3.1 (0.55) P<0.001"	Max events: T=6; C=8	Days missing school: T=0.55 +-1.09; C=1.35+-1.79; MD -0.80 (95% CI, -1.211, -0.389; P<0.0001				
cadets during the cold and flu season (Oct-April) exposed to a number of stressful situations (basic training and academic life)<br>**N=1 study; (n=40 participants with mean age of 18) taking 15 mg capsule of zinc**							
Veverka 2009[43]<br>**15 mg capsule of zinc supplied through an "FDA licensed clinical specialty pharmacy (FL, USA); 7 months**	T=5/20; C=4/17<br>RR 1.063 (95% CI, 0.338, 3.338; P=0.917)	Number of symptom events: T=135/238; C=163/240<br>*"when comparing the subjects that had no self report symptoms during the study to those with symptoms, subjects in the Zinc group appreciated more weeks without any symptoms (p=0.01)"*				plasma zinc concentrations for both groups increased significantly (p = 0.04) but no effect was found between groups (p =0.87)	
seniors some of which were zinc-deficient and oxidatively stressed moreso compared to younger adults – up to 12 months duration<br>**N=1 study; (50 participants) taking 45 mg elemental zinc**							

Study	Incidence of symptoms (# persons)	Severity of symptoms	Duration of symptoms	Adverse events: estimated difference in the probability of experiencing the event	Laboratory evidence of infection	Biomarkers	Other resilience type outcomes
Prasad 2007[39] **zinc gluconate - 45 mg/day elemental zinc; 12 months**	T=7/24; C=22/25 RR 0.331 (95% CI, 0.175, 0.629; P=0.001)					*ex vivo generation of tumor necrosis factor and plasma oxidative stress markers were significantly lower in the zinc-supplemented than in the placebo group. Plasma zinc and phytohemagglu tin-induced interleukin 2 mRNA in isolated mononuclear cells were significantly higher in the zinc supplemented than in the placebo group.*	

RR = risk ratio (if RR<1, the risk in the DS group is less than the risk in the control group [1-RR=reduction in relative risk]); RD = risk difference (estimated difference in the probability of experiencing the event); SMD=standardized mean difference (negative indicates a reduction in symptom scores in favor of intervention; 0-0.2 considered small; 0.2-0.8 moderate; >0.8 large effect, used when scales are different across studies; MD= difference in means, used when

scales are the same, such as days with infection. For duration of symptoms, used events of illness, not the number of people so duration per event on average, negative indicates reduction in duration in favor of DS. Calculations were done using Comprehensive Meta-Analysis.

Table S6: Adverse event information

Adverse Events reported in evaluated studies	Adverse events found from searching case reports	Natural Medicines reporting of adverse events	CAERS database 2014-March 2020
**Echinacea**			
sleeplessness<br>and severe oral aphthous ulcers, which resolved spontaneously<br>while receiving treatment (evaluated as likely not to be related to treatment)<br>gastrointestinal side effects, diarrhea<br>Allergic and hypersensitivity reactions including rash or urticarial (in both groups)	• Allergic reaction[2,3,8]<br>• Appeared ill[9]<br>• Anorexia[6]<br>• Arthralgia/myalgia[2,3,9]<br>• Blisters (trunk, head, oral muscosa; maybe exacerbated by patients condition, cannot rule out URI contributed to his flare)[1]<br>• Blood levels effected[7,9]<br>• Burning sensation[2], Throat[3,8]<br>• Cardiac effects[2,7]<br>• Changes in stool[2,6]<br>• Chest tightness[2]<br>• Dark urine[6]<br>• Disorientation/Dizziness[2]<br>• Difficulty breathing/swallowing[2]<br>• Dry eyes/mouth[9]<br>• Edema, Facial[2] recurrent[8], Periorbital[2], Upper airway[2]<br>• Elevated LDH[7]<br>• Epistaxis[2]<br>• Erythema[8] nodosum, episodes[3]<br>• Eosinophilic granulocyte infiltration[5]<br>• Fatigue[2,4,5,6,9]<br>• Fever[3,7]<br>• Hepatitis, acute cholestatic autoimmune hepatitis ACAH)[2,5,6], Interface[5]	General: Orally, echinacea is well tolerated.<br><br>Most Common Adverse Effects: Orally: Abdominal pain, constipation, diarrhea, heartburn, nausea and vomiting, rashes, and stomach upset.<br><br>Serious Adverse Effects (Rare): Orally: Severe allergic reactions and hepatitis have been reported.	There are 26 CAERS reported incidents, categorized as: Hospitalization, Life threatening, Medically important, Other outcome, Patient visited ER, and Patient Visited Healthcare Provide.<br>Adverse events included: Abdominal Pain, Activities of daily living impaired, Alpha-1 anti-trypsin increased, Amylase increased, Aneurysm, Angioedema, Arteritis, Arthralgia, Autoimmune haemolytic anaemia, Cancer, Cardiac effects, Cerebrovascular accident, Coeliac disease, Changes in blood levels, Chest discomfort, Chills, Cognitive dysfunction/impairment (D/I), Cold sweat, Constipation, Decreased appetite, Dehydration, Developmental delay, Diabetes Mellitus, Diverticulum, Dizziness, Dysstasia, Ear, nose and throat examination abnormal, Eosinophil count decreased, Eye disorder, Faeces pale, Failure to thrive, Fatigue, Feeling abnormal, Feeling cold, Fever, Fluid retention, Gastritis, Gastrointestinal D/I, Gastroenteritis salmonella, Headache, Herbal toxicity, Hernia, Hyperhidrosis, Hypoaesthesia, Infantile spasms, International normalised ratio increased, Kidney D/I, Lipase increased, Liver injury, Loss of consciousness, Muscle spasms, Muscle twitching, Necrosis, Neutrophil count increased, Osteoarthritis, Pain, Pruritus generalised, Pyrexia, Respiratory D/I, Scab, Skin issues, Smooth muscle antibody positive, Sneezing, Stomach D/I, Thirst decreased, Tongue issues, Transferrin increased, Tremor, Ulcer, Unresponsive to stimuli, Urinary issues, Vaginal haemorrhage, Vitamin B6 increased, Vomiting, Wound

	• Headache[2,3]<br>• Hypersensitivity[2]<br>• Immune system impaired[5,9,10]<br>• Increased indirect bilirubin[7]<br>• Jaundice/Icteric skin[4,5,6]<br>• Liver function impairment[5,6,7]<br>• Muscle weakness, diffuse/generalized[9]<br>• Nausea[2]<br>• Portal lymphoplasmocytic infiltration[5]<br>• Rash[2,7]<br>• Renal function impaired[7], Acute renal failure[2]<br>• Sclera[4]<br>• Severe thrombocytopenia[7]<br>• Tender nodules[3]<br>• Unpleasant taste[3]<br>• Vasculitis[2]		

Echinacea Case Report References

1. Lee, A. N., & Werth, V. P. (2004). Activation of autoimmunity following use of immunostimulatory herbal supplements. *Archives of Dermatology*, *140*(6). https://doi.org/10.1001/archderm.140.6.723
    1.1. Dose: "Began taking an Echinacea supplement daily. He had never before taken an herbal supplement."
2. Mullins, R. J., & Heddle, R. (2002). Adverse reactions associated with echinacea: the Australian experience. *Annals of Allergy, Asthma, & Immunology*, *88*, 42–51.
    2.1. Dose: Case 1 – "patient swallowed 5 mL (approximately double the manufacturer's recommended amount) of a commercially prepared 40% alcohol in water solution of echinacea, further diluted in apple and black currant juice (according to the manufacturer's label). The amount ingested was equivalent to 3825 mg whole plant extract of Echinacea angustifolia and 150 mg dry root of E. purpurea." (Mullins 1998 Med J Australia)
    2.2. Dose: Case 3 – "An echinacea-containing tablet"
    2.3. Dose: Case 4 – "Ingestion of echinacea tablets"
    2.4. Dose: Case 5 – "Ingestion of echinacea tablets"
3. Lee Soon, S., & Crawford, R. I. (2001). Recurrent erythema nodosum associated with echinacea herbal therapy. *Journal of the American Academy of Dermatology*, *44*(2), 298–299. https://doi.org/10.1067/mjd.2001.112219
    3.1. Dose: "He routinely took Echinacea at the onset of influenza-like illnesses…"
4. Gabranis, I., Koufakis, T., Papakrivos, I., & Batala, S. (2015). Echinacea-associated acute cholestatic hepatitis. *Journal of Postgraduate Medicine*, *61*(3), 211. https://doi.org/10.4103/0022-3859.159430
    4.1. Dose: "15 days prior to presentation, he had developed a flu-like syndrome, for which he had received echinacea root tablets (600mg per day for 5 days) in order to stimulate his immune system"
5. Kocaman, O., Hulagu, S., & Senturk, O. (2008). Echinacea-induced severe acute hepatitis with features of cholestatic autoimmune hepatitis. *European Journal of Internal Medicine*, *19*(2), 148. https://doi.org/10.1016/j.ejim.2007.04.014

<table>
<tr><td colspan="4">5.1. Dose: "he had started taking Echinacea (Echinacea root tb; 1500 mg/day) after catching the cold"
6. Lawrenson, J. A., Walls, T., & Day, A. S. (2014). Echinacea-induced acute liver failure in a child. Journal of Paediatrics and Child Health, 50(10), 841–841. https://doi.org/10.1111/jpc.12726
6.1. Dose: "These were Kiwiherb Children's Echinature (3.5 mg echinacea in 5 mL solution), 0.5–1.0 mL daily for 2 weeks, and Healtheries Boost Immunity (containing vitamin C 1 g, zinc 7.5 mg and Echinacea 200 mg), half a tablet daily for 2 weeks. Total daily dose equated to 100.7 mg of echinacea."
7. George, L., Ioannis, E., Radostina, T., & Antonios, M. (2006). Severe thrombotic thrombocytopenic purpura (TTP) induced or exacerbated by the immunostimulatory herb echinacea. American Journal of Hematology, 81(3), 224–224. https://doi.org/10.1002/ajh.20531
7.1. Dose: "used an oral water–alcoholic extract of the herb Echinacea pallida for about a week"
8. Engebretsen, K. A., Johansen, J. D., & Thyssen, J. P. (2015). Herbal medicine as a cause of recurrent facial oedema. Contact Dermatitis, 72(5), 342–344. https://doi.org/10.1111/cod.12364
8.1. Dose: "Echinaforce, a herbal medicine intended to improve his immune system (herb and root extract from the plant Echinacea purpurae, in the plant family Asteraceae/Compositae)"
9. Logan, J. L., & Ahmed, J. (2003). Critical hypokalemic renal tubular acidosis due to Sjogren's syndrome: Association with the purported immune stimulant echinacea. Clinical Rheumatology, 22(2), 158–159. https://doi.org/10.1007/s10067-002-0671-4
9.1. Dose: "She had begun taking St John's Wort, echinacea and kava"
10. Kemp, D. E., & Franco, K. N. (2002). Possible Leukopenia Associated with Long-Term Use of Echinacea, 15(5), 417.
10.1 Dose: "For the past 8 weeks she had been taking vitamins C, E, and B complex, along with echinacea, ginkgo biloba, and calcium. She initially began taking echinacea when family members became ill with upper respiratory tract infections. The patient believed echinacea had prevented her from getting a cold and thus continued to take 450-mg capsules of the herb, three times daily for 2 months. This dose is typically recommended by manufactures and European physicians, but generally for only up to 6 weeks or less."</td></tr>
<tr><td colspan="4">Elderberry</td></tr>
<tr><td>Cold-like symptoms
fatigue</td><td>• Acute pancreatitis[1]
• Diffuse Abdominal pain, diffuse[1], persistent[1]</td><td>General: Orally, elderberry extracts prepared from ripe fruit seem to be well tolerated.

Most Common Adverse Effects: Orally: When adverse effects occur, they are likely due to ingestion of raw and unripe elderberries, or seeds, leaves, and other plant parts. Due to cyanogenic glycosides, these may cause nausea, vomiting, severe diarrhea, weakness, dizziness, numbness, and</td><td>There are 27 CAERS reported incidents, categorized as: Disability, Hospitalization, Life threatening, Medically important, Other outcome, Other seriousness, Patient visited ER, Patient Visited Healthcare Provider.
Adverse events included: Abdominal pain, Autoimmune haemolytic anaemia, Blood test abnormal, Cardiac impairment, Chills, Cognitive effects, Decreased appetite, Defaecation urgency, Diarrhoea, Dry mouth, Failure to thrive, Food poisoning, Gastrointestinal discomfort, Headache, Hypersensitivity, Hypoaesthesia, Ischaemic stroke, Lethargy, Loss of consciousness, Malaise, Menstruation delayed, Muscle spasms, Oral Allergy Syndrome, Pain, Pancreatitis, Plasma cell myeloma, Pyrexia, Respiratory effects, Skin effects/irritations, Vitamin B6 increased, Vitamin C decreased, Vitamin D decreased, Vomiting</td></tr>
</table>

		stupor. Cooking eliminates the toxin.	
**Elderberry Case Report References**			
1. Weissman, S., Lo, A., Patel, R., Mehta, T. I., Singh, V., Aziz, M., Belyayeva, A., Cherian, J., Amrutiya, V., Atoot, A., Hassan, A., Sotiriadis, J., Atoot, A., & Tabibian, J. H. (2019). An unusual culprit of drug-induced pancreatitis. *Digestive Diseases and Sciences*, *65*(5), 1549–1552. https://doi.org/10.1007/s10620-019-05864-4<br>1.1 Dose: “New homeopathic medication – black elderberry (Sambucol)”, 2-3 doses			
**Garlic**			
Rash<br>odor	• CRP elevated[3]<br>• Disappearance of body sensation below the T7 dermatome[5]<br>• Epistaxis[4]<br>• Eyes, haemorrhaged during surgery[1]<br>• International normalised ratio (INR), abnormal, decreased[6]<br>• Mass lesion in epidural space at T3–T5 levels[5]<br>• Pain[2,5]<br>• Paralysis of bilateral lower extremities[5]<br>• Renal haematoma (left kidney, 6x5x4 cm) after extracorporeal shock-wave lithotripsy (SWL)[2]<br>• Respiratory Issues/Dysfunction[3]<br>• Sensory deficit, lower extremity motor[5]<br>• Spontaneous spinal epidural hematoma (SSEH)[5]	General: Orally, garlic is generally well tolerated.<br><br>Most Common Adverse Effects: Abdominal pain, body odor, flatulence, malodorous breath, and nausea. Allergic reactions in sensitive individuals.<br><br>Serious Adverse Effects (Rare): Orally: Some case reports raise concerns about increased risk of bleeding with garlic.	There are 42 CAERS reported incidents, categorized as Death, Hospitalization, Life threatening, Medically important, Other outcome, Other Seriousness, Patient visited ER, Patient visited Healthcare provider, Required intervention.<br>Adverse events included: Abdominal pain, Anaphylactic reaction, Aphagia, Asthenia, Anaemia, Asthma, Appendicitis, Atrioventricular block, Changes in blood concentrations, Bilirubin urine, Bronchitis, Burning sensation, Cancer, Cardiac dysfunction, Cerebrovascular accident, Chapped lips, Chemical poisoning, Chest discomfort, Chills, Choking sensation, Chromaturia, Circulatory collapse, Cognitive issues, Cold sweat, Computerised tomogram abnormal, Convulsion, Cough, Decreased appetite, Dehydration, Diarrhoea, Diabetes mellitus, Discomfort, Diverticular perforation, Dizziness, Drug interaction, Dysphagia, Dysphonia, Dyspnoea, Dysstasia, Epitaxis, Eye pain/swelling, Feeling cold, Faeces discoloured, Fatigue, Feeling abnormal, Fluid retention, Foreign body, Foreign body trauma, GI & Stomach dysfunction, Glomerular filtration rate decreased, Glucose urine present, Hernia, Haemorrhage, Headache, Hepatomegaly, Hepatic pain, Hyperhidrosis, Hypersensitivity, Infarction, Insomnia, Lacrimation increased, Lip dry, Lip haemorrhage, Liver dysfunction, Loss of consciousness/Syncope, Lymphadenopathy, Malaise, Musculoskeletal stiffness, Muscle fatigue, Nasal congestion, Nausea, Oedema mouth, Oesophageal spasm, Oesophagitis, Odynophagia, Pain, Pallor, Pollakiuria, Pulmonary congestion, PO2 decreased, Positron emission tomogram abnormal, Photopsia, Pyrexia, Pneumonia, Respiratory arrest, Renal impairment, Rash, Renal cyst, Skin exfoliation, Throat tightness, Tremor, Tongue discolouration, Ultrasound scan abnormal, Urine analysis

			abnormal, Unresponsive to stimuli, Urinary tract infection, Vomiting, Weight decreased

Garlic Case Report References

1. Carden, S. M., Good, W. V., Carden, P. A., Good, R. M. (2002). Garlic and the strabismus surgeon. Clinical & Experimental Ophthalmology, 30(4), 303–304. https://doi.org/10.1046/j.1442-9071.2002.00540.x
    1.1. Dose: odourless garlic on a regular basis prior to surgery...the day prior to surgery she had consumed five tablets (approximately 5 g of equivalent fresh bulb).
    1.2. Patient: non-insulin dependent diabetes mellitus.
2. Gravas, S., Tzortzis, V., Rountas, C., Melekos, M. D. (2009). Extracorporeal shock-wave lithotripsy and garlic consumption: A lesson to learn. Urological Research, 38(1), 61–63. https://doi.org/10.1007/s00240-009-0242-0
    2.1. Dose: 600 mg tablets aged garlic extraction twice a day.
    2.2. Patient: Although, it is difficult to prove whether the haematoma was caused by the SWL itself or the consumption of herbal supplements, we believe that in our case garlic consumption significantly contributed to the occurrence of this extensive haematoma.
3. Suzuki, Y., Saito, J., Misa, K., Fukuhara, N., Fukuhara, A., Munakata, M. (2016). A case of black garlic-induced pneumonia as an adverse reaction. Allergology International, 65(3), 353–355. https://doi.org/10.1016/j.alit.2016.02.009
    3.1. Dose: She began taking black garlic.
    3.2. Patient: History of hypertension.
4. Shakeel, M., Trinidade, A., McCluney, N., Clive, B. (2010). Complementary and alternative medicine in epistaxis: A point worth considering during the patient's history. European Journal of Emergency Medicine, 17(1), 17–19. https://doi.org/10.1097/mej.0b013e32832b1679
    4.1. Dose: He admitted to using garlic and milk thistle tablets for the same duration as the aspirin.
    4.2. Patient: The patient said he had started taking 75 mg of aspirin daily for the past 2 weeks on his own initiative for perceived high blood pressure.

5. Kim, E. J., Ahn, J., Kim, S.-J. (2018). Spontaneous spinal epidural hematoma of the thoracic spine after Herbal Medicine: A case report. BMC Complementary and Alternative Medicine, 18(1). https://doi.org/10.1186/s12906-018-2354-y
    5.1. Dose: had been continuously taking herbal medicine containing black garlic for 8 weeks.
    5.2. Patient: A 60-year healthy female who was a hospital janitor…medical history, there was no recent trauma, no familial bleeding disorder, or no anticoagulation treatment. The patient's previous surgical history revealed an open discectomy procedure at L3 to L4 due to herniated intervertebral disc in 2011.
6. Pathak, A., Léger, P., Bagheri, H., Senard, J.-M., Boccalon, H., Montastruc, J.-L. (2003). Garlic interaction with fluindione: A case report. Therapies, 58(4), 380–381. https://doi.org/10.2515/therapie:2003060
    6.1. Dose: 600 mg/d.
    6.2 Patient: Drug-food interaction between garlic and fluindione. Dosages of concomitantly administered drugs (enalapril 20mg, furosemide 40mg, pravastatin 20mg) were unmodified. In November 2000, his INR suddenly dropped below its normal range (2.5) and stayed below 2 for 12 consecutive days, despite an increase in the OAC dosage.

**Vitamin A**			
Not described	• Abdominal distention, suggestive of ascites[8], moderate[2]<br>• Ascites[4]<br>• Changes in blood levels[3-5]<br>• Cheilosis[2]	General: Orally, vitamin A is generally well-tolerated at doses below the tolerable upper intake level (UL).	There are 16 CAERS reported incidents, categorized as: Hospitalization, Life threatening, Medically important, Patient visited ER, and Patient Visited Healthcare Provider.<br>Adverse events included: Abdominal pain, Alopecia, Bone pain, Brain/Cerebrovascular issues, Burning sensation, Cardiovascular

	• Chronic allograft nephropathy[3]<br>• Constipation[3]<br>• Enlarged spleen[1]<br>• Epistaxis[2]<br>• Focal intracellular cytoplasmic necrosis with apoptotic bodies in Kupffer cells[6]<br>• Haematochezia[3]<br>• Hair loss[2]<br>• Headache, generalised[7]<br>• Liver dysfunction[1,2,4,6,8]<br>• Malaise[3]<br>• Melena[3]<br>• Occular problems[7]<br>• Partial macular star appearance[7]<br>• Pruritus[6]<br>• Pseudotumour cerebri[7]<br>• Regression in language skills[2]<br>• Skin issues[2,6]<br>• Tremor[3]<br>• Unsteadiness of gait[3]<br>• Unsteady gait[2]<br>• Weight loss[3]<br>• Worsening balance[2]	Serious Adverse Effects (Rare): Orally: In very high doses, vitamin A can cause pseudotumor cerebri, pain, liver toxicity, coma, and even death.<br><br>Ages & Tolerable upper intake levels UL for Vitamin A based on IOM Guidelines: 12 mos (600 mcg), 1-3 yrs (600 mcg), 4-8 yrs (900 mcg), 9-13 yrs (1700 mcg), 14-18 yrs (2800 mcg), 19+ yrs (3000 mcg)	dysfunction, Changes in blood levels, Chest pain, Colitis ulcerative, Cough, Defecation urgency, Dehydration, Diverticulitis, Diplegia, Dizziness, Dysstasia, Diarrhea, Fall, Fatigue, Feeling hot, Gastrooesophageal reflux disease, Hypersensitivity, Immune system disorder, Irritability, Irritable bowel syndrome, Kidney dysfunction, Liver dysfunction, Loss of Consciousness, Malaise, Mouth irritations, Nausea, Ocular dysfunction, Overdose, Pneumonia, Photophobia, Porphyrin metabolism disorder, Pruritus, Pyrexia, Skin irritations, Speech disorder, Swelling, Throat tightness, Vomiting, Weight decreased

Vitamin A Case Report References

1. Castaño, G., Etchart, C., & Sookoian, S. (2006). Vitamin A toxicity in a physical culturist patient: A case report and review of the literature. *Annals of Hepatology*, *5*(4), 293–295. https://doi.org/10.1016/s1665-2681(19)31992-1
   1.1. Dose: History of increased vitamin A intake from natural source and supplementation of vitamin A 220,000 IU/d, and consumed steroidal anabolic drugs.
   1.2. Patient: 25 yo, M
2. Azzam, R., Dilley, K., Melin-Aldana, H., Alonso, E., & Sentogo, T. (2005). Clinical Quiz. *Journal of Pediatric Gastroenterology and Nutrition*, *41*(3), 363–365.
   2.1. Dose: Vitamin drops
   2.2. Patient: 29 mo, M
3. Cohen, E. P. (2004). Hypercalcaemia from non-prescription Vitamin A. *Nephrology Dialysis Transplantation*, *19*(11), 2929–2929. https://doi.org/10.1093/ndt/gfh437
   3.1. Dose: Dietary Supplement containing vitamin A, containing 7000 U b-carotene/tablet. Taken 4x/d for < 6 mos.
   3.2. Patient: 67 yo, white, M, underwent kidney transplant 6 yrs prior after kidney failure, medications included prednisone, mycophenolate mofetil, atorvastatin, labetalol, bumetanide, potassium chloride
4. García-Muñoz, P., Bernal-Bellido, C., Marchal-Santiago, A., Cepeda-Franco, C., Álamo-Martínez, J. M., Marín-Gómez, L. M., Suárez-Artacho, G., Castillo-Tuñón, J. M., Navarro-Morales, L., Padillo-Ruíz, F. J., & Gómez-Bravo, M. A. (2019). Liver cirrhosis from chronic hypervitaminosis a resulting in liver transplantation: A case report. *Transplantation Proceedings*, *51*(1), 90–91. https://doi.org/10.1016/j.transproceed.2018.03.141
   4.1. Dose: Vitamin A supplements
   4.2. Patient: 48 yo, M, medical history of congenital ichthyosiform erythroderma treated with vit A and oral corticosteroids
5. Lam, H. S., Chow, C. M., Poon, W. T., Lai, C. K., Chan, K. C., Yeung, W. L., Hui, J., Chan, A. Y., & Ng, P. C. (2006). Risk of vitamin A toxicity from candy-like chewable vitamin supplements for children. *Pediatrics*, *118*(2), 820–824. https://doi.org/10.1542/peds.2006-0167
   5.1. Dose: Same brand of jelly vitamin (2000 IU Vit A + 200 IU Vit D2/jelly), ~100-150 vit/3-5d
   5.2. Patient: 1) 5 yo, M; 2) 5 yo, M; 3) 30 mo M
6. Ramanathan, V. S., Hensley, G., French, S., Eysselein, V., Chung, D., Reicher, S., & Pham, B. (2010). Hypervitaminosis a inducing intra-hepatic cholestasis—a rare case report. *Experimental and Molecular Pathology*, *88*(2), 324–325. https://doi.org/10.1016/j.yexmp.2009.11.007
   6.1. Dose: Herbalife shake and 2 multivitamin tablets/12 years (3416 IU/d Vit A total).
   6.2. Patient: 46 yo, Caucasian, M, reported common bile duct structure, recent stent removal 15 days prior, history of B cell lymphoma, post-chemotherapy 3 yrs ago, in remission
7. Benzimra, J. D., Simon, S., Sinclair, A. J., & Mollan, S. P. (2014). Sight-threatening pseudotumour cerebri associated with excess vitamin A supplementation. *Practical Neurology*, *15*(1), 72–73. https://doi.org/10.1136/practneurol-2014-000934
   7.1. Dose: Admitted to using several dietary supplements for 2 mos, high dose multivitamin (Vit A 2400 ug; 8000 IU)
   7.2. Patient: 17 yo, F
8. Sheth, A., Khurana, R., & Khurana, V. (2008). Potential liver damage associated with over-the-counter vitamin supplements. *Journal of the American Dietetic Association*, *108*(9), 1536–1537. https://doi.org/10.1016/j.jada.2008.06.443
   8.1. Dose: 13,000 ug Vit A/d, for 2 yrs
   8.2. Patient: 59 yo, M, significant DM, dyslipidemia, obstructive sleep apnea

Vitamin C

Indigestion<br>Heartburn	• Acute renal failure[5]<br>• Acute tubular necrosis[5]	**G**eneral: Orally, vitamin C is well-tolerated.	There are 186 CAERS reported incidents, categorized as: Death, Disability, Hospitalization, Life threatening, Medically important,

	• Anorexia[3]<br>• Birefringent crystals consistent with calcium oxalate in lumen and cytoplasm of renal tubules[3]<br>• Confusion[3]<br>• Decreased urinary output[3]<br>• Elevated plasma glucose, fasting[1], postprandial[1]<br>• High urine oxalate excretion[2]<br>• High hyperoxaluria[2]<br>• Fatigue[3,4]<br>• Fibrosis[3]<br>• Headache, 6-day history[5]<br>• Hematuria[2]<br>• Hypodense lesion in left kidney suggestive of simple cyst[3]<br>• Increased cortical echogenicity of the kidneys[5]<br>• Increased iron in liver (hepatocytes & Kupffer cells)[4]<br>• Inflammation with lymphoplasmacytic infiltration[3]<br>• Intermittent left colic pain[2]<br>• Interstitial edema[3]<br>• Left ureteral stone[2]<br>• Lethargic but arousable[3]<br>• Massive oxalate deposition[5]<br>• Nausea[5], mild[2]<br>• Oxalate nephropathy[3]<br>• Pediatric urolithiasis[2]<br>• Vomiting[2,5]<br>• Word-finding difficulty[3]	Most Common Adverse Effects:<br>*Orally*: Abdominal cramps, esophagitis, heartburn, headache, osmotic diarrhea, nausea, vomiting. Kidney stones have been reported in those prone to kidney stones. Adverse effects are more likely to occur at doses above the tolerable upper intake level of 2 grams daily.<br><br>Serious Adverse Effects (Rare):<br>*Orally*: There have been rare case reports of carotid inner wall thickening after large doses of vitamin C.<br><br>Ages & Tolerable upper intake levels UL for Vitamin C based on IOM Guidelines: 12 mos NA, 1-3 yrs (400 mg), 4-8 yrs (650 mg), 9-13 yrs (1,200 yrs), 14-18 yrs (1,800 mg), 19+ yrs (2,000 mg)	Other outcome, Other seriousness, Patient visited ER, Patient visited healthcare provider.<br>Adverse events included: Abasia, Abnormal test results, Abscess, Accidental overdose, Allergic reaction, Alopecia, Appendix disorder, Arthralgia, Asthenia, Asthma, Bacteraemia, Biliary tract disorder, Blindness transient, Bronchitis, Burning sensation, Calcification metastatic, Cancer, Cardiovascular changes/dysfunction, Cellulitis, Cerebrovascular accident, Changes to blood/concentration levels, Chest discomfort, Chills, Coeliac disease, Cold sweat, Colitis ulcerative, Colon injury, Coma, Condition aggravated, Constipation, Convulsion, Deafness, Death, Decreased appetite, Defaecation urgency, Dehydration, Dental caries, Diabetes mellitus, Diabetic coma, Dizziness, Drug interaction, Dry mouth, Dysphonia, Dyspnoea, Dysstasia, Dysuria, Ear discomfort, Effects on eyes/vision, Escherichia, Faeces discoloured, Fall, Fatigue, Feeling abnormal, Feeling cold, Feeling hot, Flatulence, Flushing, Food interaction, Food poisoning, Foreign body, Foreign body trauma, Gait disturbance, Gallbladder disorder, Gallbladder non-functioning, Gastrointestinal effects (nausea/vomiting), Gout, Haematochezia, Haemorrhage, Hallucination, Head injury, Headache, Hospitalisation, Hyperhidrosis, Hypoaesthesia, Hypoaesthesia oral, Impaired work ability, Infections, Joint injury, Lacrimation increased, Laryngitis, Lethargy, Liver dysfunction, Loss of consciousness, Lymphadenopathy, Malaise, Metal poisoning, Movement disorder, Muscle injury/dysfunction, Myasthenia gravis, Nasal congestion, Nausea/Vomiting, Neuropathy peripheral, Overdose, Pain, Paraesthesia, Paraesthesia oral, Paralysis, Pharyngeal oedema, Pneumonia, Potentiating drug interaction, Presyncope, Productive cough, Prostatomegaly, Protein total increased, Proteinuria, Psychological effects, Pulmonary sarcoidosis, Pyrexia, Quantitative, Rectal haemorrhage, Renal effects/dysfunction, Respiratory effects, Restless legs syndrome, Retching, Retinal scar, Salmonellosis, Sensation of foreign body, Sinusitis, Skin reactions/irritations, Small intestinal obstruction, Sneezing, Somnolence, Sputum increased, Swelling, Temporal arteritis, Tension headache, Throat & Swallowing problems, Tinnitus, Tongue coated, Tongue discolouration, Tooth fracture, Tooth loss,

<table>
<tr><td></td><td></td><td></td><td>Tremor, Unresponsive to stimuli, Ureteric obstruction, Urinary tract disorder, Urine protein, Urticaria, Vaginal haemorrhage, Vasculitis, Vocal cord disorder, Vocal cord paralysis, Weight decreased, Weight increased</td></tr>
<tr><td colspan="4">Vitamin C Case Report References

1. Branch, D. R. (1999). High-dose vitamin C supplementation increases plasma glucose. Diabetes Care, 22(7), 1218–1219. https://doi.org/10.2337/diacare.22.7.1218
1.1. Dose: 4,500 mg synthetic unsweetened Vitamin C product/d for 5 yrs
1.2. Patient: 49 yo M, slight-to-moderately obese,
2. Chen, X., Shen, L., Gu, X., Dai, X., Zhang, L., Xu, Y., & Zhou, P. (2014). High-dose supplementation with vitamin C–induced pediatric urolithiasis: The first case report in a child and literature review. Urology, 84(4), 922–924. https://doi.org/10.1016/j.urology.2014.07.021
2.1. Dose: 3 g/d Vit C effervescent tablets since 3 yo
2.2. Patient: 9 yo, M
3. Lin, W. V., Turin, C. G., McCormick, D. W., Haas, C., & Constantine, G. (2018). Ascorbic acid-induced oxalate nephropathy: A case report and discussion of pathologic mechanisms. CEN Case Reports, 8(1), 67–70. https://doi.org/10.1007/s13730-018-0366-6
3.1. Dose: Vitamin C supplements, 2 g/d.
3.2. Patient: 69 yo, M, history of benign prostate hyperplasia (BPH), and small bowel resection for strangulated Meckel diverticulum. “It is likely that oxalate nephropathy developed due to excessive supplement use, exacerbated by BPH and past small bowel resection.
4. Mallory, M. A., Sthapanachai, C., & Kowdley, K. V. (2003). Iron overload related to excessive vitamin C intake. Annals of Internal Medicine, 139(6), 532. https://doi.org/10.7326/0003-4819-139-6-200309160-00032
4.1. Dose: 325 mf ferrous sulfate 2x/d, and 15,000 mg Vit C/d
4.2. Patient: 61 yo, F. “Therefore, we feel that hepatic iron accumulation in our patient was due solely to the combination of vitamin C and iron intake.”
5. Mashour, S., Turner, J. F., & Merrell, R. (2000). Acute renal failure, oxalosis, and Vitamin C Supplementation. Chest, 118(2), 561–563. https://doi.org/10.1378/chest.118.2.561
5.1. Dose: Vitamin C tablets (500 mg), 4-5x/d. “On further inquiry, the patient was able to recall that he had begun taking even larger numbers of vitamin C tablets a few days prior to the onset of symptoms, up to 10 tablets daily (5,000 mg) due to a recent upper respiratory infection.”
5.2. Patient: 31 yo, African-American, M</td></tr>
<tr><td colspan="4">Vitamin D</td></tr>
<tr><td>Chest pain – “unrelated to study medication”
Common cold and stomach ache – not connected to study medication
Symptomatic hypercalcemia - nausea and epigastric discomfort</td><td>• Abdominal tenderness[7]
• Altered mental staus[8,10]
• Anemia[11]
• Balance difficulty[8]
• Calcium imbalance[1-14]
• Cardiovascular effects[1-4,7,10]
• Chronic gastric reflux symptoms[7]
• Chronic inflammation[13]
• Colic[1,3], occassional[1]
• Confusion[8]</td><td>General: Orally, vitamin D is well-tolerated.

Serious Adverse Effects (Rare):
Orally: Excessive doses can lead to vitamin D toxicity with symptoms of hypercalcemia, and also</td><td>There are 637 CAERS reported incidents, categorized as: Death, Disability, Hospitalization, Life threatening, Medically important, Other outcome, Other seriousness, Patient visited ER, Patient visited healthcare provider, Required intervention.
Adverse events included: Abdominal discomfort, Abnormal drug reactions, Abnormal test results, Accidental overdose, Activities of daily living impaired, Adhesion, Allergy/Immune related responses, Alopecia, Antineutrophil cytoplasmic antibody increased, Aphagia, Appendicectomy, Appendix disorder, Asphyxia, Back issues, Balance/Walking disorders, Basedow’s disease, Blood</td></tr>
</table>

	• Constipation[1,3,4,7,9]<br>• Creatinine elevated[6,8,14]<br>• Decreased frequency of stools[12]<br>• Dehydration[5,9]<br>• Dermatologic reactions[4,13]<br>• Diarrhea[9]<br>• Diminished muscle tone[12]<br>• Dry mouth[8]<br>• Elevated vitamin D[3,4,6,7,10-12,14]<br>• Epilepticus[8]<br>• Fat necrosis[13]<br>• Fatigue[4,5,7-12,14]<br>• Foot drop[10]<br>• Headache[8], sporadic frontal[4]<br>• Hemiplegia[8]<br>• Hyperphosphatemia[11]<br>• Hypertriglyceridaemia[3]<br>• Hypervitaminosis[2]<br>• Hypervitaminosis A[6]<br>• Hypoactive bowel sounds[7]<br>• Hypotrophic muscles[4]<br>• Intra-abdominal abcess[13]<br>• Irritability[9]<br>• Ischaemic colitis with colonic perforation[13]<br>• Muscle weakness[5]<br>• Nausea/Vomiting[2,3,5,7,9,14]<br>• Nephrolithiasis[3]<br>• Intact[6] Parathyroid hormone (PTH) low/suppressed[3,5,7-12,14]<br>• Pain[1,3-7]<br>• Phosphate elevated[6,8]<br>• Polydipsia[2,14]<br>• Postictal state[8]<br>• Renal damage[2-6,13,14]<br>• Retinol elevated[6]	sometimes azotemia and anemia.<br><br>Ages & Tolerable upper intake levels UL for Vitamin D based on IOM Guidelines: 0-6 mos (25 mcg, 1000 IU), 7-12 mos (38 mcg/1500 IU), 1-3 yrs (63 mcg/2500 IU), 4-8 yrs (75 mcg/3000 IU), 9+ yrs (100 mcg/4000 IU)	concentration changes, Blood count abnormal, Blood disorder, Blood thyroid stimulating hormone increased, Body height decreased, Brain mass, Brain natriuretic peptide increased, Breast engorgement, Breath odour, Broken bone, Burn oesophageal, Burning sensation, Calcification of muscle, Calciphylaxis, Cancer, Cardiovascular effects/impact, Cerebrovascular accident, Chemical poisoning, Chest discomfort, Childhood disintegrative disorder, Chills, Choking/Cough, Cholecystitis acute, Cholelithiasis, Coeliac disease, Cognitive impact, Colitis, Concussion, Condition aggravated, Consciousness, Constipation, Contusion, Convulsion, Crepitations, Crohn's disease, CSF immunoglobulin increased, Cystitis, Dandruff, Deafness, Death, Defaecation urgency, Dehydration, Dermatologic effects, Diabetes mellitus, Discomfort, Diverticular perforation, Diverticulitis, Dyskinesia, Dysphagia, Ear disorder, Effects on bones, Effects on muscle/tendon, Ejaculation disorder, Electrolyte imbalance, Encephalitis, Encephalomalacia, Encephalopathy, Endocrine disorder, Energy increased, Enzyme abnormality, Enzyme level increased, Epilepsy, Epistaxis, Esophagial changes, Facial rash/swelling, Faeces discoloured, Faeces hard, Failure to thrive, Fatigue, Febrile neutropenia, Feeling abnormal, Feeling cold, Feeling hot, Feeling jittery, Fluctuations in appetite/weight, Fluid overload, Food allergy, Food poisoning, Foreign body, Gallbladder disorder, Gastrointestinal impact/effects, Gastrooesophageal reflux disease, General physical health deterioration, Gout, Haematochezia, Haemorrhage, Haemorrhoids, Hair growth abnormal, Hair texture abnormal, Head injury, Head titubation, Headache, Hernia, Hiccups, Homonymous, Hospitalisation, Hot flush, Hyperaesthesia, Hyperhidrosis, Hypersensitivity, Hyperthyroidism, Hypoacusis, Hypoaesthesia, Hyponatraemia, Hypophosphataemia, Hypothyroidism, Hypoxia, Impact on sleep, Impaired work ability, Increased upper airway secretion, Infantile colic, Infantile spasms, Infection, Influenza, Infrequent bowel movements, Intentional overdose, International normalised ratio decreased, Liver damage/impact, Lung disorder, Malaise, Mass, Metabolic acidosis, Milk-alkali syndrome, Miscellaneous, Mobility decreased, Motor developmental delay, Motor dysfunction, Mucous stools, Multi-organ failure, Multiple sclerosis relapse, Myalgia, Myoclonus, Nasal

	• Sepsis[13]<br>• Urinary changes[2,3,4,8,9,11,14]<br>• Vitamin D intoxication[3,4,5,6,7,8,9,14]<br>• Weight loss/Poor eating[1,4,9,12]		congestion, Nausea, Nerve injury, Nervous system disorder, Neutrophil percentage increased, Norepinephrine increased, Normal pressure hydrocephalus, Ocular impact, Onychoclasis, Oral impact/effects, Osteopathic impact, Overdose, Oxygen saturation decreased, Pain, Painful defaecation, Pancreatic enzymes increased, Pancreatitis acute, Paraesthesia, Paralysis, Pericardial effusion, Prostatomegaly, Protein total decreased, Protein total increased, Pulmonary hypertension, Pyrexia, Red blood cell sedimentation rate increased, Renal damage, Repetitive speech, Respiratory acidosis, Respiratory effects, Retching, Rhinorrhoea, Seizure like phenomena, Sinus congestion, Speech disorder, Swelling, Systemic lupus erythematosus, Thirst, Thrombosis, Tinnitus, Transient ischaemic attack, Tremor, Ulcer, Ureteric obstruction, Urinary dysfunction, Vasogenic cerebral oedema, Vericose vein ruptured, Viith nerve paralysis, Vaginal irritation, Wheezing, White blood cell count increased, X-ray abnormal

Vitamin D Case Report References

1. Barrueto, F., Wang-Flores, H. H., Howland, M. A., Hoffman, R. S., & Nelson, L. S. (2005). Acute vitamin D intoxication in a child. *Pediatrics*, *116*(3). https://doi.org/10.1542/peds.2004-2580
   1.1. Dose: 1 ampule of Raquiferl, 600,000 IU Vit D for 4 days = 2,400,000
   1.2. Patient: 2 yo M
2. Benemei, S., Gallo, E., Giocaliere, E., Bartolucci, G., Menniti-Ippolito, F., Firenzuoli, F., Mugelli, A., & Vannacci, A. (2013). It's time for new rules on Vitamin D Food Supplements. *British Journal of Clinical Pharmacology*, *76*(5), 825–826. https://doi.org/10.1111/bcp.12134
   2.1. Case 1: 34 yo F, osteoporosis, atopic dermatitis, coeliac disease (316,800 IU day^{-1}, 4 mos)
   2.2. Case 2: 57 yo M, hypertension (158,400 IU day^{-1}, 11 mos)
   2.3. Case 3: 47 yo M, (422,400 IU day^{-1}, 5 mos)
3. Conti, G., Chirico, V., Lacquaniti, A., Silipigni, L., Fede, C., Vitale, A., & Fede, C. (2014). Vitamin D intoxication in two brothers: Be careful with dietary supplements. *Journal of Pediatric Endocrinology and Metabolism*, *27*(7-8). https://doi.org/10.1515/jpem-2013-0220
   3.1. Case 1: 12 yo M, (Label: 1.5 ug/capsule Vit D3, 0.24 mg Vit A, 5 mg Vit E, 14 ug Vit K, 3.6 mg Vit F; Analysis: 1060+/-49 ug Vit D = 42,400 IU Vit D/capsule, took 6 pills/d = 254,400 IU)
   3.2. Case 2: 15 yo, M (5 capsules/d for 2 wks = 3,180,000 IU)
4. de Paula, A. L., Gonzaga, W. P., Oliveira, L. M., Feibelmann, T. C., & Markus, J. (2020). Exogenous intoxication by non-prescribed use of vitamin D, a case report. *BMC Geriatrics*, *20*(1). https://doi.org/10.1186/s12877-020-01614-8
   4.1. Dose: Various vitamin complexes, including cholecalciferol 1x/d for 6 mos
   4.2. Patient: 64 yo, F
5. De Vincentis, S., Russo, A., Milazzo, M., Lonardo, A., De Santis, M. C., Rochira, V., Simoni, M., & Madeo, B. (2021). How much vitamin D is too much? A case report and review of the literature. *Endocrine, Metabolic & Immune Disorders - Drug Targets*, *21*(9), 1653–1659. https://doi.org/10.2174/1871530320666201007152230

5.1. Dose: “mean daily 130,000 UI”. “Cholecalciferol supplementation was started with an initial oral dose of 70,000 UI/day…supplementation was progressively increased as follows: 100,000 UI/day from March 2015 to July 2015, then 140,000 UI/day to April 2016, and finally up to 160,000 UI/day.”
5.2. Patient: 56 yo F, with multiple sclerosis, and history of iron deficiency anemia, hypertension, menopause, and chronic autoimmune thyroiditis

6. Granado-Lorencio, F., Rubio, E., Blanco-Navarro, I., Perez-Sacristan, B., Rodriguez-Pena, R., Garcia Lopez, F.J. (2012). Hypercalcemia, hypervitaminosis A and 3-epi-25-OH-D3 levels after consumption of an “over the counter” vitamin D remedy. A case report. *Food and Chemical Toxicology, 50,* 2106-2108.
   6.1. Dose: Vitamin D supplement, Cholecalciferol, 600,000 IU (15 mg), 1 vial/d for 4 wks
   6.2. Patient: 51 yo F, “history of surgical intervention for the extraction of a staghorn lithiasis on the left kidney…medications included atenolol and hydrocholorothiazide”
7. Houghton, C. C., & Lew, S. Q. (2020). Long-term hypervitaminosis D-induced hypercalcaemia treated with glucocorticoids and bisphosphonates. *BMJ Case Reports*, *13*(4). https://doi.org/10.1136/bcr-2019-233853
   7.1. Dose: 50,000 IU Vit D/d for ~7 months
   7.2. Patient: 25 yo healthy, M, with Wolff Parkinson White syndrome, status post accessory pathway ablation, diagnosis of vitamin D deficiency
8. Kaptein, S., Risselada, A. J., Boerma, E. C., Egbers, P. H. M., & Nieboer, P. (2010). Life-threatening complications of vitamin D intoxication due to over-the-counter supplements. *Clinical Toxicology*, *48*(5), 460–462. https://doi.org/10.3109/15563650.2010.486382
   8.1. Case 1: 75 yo F (“total daily dose of 3.75 ug (150 IU) vitamin D3, according to the directions”; “100-1,000 times higher than that stated on the label”)
   8.2. Case 2: 73 yo F, hypertension, hysterectomy, and medications including atenolol/chloorthalidon, bisacodyl, oxazapam (“100-1,000 times higher than that stated on the label”)
9. Ketha, H., Wadams, H., Lteif, A., & Singh, R. J. (2015). Iatrogenic vitamin D toxicity in an infant – a case report and review of literature. *The Journal of Steroid Biochemistry and Molecular Biology*, *148*, 14–18. https://doi.org/10.1016/j.jsbmb.2015.01.022
   9.1. Dose: “Instead on one drop the mother was administering approximately a dropper full every day for a period of 2 months; which she assumed totaled to about a 100 IU…vitamin D3 content of the supplement was found to be threefold higher (6000 IU of D/drop) than listed on the label (2000 IU)…resulted in a daily vitamin D dose of 50,000 IU for two months”
   9.2. Patient: 4 mos F
10. Kim, S., Stephens, L. D., & Fitzgerald, R. L. (2017). How much is too much? Two contrasting cases of excessive vitamin D supplementation. *Clinica Chimica Acta*, *473*, 35–38. https://doi.org/10.1016/j.cca.2017.08.004
   10.1. Case 1: 75 yo M, history of atrial fibrillation, coronary artery disease, heart failure, thyroid cancer, and bladder cancer (prescribed 50,000 IU ergocalciferol (vitamin D2) and 500 mg calcium-citrate for 1 yr)
   10.2. Case 2: 60 yo F, (consumed 40,000 IU of cholecalciferol (vitamin D3) daily for 10+ mos…”she independently increased her vitamin D3 intake from 2000 IU/day to 40,000 IU/day.)
11. Leu, J. P., Weiner, A., & Barzel, U. S. (2008). Vitamin D toxicity: Caveat emptor. *Endocrine Practice*, *14*(9), 1188–1190. https://doi.org/10.4158/ep.14.9.1188
   11.1. Dose: Five milliliters of Soladek (dietary supplement) [contains] 600,000 IU of vitamin D…
   11.2. Patient: 60 yo F Dominican, history of osteoarthritis
12. Smollin, C., & Srisansanee, W. (2014). Vitamin D toxicity in an infant: Case files of the University of California, San Francisco Medical Toxicology Fellowship. *Journal of Medical Toxicology*, *10*(2), 190–193. https://doi.org/10.1007/s13181-013-0365-0
   12.1. Dose: “”her vitamin D prescription called for 1 mL (400 IU vitamin D per mL) to be taken daily. The vitamin D supplement purchased online was a concentrated for containing 400 IU per drop.”
   12.2. Patient: 2 mos F, no significant past medical history
13. Storan, E. R., O’Gorman, S. M., Murphy, A., & Laing, M. (2016). Case report of calciphylaxis secondary to calcium and vitamin D3 supplementation. *Journal of Cutaneous Medicine and Surgery*, *21*(2), 162–163. https://doi.org/10.1177/1203475416668162
   13.1. Dose: Vitamin D3 supplementation for 4 mos

13.2. Patient: 64 yo F, normal renal function, fractured right femoral neck "with a dynamic hip screw and injection of 300 000 IU Vit D3 followed by daily calcium and vitamin D3 supplementation…she developed sepsis , ischaemic colitis with colonic perforation, and an intra-abdominal abscess requiring a total colectomy with stoma formation and acute kidney injury requiring 1 week of haemodialysis.<br>14. Van, K., Lai, F. Y., Ling, J., & Wood, C. (2018). The latest scoop: A rare case of vitamin D toxicity. *The American Journal of Medicine*, *131*(3). https://doi.org/10.1016/j.amjmed.2017.09.043<br>14.1. Dose: 4 scoops of protein bodybuilding supplement, containing 400 IU of vitamin D and 1200 mg of calcium over the last 3 mos<br>14.2. Patient: 22 yo M, no medical history or medications			
**Vitamin E**			
Participants were more likely to experience symptoms of respiratory illness	• Acute right hemiparesis[1]<br>• Anemia, mild[1]<br>• Aneurysms (small)[1]<br>• Dysarthria[1]<br>• Focal basal vacuolar changes[2]<br>• Headache[1], severe[1]<br>• Hemorrhages, Intracerebral (ICH)[1], Cryptogenic[1], Thalamic[1], Left thalamic[1], subcortical (thalamic) intraparenchymal[1], intraventricular[1], 4th ventricle[1], mild ventriculomegaly[1], subcortical without underlying pathology[1]<br>• Hypervitaminosis E[1]<br>• Infarcts, small multifocal[1]<br>• Left leg weakness[1]<br>• Lethargy[1]<br>• Macules, pruritic, purpuric, multiple, erythematous, non-palpable, on the dorsal aspects of her feet[2]<br>• Pigmented purpura[2]<br>• Pigmented purpuric dermatoses[2]<br>• Superficial perivascular lymphocytic infiltrate with extravasated red blood cells, hemosiderin deposits within the papillary dermis[2]<br>• a-tocopherol elevated[1], Serum[1]	General: Orally, vitamin E is generally well-tolerated.<br><br>Serious Adverse Effects (Rare):<br>*Orally*: Bleeding hemorrhagic stroke, cardiovascular complications.<br><br>Ages & Tolerable upper intake levels UL for Vitamin E based on IOM Guidelines: 1-3 yrs (200 mg), 4-8 yrs (300 mg), 9-13 yrs (600 mg), 14-18 yrs (800 mg), 19+ yrs (1,000 mg)	There are 48 CAERS reported incidents, categorized as: Disability, Hospitalization, Life threatening, Medically important, Other outcome, Other seriousness, Patient visited ER, and Patient Visited Healthcare Provider.<br>Adverse events included: Abasia, Abdominal pains, Abnormal faeces, Abortion spontaneous, Amnesia, Aphasia, Arthralgia, Asthenia, Asthma, Autism, Back pain, Blood disorder, Burning sensation, Cancer, Cardiovascular effects, Cataract, Cerebrovascular accident, Changes in blood concentrations, Chest discomfort, Chest pain, Chills, Choking, Cold sweat, Colitis ulcerative, Colon injury, Constipation, Cough, Deafness, Defaecation urgency, Dehydration, Dermatologic reactions, Diarrhoea, Diverticulitis, Dizziness, Dyspepsia, Dyspnoea, Dysstasia, Feeling abnormal, Feeling cold, Flatulence, Flushing, Foetal exposure during pregnancy, Foreign body, Frequent bowel movements, Gait disturbance, Gallbladder disorder, Gallbladder non-functioning, Gastric infection, Gastritis, Gastroenteritis viral, Gastrooesophageal reflux disease, General symptom, Haemorrhage, Headache, Hepatitis, Hepatobiliary scan abnormal, Hospitalisation, Hyperhidrosis, Hypersensitivity, Hypoaesthesia, Hypoaesthesia oral, Immune system disorder, Impaired work ability, Infection, Inflammation, Intestinal haemorrhage, Irritable bowel syndrome, Liver reactions, Liver function test abnormal, Local swelling, Loss of consciousness, Malaise, Movement disorder, Muscle spasms, Nausea, Obstructive airways disorder, Ocular reactions/dysfunction, Oral allergy syndrome, Oropharyngeal pain, Pain, Pain in extremity, Paraesthesia, Parkinson's disease, Pharyngeal oedema, Pleuritic pain, Pneumonia, Porphyrin metabolism disorder, Proteinuria, Pruritus, Pruritus generalised, Pyrexia, Quantitative, Rectal haemorrhage,

			Renal reactions, Respiratory arrest, Rhinorrhoea, Somnolence, Speech disorder, Stool analysis abnormal, Sunburn, Swelling, Throat tightness, Tongue discolouration, Transient global amnesia, Tremor, Unresponsive to stimuli, Urinary tract infection, Urine protein, Varicose vein, Vasculitis, Vertigo, Vomiting, Weight decreased, Weight increased

Vitamin E Case Report References

1. Le, N. K., Kesayan, T., Chang, J. Y., & Rose, D. Z. (2020). Cryptogenic intracranial hemorrhagic strokes associated with hypervitaminosis E and acutely elevated α-tocopherol levels. *Journal of Stroke and Cerebrovascular Diseases*, *29*(5), 104747. https://doi.org/10.1016/j.jstrokecerebrovasdis.2020.104747
    1.1. Case 1: 60 yo, M (400 IU/d), diet-controlled hyperlipidemia, mild ulcerative colitis, prehypertensive, took probiotic supplements.
    1.2. Case 2: 74 yo, M (400 IU/d), former smoker, mild hypertension, diabetic, coronary artery disease status post bypass, "claimed strict adherence with antihypertensive regimen" to include atenolol, telmisartan, and terazosin.
    1.3. Case 3: 79 yo, F, (400 IU/d), history of migraines, mild hypertension and hypothyroidism (controlled with Rx), was also taking fish oil, lutein-zeaxanthin
2. Unal, E., & Ergül, G. (2015). Pigmented purpuric dermatosis after taking a dietary supplement. *Cutaneous and Ocular Toxicology*, *35*(3), 260–262. https://doi.org/10.3109/15569527.2015.1094488
    2.1. Dose: 330.4 g vitamin E, with parsley concentrate (Petroselinum crispum), and 15 ug selenium
    2.2. Patient: 54 yo, F, treating hypothyroidism with levothyroxine sodium

**Zinc**			
mild gastrointestinal discomfort<br>Bad taste	• Anemia[2,3]<br>• Cellular bone marrow with active megakaryopoiesis[2]<br>• Convulsions, tonic-clonic[1]<br>• Cytoplasmic vacuoles[2]<br>• Dyspnea[3]<br>• Edema, mild pitting pedal[3]<br>• Epigastric "rising sensation"[1]<br>• Erythroid hyperplasia[2]<br>• Fatigue[2,3]<br>• Hypocupremia[2], zinc-induced [3]<br>• Increased serum ferritin & iron stores[3]<br>• Leukopenia[2,3]<br>• Neutropenia[2]<br>• Pale[3]<br>• Reticulocyte count low[2]<br>• Seizures, complex partial[1]<br>• Sideroblasts, ringed[2,3], non-ringed[3]	General: Orally, zinc is well tolerated in doses below the tolerable upper intake level (UL), which is 40 mg daily for adults.<br><br>Most Common Adverse Effects:<br>*Orally*: Abdominal cramps, diarrhea, metallic taste, nausea and vomiting (dose-related).<br><br>Serious Adverse Effects (Rare):<br>*Orally*: There have been cases of acute renal tubular necrosis, interstitial	There are 249 CAERS reported incidents, categorized as: Death, Disability, Hospitalization, Life threatening, Medically important, Other outcome, Other seriousness, Patient visited ER, and Patient Visited Healthcare Provider.<br>Adverse events included: Abasia, Abdominal pains, Abnormal test results, Abortion spontaneous, Acne, Acquired immunodeficiency syndrome, Adrenal disorder, Adrenal insufficiency, Adverse reaction, Alopecia, Aphasia, Apparent death, Appendicitis, Arthralgia, Arthropathy, Asthenia, Ataxia, Back pain, Bacterial infection, Balance disorder, Body height decreased, Brain natriuretic peptide increased, Burning sensation, Cancer, Candida infection, Cardiovascular effects, Cerebellar syndrome, Cerebrovascular accident, Changes in blood levels, Chest discomfort, Chest pain, Chills, Chromaturia, Coeliac disease, Collagen disorder, Colon injury, Colonic abscess, Concussion, Condition aggravated, Constipation, Contusion, Convulsion, Cystitis, Death, Decreased vibratory sense, Dehydration, Dermatologic effects, Diabetes, Diarrhoea, Discomfort, Disease

	• Swelling, lower extremeties[3]<br>• Unconscious staring spell (1-2 min)[1]<br>• Vacuolization in cell cytoplasm in erythroid and myeloid series[3]<br>• Zinc levels increased[3]	nephritis, neurological complications, severe vomiting, and sideroblastic anemia after zinc overdose.<br><br>Ages & Tolerable upper intake levels UL for Zinc based on IOM Guidelines: 0-6 mos (4 mg), 7-12 mos (5 mg), 1-3 yrs (7 mg), 4-8 yrs (12 mg), 9-13 (23 mg), 14-18 yrs (34 mg), 19+ yrs (40 mg)	recurrence, Dizziness, Drug abuse, Drug interaction, Dysphonia, Dysstasia, Dysuria, Ear infection, Ear pain, Epilepsy, Erectile dysfunction, Erythema, Eye swelling, Feeling abnormal, Feeling cold, Feeling hot, Flank pain, Flushing, Foreign body, Gait disturbance, GI/Stomach reactions/issues, Glossodynia, Groin pain, Haematemesis, Haemorrhage, Hallucination, Head injury, Headache, Hepatic impact, Hospitalisation, Hot Flush, Hyperacusis, Hypervigilance, Hypoaesthesia, Hypopnoea, Ill-defined disorder, Immune/Allergic response, Impact on sleep/energy, Impaired healing, Infection, Influenza like illness, Intraductal proliferative breast lesion, Jaundice, Localised infection, Loss of consciousness, Loss of proprioception, Lyme disease, Malaise, Meniere's disease, Metal poisoning, Muscle spasms, Muscle spasticity, Musculoskeletal disorder, Musculoskeletal pain, Myelopathy, Nasopharyngitis, Nausea, Neck pain, Neuralgia, Neuropathy peripheral, Nervous system disorder, Night sweats, Ocular/Visual impact, Oedema mouth, Oral/Mouth effects, Oral allergy syndrome, Oropharyngeal pain, Osteoarthritis, Overdose, Pain, Pallor, Paraesthesia, Paralysis, Parkinson's disease, Pnemonia, Postmenopausal haemorrhage, Presyncope, Psychological effects/reactions, Pyrexia, Renal reactions, Respiratory effects, Rhinorrhoea, Sarcoidosis, Sneezing, Stomatitis, Swelling, Therapeutic response unexpected, Thyroid disorder, Tremor, Upper limb fracture, Urinary tract infection, Urge incontinence, Weight/Consumption changes, Wound hemorrhage

Zinc Case Report References

1. Green, A., & Weaver, D. (2008). Potential proconvulsant effects of oral zinc supplementation–a case report. *NeuroToxicology*, *29*(3), 476–477. https://doi.org/10.1016/j.neuro.2008.03.008
   1.1. Dose: 64 mg elemental zinc/d (i.e. 240 mg zinc gluconate 2x/d)
   1.2. Patient: 28 yo, M with 16 year history of seizures treated with phenytoin (300 mg/d)
2. Salzman, M. B., Smith, E. M., & Koo, C. (2002). Excessive oral zinc supplementation. *Journal of Pediatric Hematology/Oncology*, *24*(7), 582–584. https://doi.org/10.1097/00043426-200210000-00020
   2.1. Dose: 50 mg zinc/d increased to 100 mg 3x/d.
   2.2. Patient: 17 yo M, with acne
3. Ramadurai, J., Shapiro, C., Kozloff, M., & Telfer, M. (1993). Zinc abuse and sideroblastic anemia. *American Journal of Hematology*, *42*(2), 227–228. https://doi.org/10.1002/ajh.2830420217
   3.1. Dose: Zinc sulfate 100 mg x 6/d

3.2. Patient: 36 yo, F

The CAERS database was searched January 1, 2014 – March 31, 2020, first sorting by 'Product Type' and then 'Description'. Only SUSPECT Product Types and Vit/Min/Prot/Unconv Diet(Human/Animal) were included. Medra Preferred Terms and Outcomes were recorded. It is important to note the following: "*The presence of an adverse event report in CAERS does not mean the FDA has determined that the product listed was the cause of the event. Information from the reports is included in its original form and reports not always contain sufficient information for FDA to determine whether there is a correlation between the reported event and use of the product.*" https://www.fda.gov/food/compliance-enforcement-food/cfsan-adverse-event-reporting-system-caers

PubMed was searched with the search string: [dietary ingredient name] and (adverse effects) with the Filters: Case Reports, Humans, English, from 2001/1/1 – 2021/1/1, and included only dietary supplement (i.e. non-food, etc.) studies.

Dietary Recommended Allowance and Upper Limit References

**Ingredient**	**Recommended Daily Allowance**	**Tolerable Upper Limit**
Elderberry	N/A	N/A
Echinacea	N/A	N/A
Garlic	N/A	N/A
Vitamin A[1]	0-6 mos: 400 mcg RAE (1,333 IU retinol)<br>7-12 mos: 500 mcg RAE (1,667 IU retinol)<br>1-3 yrs: 300 mcg RAE (1,000 IU retinol)<br>4-8 yrs: 400 mcg RAE (1,333 IU retinol)<br>9-13 yrs: 600 mcg RAE (2,000 IU retinol)<br>14+ yrs: 700 mcg RAE (F) (2,333 IU retinol); 900 mcg RAE (M) (3,000 IU retinol)	0-3 yrs: 600 mcg (2,000 IU retinol)<br>4-8 yrs: 900 mcg (3,000 IU retinol)<br>9-13 yrs: 1,700 mcg (5,667 IU retinol)<br>14-18 yrs: 2,800 mcg (9,333 IU retinol)<br>19+ yrs: 3,000 mcg (10,000 IU retinol)
Vitamin C	0-6 mos: 40 mg<br>7-12 mos: 50 mg<br>1-3 yrs: 15 mg<br>4-8 yrs: 25 mg<br>9-13 yrs: 45 mg<br>14-18 yrs: 65 mg (F); 75 mg (M)<br>19+ yrs: 75 mg (F); 90 mg (M)	0-12 mos: "Not possible to establish"<br>1-3 yrs: 400 mg<br>4-8 yrs: 650 mg<br>9-13 yrs: 1,200 mg<br>14-18 yrs: 1,800 mg<br>19+ yrs: 2,000 mg

Vitamin D[2]	0-12 mos: 10 mcg (400 IU)<br>1-70 yrs: 15 mcg (600 IU)<br>70+ yrs: 20 mcg (800 IU)	0-6 mos: 25 mcg (1,000 IU)<br>7-12 mos: 38 mcg (1,500 IU)<br>1-3 yrs: 63 mcg (2,500 IU)<br>4-8 yrs: 75 mcg (3,000 IU)<br>9+ yrs: 100 mcg (4,000 IU)
Vitamin E (a-tocopherol)	0-6 mos: 4 mg<br>7-12 mos: 5 mg<br>1-3 yrs: 6 mg<br>4-8 yrs: 7 mg<br>9-13 yrs: 11 mg<br>14+ yrs: 15 mg	1-3 yrs: 200 mg<br>4-8 yrs: 300 mg<br>9-13 yrs: 600 mg<br>14-18 yrs: 800 mg<br>19+ yrs: 1,000 mg
Zinc	0-6 mos: 2 mg<br>7-12 mos: 3 mg<br>1-3 yrs: 3 mg<br>4-8 yrs: 5 mg<br>9-13 yrs: 8 mg<br>14-18 yrs: 9 mg (F);11 mg (M)<br>19+ yrs: 8 mg (F);11 mg (M)	0-6 mos: 4 mg<br>7-12 mos: 5 mg<br>1-3 yrs: 7 mg<br>4-8 yrs: 12 mg<br>9-13 yrs: 23 mg<br>14-18 yrs: 34 mg<br>19+ yrs: 40 mg

Footnotes:

1. To convert IU to mcg RAE, use the following: 1 IU retinol = 0.3 mcg RAE (https://ods.od.nih.gov/factsheets/VitaminA-HealthProfessional/)
2. RDAs for vitamin D are listed in both micrograms (mcg) and international units (IU); 1 mcg vitamin D = 40 IU. (https://ods.od.nih.gov/factsheets/VitaminD-HealthProfessional/)

References cited

1. Jawad M, Schoop R, Suter A, Klein P, Eccles R. Safety and efficacy profile of echinacea purpurea to prevent common cold episodes: A randomized, double-blind, placebo-controlled trial. *Evid Based Complement Alternat Med.* 2012;2012:841315.
2. Ogal M, Johnston SL, Klein P, Schoop R. Echinacea reduces antibiotic usage in children through respiratory tract infection prevention: A randomized, blinded, controlled clinical trial. *Eur J Med Res.* 2021;26(1):33.
3. O'Neil J, Hughes S, Lourie A, Zweifler J. Effects of echinacea on the frequency of upper respiratory tract symptoms: A randomized, double-blind, placebo-controlled trial. *Ann Allergy Asthma Immunol.* 2008;100(4):384-388.
4. Sperber SJ, Shah LP, Gilbert RD, Ritchey TW, Monto AS. Echinacea purpurea for prevention of experimental rhinovirus colds. *Clin Infect Dis.* 2004;38(10):1367-1371.
5. Tiralongo E, Lea RA, Wee SS, Hanna MM, Griffiths LR. Randomised, double blind, placebo-controlled trial of echinacea supplementation in air travellers. *Evid Based Complement Alternat Med.* 2012;2012:417267.
6. Turner RB, Bauer R, Woelkart K, Hulsey TC, Gangemi JD. An evaluation of echinacea angustifolia in experimental rhinovirus infections. *N Engl J Med.* 2005;353(4):341-348.
7. Tiralongo E, Wee SS, Lea RA. Elderberry supplementation reduces cold duration and symptoms in air-travellers: A randomized, double-blind placebo-controlled clinical trial. *Nutrients.* 2016;8(4):182.
8. Josling P. Preventing the common cold with a garlic supplement: A double-blind, placebo-controlled survey. *Adv Ther.* 2001;18(4):189-193.
9. Nantz MP, Rowe CA, Muller CE, Creasy RA, Stanilka JM, Percival SS. Supplementation with aged garlic extract improves both nk and γδ-t cell function and reduces the severity of cold and flu symptoms: A randomized, double-blind, placebo-controlled nutrition intervention. *Clin Nutr.* 2012;31(3):337-344.
10. Percival SS. Aged garlic extract modifies human immunity. *Journal of Nutrition.* 2016;146(2):433S-436S.
11. Chen K, Chen XR, Zhang L, et al. Effect of simultaneous supplementation of vitamin a and iron on diarrheal and respiratory tract infection in preschool children in chengdu city, china. *Nutrition.* 2013;29(10):1197-1203.
12. Chowdhury S, Kumar R, Ganguly NK, Kumar L, Walia BN. Effect of vitamin a supplementation on childhood morbidity and mortality. *Indian J Med Sci.* 2002;56(6):259-264.
13. Constantini NW, Dubnov-Raz G, Eyal BB, Berry EM, Cohen AH, Hemilä H. The effect of vitamin c on upper respiratory infections in adolescent swimmers: A randomized trial. *Eur J Pediatr.* 2011;170(1):59-63.
14. Johnston CS, Barkyoumb GM, Schumacher SS. Vitamin c supplementation slightly improves physical activity levels and reduces cold incidence in men with marginal vitamin c status: A randomized controlled trial. *Nutrients.* 2014;6(7):2572-2583.
15. Van Straten M, Josling P. Preventing the common cold with a vitamin c supplement: A double-blind, placebo-controlled survey. *Adv Ther.* 2002;19(3):151-159.
16. Camargo CA, Sluyter J, Stewart AW, et al. Effect of monthly high-dose vitamin d supplementation on acute respiratory infections in older adults: A randomized controlled trial. *Clin Infect Dis.* 2020;71(2):311-317.

17. Scragg R, Waayer D, Stewart AW, et al. The vitamin d assessment (vida) study: Design of a randomized controlled trial of vitamin d supplementation for the prevention of cardiovascular disease, acute respiratory infection, falls and non-vertebral fractures. *J Steroid Biochem Mol Biol.* 2016;164:318-325.
18. de Gruijl FR, Pavel S. The effects of a mid-winter 8-week course of sub-sunburn sunbed exposures on tanning, vitamin d status and colds. *Photochem Photobiol Sci.* 2012;11(12):1848-1854.
19. Dubnov-Raz G, Rinat B, Hemilä H, Choleva L, Cohen AH, Constantini NW. Vitamin d supplementation and upper respiratory tract infections in adolescent swimmers: A randomized controlled trial. *Pediatr Exerc Sci.* 2015;27(1):113-119.
20. Ganmaa D, Uyanga B, Zhou X, et al. Vitamin d supplements for prevention of tuberculosis infection and disease. *N Engl J Med.* 2020;383(4):359-368.
21. Goodall EC, Granados AC, Luinstra K, et al. Vitamin d3 and gargling for the prevention of upper respiratory tract infections: A randomized controlled trial. *BMC Infect Dis.* 2014;14:273.
22. Harrison SE, Oliver SJ, Kashi DS, et al. Influence of vitamin d supplementation by simulated sunlight or oral d3 on respiratory infection during military training. *Med Sci Sports Exerc.* 2021;53(7):1505-1516.
23. Hauger H, Ritz C, Mortensen C, et al. Winter cholecalciferol supplementation at 55°n has little effect on markers of innate immune defense in healthy children aged 4-8 years: A secondary analysis from a randomized controlled trial. *Eur J Nutr.* 2019;58(4):1453-1462.
24. Mortensen C, Damsgaard CT, Hauger H, et al. Estimation of the dietary requirement for vitamin d in white children aged 4-8 y: A randomized, controlled, dose-response trial. *Am J Clin Nutr.* 2016;104(5):1310-1317.
25. Jorde R, Sollid ST, Svartberg J, Joakimsen RM, Grimnes G, Hutchinson MY. Prevention of urinary tract infections with vitamin d supplementation 20,000 iu per week for five years. Results from an rct including 511 subjects. *Infect Dis (Lond).* 2016;48(11-12):823-828.
26. Jung HC, Seo MW, Lee S, Kim SW, Song JK. Vitamin d_3 supplementation reduces the symptoms of upper respiratory tract infection during winter training in vitamin d-insufficient taekwondo athletes: A randomized controlled trial. *Int J Environ Res Public Health.* 2018;15(9).
27. Laaksi I, Ruohola JP, Mattila V, Auvinen A, Ylikomi T, Pihlajamäki H. Vitamin d supplementation for the prevention of acute respiratory tract infection: A randomized, double-blinded trial among young finnish men. *J Infect Dis.* 2010;202(5):809-814.
28. Li-Ng M, Aloia JF, Pollack S, et al. A randomized controlled trial of vitamin d3 supplementation for the prevention of symptomatic upper respiratory tract infections. *Epidemiol Infect.* 2009;137(10):1396-1404.
29. Loeb M, Dang AD, Thiem VD, et al. Effect of vitamin d supplementation to reduce respiratory infections in children and adolescents in vietnam: A randomized controlled trial. *Influenza Other Respir Viruses.* 2019;13(2):176-183.
30. Murdoch DR, Slow S, Chambers ST, et al. Effect of vitamin d3 supplementation on upper respiratory tract infections in healthy adults: The vidaris randomized controlled trial. *Jama.* 2012;308(13):1333-1339.
31. Rees JR, Hendricks K, Barry EL, et al. Vitamin d3 supplementation and upper respiratory tract infections in a randomized, controlled trial. *Clin Infect Dis.* 2013;57(10):1384-1392.
32. Shimizu Y, Ito Y, Yui K, Egawa K, Orimo H. Intake of 25-hydroxyvitamin d3 reduces duration and severity of upper respiratory tract infection: A randomized, double-blind, placebo-controlled, parallel group comparison study. *J Nutr Health Aging.* 2018;22(4):491-500.

33. Simpson S, van der Mei I, Stewart N, Blizzard L, Tettey P, Taylor B. Weekly cholecalciferol supplementation results in significant reductions in infection risk among the vitamin d deficient: Results from the cipris pilot rct. *BMC Nutr.* 2015;1:doi: 10.1186/2055-0928-1181-1187.

34. Urashima M, Segawa T, Okazaki M, Kurihara M, Wada Y, Ida H. Randomized trial of vitamin d supplementation to prevent seasonal influenza a in schoolchildren. *Am J Clin Nutr.* 2010;91(5):1255-1260.
35. Urashima M, Mezawa H, Noya M, Camargo CA, Jr. Effects of vitamin d supplements on influenza a illness during the 2009 h1n1 pandemic: A randomized controlled trial. *Food Funct.* 2014;5(9):2365-2370.
36. Graat JM, Schouten EG, Kok FJ. Effect of daily vitamin e and multivitamin-mineral supplementation on acute respiratory tract infections in elderly persons: A randomized controlled trial. *Jama.* 2002;288(6):715-721.
37. van Amsterdam J, van der Horst-Graat J, Bischoff E, Steerenberg P, Opperhuizen A, Schouten E. The effect of vitamin e supplementation on serum dhea and neopterin levels in elderly subjects. *Int J Vitam Nutr Res.* 2005;75(5):327-331.
38. Kurugöl Z, Akilli M, Bayram N, Koturoglu G. The prophylactic and therapeutic effectiveness of zinc sulphate on common cold in children. *Acta Paediatr.* 2006;95(10):1175-1181.
39. Prasad AS, Beck FW, Bao B, et al. Zinc supplementation decreases incidence of infections in the elderly: Effect of zinc on generation of cytokines and oxidative stress. *Am J Clin Nutr.* 2007;85(3):837-844.
40. Rerksuppaphol S, Rerksuppaphol L. A randomized controlled trial of chelated zinc for prevention of the common cold in thai school children. *Paediatr Int Child Health.* 2013;33(3):145-150.
41. Richard SA, Zavaleta N, Caulfield LE, Black RE, Witzig RS, Shankar AH. Zinc and iron supplementation and malaria, diarrhea, and respiratory infections in children in the peruvian amazon. *Am J Trop Med Hyg.* 2006;75(1):126-132.
42. Vakili R, Vahedian M, khodaei G, Mahmoudi M. Effects of zinc supplementation in occurrence and duration of common cold in school aged children during cold season: A double-blind placebo-controlled trial. *Iran J Pediatr.* 2009;19(4):376-380.
43. Veverka DV, Wilson C, Martinez MA, Wenger R, Tamosuinas A. Use of zinc supplements to reduce upper respiratory infections in united states air force academy cadets. *Complement Ther Clin Pract.* 2009;15(2):91-95.
